# SHOCKING! College professor reveals hidden secrets of news headlines. Can you guess? Examining the efficacy of clickbait detection training and news literacy

**DOI:** 10.1371/journal.pone.0359401

**Published:** 2026-11-05

**Authors:** Ben Wasike

**Affiliations:** Department of Communication, University of Texas Rio Grande Valley, Texas, United States of America; Max Planck Institute for Solid State Research, GERMANY

## Abstract

This study used a survey and an experiment to examine how news literacy and personal locus, which is a competency adjacent to news literacy, affected clickbait detection and engagement with clickbait via clicking, reading, and sharing clickbait headlines and stories. The survey measured the effect of news literacy and the four psychometric personal locus variables (reflective thinking, the tolerance for ambiguity, signal detection, and the drive for information) on detection and engagement with clickbait. The pre- and post-test experiment with a control group measured the effect of clickbait detection training on detection and engagement. Overall, news literacy improved detection and reduced engagement, as did signal detection. Reflective thinking performed likewise via correlation but showed marginal main effects. The tolerance for ambiguity was inversely correlated with engagement, but not with detection, and it only reduced clicking marginally among the individual engagement variables. While the drive for information was not correlated with detection, it increased engagement, thus contradicting extant research. The experiment showed that while clickbait detection training reduced engagement with clickbait, it improved detection only when clickbait headlines were considered, and not when both clickbait and non-clickbait headlines were considered. The author recommends that news literacy training incorporate aspects of personal locus improvement and clickbait detection training.

## Introduction

The kicker in the title above is facetiously written as a clickbait headline. These are catchy and exaggerated headlines written with the sole purpose of baiting readers into clicking on them [1]. Such headlines use forward-referencing by suggestively mentioning aspects of the related story in advance to create curiosity and anticipation among readers, who may feel compelled to click the headline and read the story to satisfy their curiosity [2,3]. Clickbait headlines also carry emotionally charged words as well as question-based references that further increase curiosity [4–6]. The title above contains an emotionally charged word, “shocking!”, a forward-reference that does not specify the subject in question, “college professor,” and a question-based reference, “can you guess?” It is also an example of deceptive clickbait because none of what it claims appears in this paper.

Studying clickbait is important because 40% of news readers rarely delve past the headline [7]. On social media, 75% of shared news links are forwarded to others without the sender clicking on or reading the linked stories [8]. Additionally, most news readers prefer to click on short, simple headlines [9]. All these conditions favor clickbait. As I discuss later, clickbait negatively affects readers, but research suggests that news literacy is a potential deterrent. Lacking, however, is a healthy body of research on the effect of news literacy on clickbait detection. Therefore, this study examines whether news literacy improves clickbait detection and whether news literacy reduces the likelihood of engaging with clickbait via clicking on such headlines, reading the accompanying stories, and sharing them with others. The study also considers personal locus, a psychometric measure of media literacy, that is also associated with news literacy [10–12] and misinformation susceptibility [13,14]. Personal locus refers to a person’s control over their exposure to media and their awareness of the effects of that exposure [12].

As I discuss later, not only is news literacy a subset of media literacy, but it is also associated with the four personal locus variables under study. These variables are reflective thinking, signal detection, the tolerance for ambiguity (TFA), and the drive for information. Because little research has examined the association among these variables, especially in the context of clickbait detection, this study uniquely adds to knowledge. The study also uses a pre- and post-test experiment with a control group to examine the effect of clickbait detection training on clickbait detection and engagement with clickbait. Therefore, this study examines whether news literacy, personal locus, and clickbait detection training affect clickbait detection and engagement with it.

### The effects of clickbait

Clickbait may be deceptive or non-deceptive. Deceptive clickbait refers to headlines that misrepresent the true meaning of the accompanying stories [15]. Deceptive clickbait differs from the non-deceptive type in that the deceptive headlines have little to no congruity with the related story, thus causing disappointment for the reader [15]. Whether deceptive or not, clickbait’s main function is to create curiosity and suspense [3,6]. Clickbait headlines may also appear alongside visual flags [16]. These are thumbnails with striking imagery accompanying the headline, and their primary function is to attract attention by standing out against a field of competing images [17]. Broadly defined, curiosity is a feeling of being deprived of information and the ensuing desire for that information [18,19]. This information gap then motivates people to engage in information-seeking activities to reduce this deprivation [18].

Clickbaiters create this deprivation by inserting forward-references within the headlines [2,3]. Inserting emotionally charged words within the headlines is another tactic that clickbait uses to create suspense and curiosity [6]. In a discussion of the semantics of clickbait, Jodłowiec [15] gives examples of headlines such as *“Why You’ve Never Heard of This Top Travel Destination”* (p. 422) and *“The Tragic Real-Life Story of Tom Hanks”* (p. 427). The first headline uses the article “this” to create suspense and curiosity about the travel destination. The second headline uses “tragic” as an emotionally laden word to arouse curiosity about a famous actor. The first headline is a common marketing ploy used by clickbaiters, and the second leads to a rambling tabloid story, not about tragedy, but about the actor’s unsuccessful projects as well as the family issues he faced in his early childhood.

Not all clickbait is deceptive, as some news agencies use clickbait to boost traffic to their sites. In their analysis of forward-referencing in clickbait headlines, Blom and Hansen [2] give examples such as, *“He loves Beatles, menthol cigs..and longs for muscles like Van Damme”* (p. 88). The pronoun “he” is the forward-reference, and a curious reader may click the headline to find out the person’s identity. In this case, it was North Korea’s Kim Jong-Un. Other examples they give are *“This Amazing Kid Died. What He Left Behind is Wondtacular”* and *“This is an A-minus paper?”* (p. 88). The second headline uses emotionally charged words, and the third uses a question-based declaration. These headlines were published by news outlets like the *BBC*, *The Sun*, and *Upworthy*.

Regardless of the technique used or the deceptive nature of the headline, clickbait is consequential. Although some studies report mixed results regarding the effect of clickbait on readers [20,21], other studies report discernible effects. For one, continuous exposure to clickbait increases the believability of such headlines [22]. Additionally, these effects may occur in the presence of warnings, especially among readers with little knowledge about clickbait [23]. Generally, forward-references are more common among headlines found on commercial and tabloid media sites than among non-commercial and non-tabloid media sites [2]. Such headlines are also more likely to omit information than traditional headlines. In turn, this omission increases the chances of readers sharing the headlines [24]. This is important because research shows that headlines written with forward-references elicit more clicks and attention than those without [25]. However, clickbait headlines with forward-references also reduce the perceived credibility of the accompanying stories [1].

Even though question-based clickbait headlines arouse more curiosity than traditional headlines without questions [26], they elicit three negative effects that traditional headlines do not [4]. One, such headlines elicit perceptions of untrustworthiness, confusion, impropriety, and a lack of credibility and seriousness. Two, they increase the anticipation that the news articles accompanying the headlines will elicit the same negative reactions. Three, question-based clickbait headlines reduce engagement with the news via commenting, sharing, or posting reactions such as likes. This may explain why stories linked to such headlines elicit less engagement [27]. However, readers are more likely to click on question-based clickbait headlines than traditional headlines with no questions [28], even though other research shows that question-based clickbait headlines reduce a reader’s comprehension of the accompanying stories [29].

Emotionally charged words also affect readers. For instance, climate change denial clickbait headlines containing words that denote negative emotion, like anger, sadness, and disgust, elicit more engagement via commenting, liking, and sharing than headlines without such words [30]. On the other hand, such engagement is higher with climate change-supporting clickbait stories using words expressing anger. Likewise, readers are more likely to click on clickbait headlines that contain words that denote negative sentiment than traditional headlines without such words [28]. This is unsurprising given that clickbait headlines containing questions and emotional words such as “shocking” create arousal and curiosity among readers [26]. Such clickbait headlines have proven to be uniquely effective in arousing attention when addressing polarizing issues such as immigration, minority rights, and political extremism [31].

### The role of news headlines

A news headline serves two main purposes. In its *semantic* function, it summarizes and introduces the main topics of the news story it heralds [32,33]. In its *pragmatic* function, it captures the reader’s attention and entices them to read the story [34,35]. To do this, a headline must align the story’s relevance and context to the reader’s knowledge, beliefs, and expectations, and do so in a manner requiring minimal cognitive effort [36]. This is important because only 40% of news readers delve beyond the headline [7]. Additionally, online news readers are more likely to click on short, simple headlines using common words rather than longer headlines using complex linguistic styles [9].

Given that the headline is the first and probably the only part of a news story that the typical news reader sees and interacts with [7], headlines exemplify the proven adage that first impressions matter [37]. These first impressions are consequential. For instance, a misleading headline accompanying an opinion article may affect the reader’s judgment about the story’s subject even when that headline is incongruent with the gist of the article [38]. This means that even with an accurate headline, news consumers still need to read the entire story to fully comprehend the message [29]. Like with clickbait, valence also makes a difference. Here, the valence and context with which a headline is written also matter. Headlines written with negative valence words such as “suicide,” “cruelty,” “terror,” and “abuse” elicit more comments from readers than those with positive or neutral valence words [39]. Likewise, headlines written with alarm words such as “death,” “disaster,” and “catastrophe” improve clicking. Meanwhile, headlines with prosocial words such as “donation,” “cooperation,” and “touched” improve engagement, such as likes [40].

### News literacy and personal locus

News literacy refers to a person’s knowledge of and experience with the dynamics of news production and news consumption while considering the societal effects and that person’s control over these matters [41–43]. Even though the term was initially coined in 2008 to describe skills in detecting fact from fiction in online news [44], news literacy is part of the larger framework of media literacy, which broadly encompasses competencies in evaluating and producing media content [45]. Media literacy is the brainchild of the media literacy movement, a term that was codified by the movement’s proponents at the 1992 National Leadership Conference on Media Literacy in Queenstown, Maryland [45]. The above-mentioned personal control aspect of news literacy derives from personal locus, which is a key component in Potter’s 2004 reconceptualization of media literacy [12]. Here, Potter defines personal locus as a person’s control over their exposure to the media and their mindfulness of the resultant effects. Personal locus improves with a person’s drive for information, their ability to distinguish signal from noise in media messages, having a high tolerance for ambiguity, and being a reflective rather than an intuitive thinker [12].

Even though personal locus is a component of media literacy, research shows that it applies to news literacy. In Potter’s conceptualization of personal locus, a media literate person must first be an active information seeker [12]. Second, they must correctly differentiate news content from other content in the information-laden field of chaos they encounter when seeking news. Third, they must not avoid ambiguous or unexpected news, and fourth, they must perform all these activities with reflective rather than intuitive thinking. Regarding information-seeking, research shows that actively seeking news and having an interest in the news improve news literacy [11]. Also, news interest and news consumption improve the ability to determine the accuracy of news [46]. This may explain why news literacy training improves news literacy, fake news detection, awareness of one’s susceptibility to fake news, knowledge about news framing, and the logical evaluation of news content [47,48]. Such training is even more effective if deployed alongside inoculative strategies like forewarning, prebunking, and the preemptive refutation of related falsehoods and correcting inaccuracies [49–51].

News literacy may even explain why people with knowledge of proper news practices can identify related features when comparing well and poorly edited news stories. This explains why news-literate people are more likely to rate stories that carry real rather than generic photos and those with traditional rather than clickbait headlines as better written, more professional, better organized, and they also perceive them as more credible [52]. Also, perceived knowledge quality, or how people perceive headlines and news stories to be accurate and reliable, improves the likelihood of them reading the accompanying stories and sharing them with others [26]. These findings are similar to those reporting that people with an understanding of how headlines work are more likely to identify the sensational and deceptive characteristics of clickbait headlines and heed related warnings than those with less such knowledge [23]. Given the discussion above, I predict that news literacy and the drive for information will improve detection and reduce engagement with clickbait.

**H1:** News literacy is a) positively associated with clickbait detection and b) inversely associated with engagement with clickbait.

**H2:** The drive for information is a) positively associated with clickbait detection and b) inversely associated with engagement with clickbait.

Differentiating news content from other content is similar to that aspect of news literacy about differentiating between signal and noise. In defining news as “any accurate information that facilitates decision-making on both personal and social issues, thus enabling people to more effectively engage with society” [53] (p. 3), Vraga et al. emphasize the need to distinguish between signal and noise. Here, they point out the role of news literacy in distinguishing between news and the plethora of other information, both accurate and misleading, that people receive from a variety of sources [53]. Tully et al. emphasize the same in their reconceptualization of news literacy, where they propose five knowledge categories needed to attain news literacy competence [42]. These are context, creation, content, circulation, and consumption. In the content category, they emphasize the importance of distinguishing news content from other content, like opinions and advertisements. It is important, therefore, that news-literate people distinguish between editorial content and advertising content [10]. Additionally, research shows a positive association between news literacy and the discernment of fake news content [54,55]. Therefore, hypothesis 3 predicts the same regarding the effect of signal detection on detection and engagement.

**H3:** The ability to distinguish between signal and noise is a) positively associated with clickbait detection and b) inversely associated with engagement with clickbait.

Research shows that reflective thinking improves news literacy. In his seminal discourse on the science of thought, Dewey describes reflection as a chain of ideas ordered consecutively, not by chance, but by interconnectedness and mutual support [56]. Here, an idea grows from a previous one, and in turn, it supports the next idea in a successive chain or thread. Cognitive reflection, a form of reflective analytical thinking, improves the ability to distinguish between true and false headlines on social media [57]. Also, cognitive reflection, alongside digital media literacy, improves the detection of fake news headlines [58]. Additionally, people are more likely to correct their initial mistake of having intuitively misidentified false headlines as true after reflecting and deliberating upon their initial decision [59]. Other research supports the findings of the effect of reflective and analytical thinking on news accuracy discernment [60]. Therefore, hypothesis 4 predicts that reflective thinking will improve detection and reduce engagement with clickbait.

**H4:** Reflective thinking is a) positively associated with clickbait detection and b) inversely associated with engagement with clickbait.

The tolerance for ambiguity (TFA) is one’s ability to deal with situations of little clarity and/or the lack of information [61]. Ambiguity occurs when the same information suggests different meanings or when there is insufficient information to determine among different meanings [62,63]. While people who are intolerant of ambiguity see such situations as threatening, people with a high TFA welcome these situations [61]. Rather than ignoring unexpected information as people with low TFA do, highly tolerant people process this information further to improve comprehension, even if it contradicts their expectations [12]. These skills are important when evaluating fake news and other misinformation, where associated correction efforts lag behind the spread of the false content [64]. Indeed, research shows that among news users who are cognizant of their susceptibility to fake news, their perceptions of ambiguity about politics and the news media improve their information-seeking endeavors for verification purposes [13]. This is similar to the awareness a person has about the effects of media use described in personal locus. Given the discussion above, hypothesis 5 predicts that TFA will improve detection and reduce engagement with clickbait. Similarly, hypothesis 6 predicts that clickbait training will improve clickbait detection and reduce engagement with clickbait.

**H5:** The tolerance for ambiguity is a) positively associated with clickbait detection and b) inversely associated with engagement with clickbait.

**H6:** Clickbait detection training is a) positively associated with clickbait detection and b) inversely associated with engagement with clickbait

## Method

### Sampling and data collection

This study used both a survey and an experiment. The survey was used to test for the effect of news literacy, signal detection, reflective thinking, the tolerance for ambiguity, and the drive for information on clickbait detection and engagement with clickbait. The experiment tested the effect of clickbait detection training on detection and engagement. The experiment was a single-factor between-subjects design. The single factor had two levels: clickbait training vs. placebo training. I discuss the experiment details below. A nationally representative sample (N = 858) of U.S. social media users was used, and it was randomly drawn from a Qualtrics panel. Like Amazon Mechanical Turk, Qualtrics panels are pools of vetted research subjects that scholars may sample from for a fee [65], and they are widely used and validated [66,67]. A screening question was used to exclude those who reported rarely or never using social media. The sample had a 100% survey completion rate, and those who failed the attention check questions were eliminated from the sample. G*Power analysis, as advised by Faul, Erdfelder, Buchner, and Lang [68] and Faul, Erdfelder, Lang, and Buchner [69], showed that the sample was adequately powered. The G*Power analysis indicated that sample sizes of 51 for the test subjects and control group, respectively, were suitable for comparative analysis (.80 power, alpha = .05, two-tailed).

To attain representativeness, the sample was designed to reflect the U.S. Census regarding age, gender, race/ethnicity, and geographical region. The sample demographics were: *Age* (41.89, (s.d. = 15.82), *gender* (female = 51.7%, male = 48.1%, non-binary or other = .2%). *Race/ethnicity* (non-Hispanic white = 61.4%, non-Hispanic Black = 10%, Hispanic = 17.3%, Asian = 4%, Native American or American Indian. = .1%, American Indian or Alaska Native = 2.6%, other/mixed race = 4.5%). *Region* (East = 19.5%, Midwest = 22.8%, West = 22.4%, and South = 35.4%.). *Political affiliation* (lean Liberal = 31.9%, lean Independent = 28.7%, lean Conservative = 30.9%), other = 8.5%). Data were collected between February 26 and March 14, 2025. Because this study randomly assigned subjects to either the test group or control group, a comparison of the two groups’ demographics was done, and it showed no significant differences regarding age, gender, ideology, or income. Additionally, no differences emerged between the two groups regarding the survey-based variables. See supplemental Table S1 in S1 File for details.

### Ethics statement

The study was approved by the University of Texas at Rio Grande Valley Institutional Review Board before data collection (IRB-25–0007). Written consent was obtained from the subjects prior to participating in the study.

### Procedure and stimuli for the experiment

#### Procedure.

After answering the demographic questions, all participants performed the pre-test, where they randomly viewed ten headlines that were a mix of clickbait and non-clickbait headlines. See below for details about the headlines and the stimuli. Also, see the appendix for the pre- and post-test headlines and stimuli. For each headline, subjects first indicated whether it was clickbait or not. They then answered three clickbait engagement questions for each headline. I discuss the engagement variable under the variables measurement section. Subjects had 35 seconds to evaluate and respond to each headline. After responding to the headlines, subjects answered the rest of the survey, after which they were randomly assigned to their respective group. The test subjects were directed to a written tutorial on how to identify clickbait headlines. The control group subjects were directed to a placebo training, which was a written tutorial about news leads. While all subjects viewed the same headlines in the post-test, these headlines were different from those they had viewed in the pre-test. Click HERE to view the instrument and survey questionnaire.

#### Headline selection.

Béna et al. advise against using existing headlines because subjects may have read them before a study [22]. Therefore, this study took two measures to address this issue. The clickbait headlines were derived from Béna et al.’s study of clickbait. In their study, they created their own headlines following suggestions and procedures in previous studies [70,71] after which they tested the headlines with a pilot study sample. They then selected the headlines that the pilot test subjects deemed most likely to be clickbait, which they later used in the main experiment. In the current study, I used the verified clickbait headlines from the Béna et al. study. I then randomly selected non-clickbait headlines from the *Associated Press (AP)* and *The New York Times* archives. To reduce the chances of recall, the non-clickbait headlines were at least ten years old and were published between 2013 and 2014. Following Béna et al.’s methods, I tested all headlines in a small non-random sample (N = 22). Here, subjects correctly identified 62% of the non-clickbait headlines from the *AP* and *New York Times* archives and 60% of the clickbait headlines derived from the Béna et al. study.

### Stimuli

The test group subjects viewed a written clickbait identification tutorial developed from guidelines from various clickbait studies discussed earlier. To prevent priming the subjects, the tutorial did not mention the word clickbait; rather, it was titled as a description of detecting deceitful headlines. The tutorial first informed the subjects about the purpose of headlines, after which they read about the four types of clickbait techniques with respective examples for each technique, as shown in the excerpt below:

The headline may contain forward-references. These are suggestive words that raise your curiosity by referring to aspects of the story in advance, but with no further details.

Example: He loves Beatles, menthol cigs..and longs for muscles like Van Damme – ***The forward reference is the word “he.” Unless the person’s identity is obvious, a good headline should name them.***

Subjects then took a short, multiple-choice practice quiz based on this tutorial. I elaborate on this quiz in the manipulation check section. Subjects then viewed the quiz answers before proceeding to the post-test portion of the experiment. The control group viewed a different tutorial on news leads. They first read a definition of a news lead, after which they viewed examples of the who, what, when, and where of a news lead. See excerpt below. They then did a short, multiple-choice quiz where they were asked t to identify the who and when of a sample lead. They also viewed their answers before proceeding to the post-test section of the experiment.

BOSTON — Jaylen Brown scored a season-high 44 points, including six 3-pointers, and the Boston Celtics snapped a two-game skid with a 142-105 rout over the Indiana Pacers on Friday night.

Who: Jaylen Brown.

What: Scored a season-high 44 points.

When: Friday night.

Where: In Boston.

### Variable measurement

The four dependent variables were clickbait *detection* and *engagement* with clickbait via clicking the headlines, reading the accompanying story, and sharing it. The six independent variables were news literacy, the four parameters of personal locus (the ability to distinguish between signal and noise, reflective thinking, the tolerance for ambiguity, and the drive for information), and clickbait detection training.

#### Detection.

For detection, subjects indicated whether a headline was clickbait or non-clickbait. Two detection scores (pre- and post-test, respectively) were computed based on the number of correctly identified headlines, whether they were clickbait or not, as done in other news veracity discernment studies that based identification scores on both real and fake headlines [72]. Therefore, the maximum detection score was 10 points for the pre- and post-test, respectively.

#### Engagement.

For each headline, subjects also indicated how likely they were to click on the headline, read the story that accompanied the headline, and share it. Responses were captured using a 1–5 not at all likely/very likely scale. A composite engagement score was then computed from the average of responses to the three questions (Cronbach’s alpha = .95). This is similar to procedures used in previous studies [26]. The maximum engagement score was 5 points.

#### News literacy.

News literacy was measured using Ashley et al.’s modified scale [43], which is based on previous scales [10,41,73] and also considers the aforementioned five knowledge categories needed to attain news literacy competence (context, creation, content, circulation, and consumption) as proposed by Tully et al. [42]. Additionally, their scale considers the dynamics of digital media and social media. Therefore, subjects randomly answered 15 questions about the five categories. The response options were either dichotomous (true/false) or multiple-choice, depending on the question. A correct answer was worth one point for a maximum news literacy score of 15 points.

#### Distinguishing signal from noise.

This variable measured the ability to distinguish news content from advertising content, as done in previous studies [10,74]. Therefore, subjects randomly viewed a combination of six news and four advertorial headlines. Advertorials were used instead of traditional advertisements because they deceptively mimic editorial content while promoting products, thus requiring a higher ability to distinguish signal from noise [75,76]. The advertorials were taken from the product promotion and review sections of various news sites such as *NBC Select, HuffPost Shopping*, and *The New York Times Wirecutter.* The news headlines were taken from the news sections of these news sites. For each headline, subjects were asked to determine how much advertising messaging (for products and services) each headline contained. Responses were captured on a 1–5 scale, where 1 = no advertising at all and 5 = a lot of advertising. Reverse coding was used for the non-advertorial headlines. A composite signal detection score was then calculated from the average of responses for a maximum of 10 signal detection points (Cronbach’s alpha = .85).

#### The tolerance for ambiguity.

This study used Herman et al.’s TFA scale [77], which improves on Budner’s widely used scale [61]. The Herman et al. scale addresses two main shortcomings of the previous scale. First, it reports higher internal reliability. Budner’s 16-item scale reports a reliability of *α* = .57, while the Herman et al. 12-item scale reports a reliability of *α* = .73. Second, the modified scale is more suitable for measuring ambiguity across broader cultural contexts than the previous scale. Hence, subjects responded to twelve statements based on a 1–5 scale, where 1 = strongly disagree and 5 = strongly agree. Depending on the direction a statement suggested, several were reverse-coded. A composite TFA score was then computed from the average of the responses (Cronbach’s alpha = .83). An example of a TFA statement is: *I like to surround myself with things that are familiar to me* (reverse-coded).

#### The drive for information.

As mentioned, this aspect of personal locus refers to the motivation to seek information [12]. Because research shows that seeking news and having an interest in the news both improve news literacy [11,41,46] subjects were queried about both issues using questions derived from the studies cited above. Subjects first indicated how often they accessed news (regional, national, international, etc.) via any platform, such as newspaper, TV, radio, and online, on rarely/never to several times a day options measured on a 1–5 scale. They then indicated their general interest in the news based on not at all interested to very interested options, also on a 1–5 scale. A composite drive for information score was computed from the average of responses from the two questions (Cronbach’s alpha = .77).

#### Reflective thinking.

Most studies use the cognitive reflective test (CRT) to measure reflective thinking. The CRT requires subjects to solve simple arithmetic problems that are prone to intuitive answers [78]. One question asks, “*A bat and a ball cost $1.10. The bat costs $1.00 more than the ball. How much does the ball cost?* (p. 65). The correct answer is 5 cents, and the intuitive answer is 10 cents [78]. However, even though it tests for thinking styles, the CRT also tests for numeracy and the ability to solve mathematical problems [79]. Therefore, this study used the CRT-2, a newly developed non-numerical test by Thomson & Oppenheimer, which has proven validity and is moderately correlated with the older version [80]. Here, subjects answered five non-numeric questions suggested by Thomson & Oppenheimer, such as, *“if you’re running a race and you pass the person in second place, what place are you in?”* (p. 67). The correct answer is second place, and the intuitive answer is first place. Subjects randomly answered five such questions for a maximum reflective thinking score of five points. The author added a fifth question to the original four-question scale: *Which is heavier? A 50-pound bag of wet sand or a 50-pound bag of dry sand.* Additionally, each question was timed at 25 seconds.

#### Manipulation check.

The in-study quiz that the test subjects in the main experiment took after exposure to the clickbait training stimuli acted as a manipulation check. After exposure to the clickbait training but before viewing the post-test headlines, the test group subjects answered two multiple-choice questions. The first question asked them to identify the forward-reference in a headline, and the second asked them to identify the emotionally charged word in a second headline. Subjects were shown the correct answers after completing the quiz. Overall, 80% of the subjects correctly identified the forward-reference, and 65% correctly identified the emotionally charged word.

#### Statistical analysis.

Statistical analysis included correlation, OLS regression, and ANOVA, and was conducted using SPSS 28. While the demographic variables were not required for analyzing the experiment-based data because the randomization was successful (see Supplemental Table S1 in S1 File), they were still used as control variables in the regression analysis for exploratory purposes.

## Results

Hypotheses 1–5 were tested based only on data collected in the pre-test. Hypothesis 1 predicted that news literacy would improve clickbait detection and reduce engagement with clickbait. Data supported this hypothesis. First, as Table 1 shows, news literacy was positively correlated with detection (*r* = .29, *p* < .001) and inversely correlated with engagement with (*r* = −.30, *p* < .001). Second, OLS regression results (shown in Tables 2 and 3) indicated that news literacy had main effects increasing detection (*β* = .22, *t* = 5.54, *p* < .001) and reducing engagement (*β* = −.15, *p* < .001). Hypo*t*hesis 2 was not supported. Not only was the drive for information not correlated with detection, but it was positively correlated with engagement (*r* = .41, *p* < .001. Additionally, it had a main effect on engagement (*β* = .34, *t* = 11.64, *p* < .001). When the individual engagement parameters are considered, the drive for informa*t*ion had effects on all three parameters: Clicking (*β* = .34, *p* < .001), reading (*β* = .34, *p* < .001), and sharing (*β* = .32, *p* < .001). See supplemental Tables S2–S4 in S1 File for details.

**Table 1:** Pre-test correlations among variables.

	1	2	3	4	5	6	Mean	S.D.
News literacy	--						5.73	2.81
Reflective thinking	.31**	--					1.62	1.32
Tolerance for ambiguity	.11**	.08*	--				2.88	0.40
Signal detection	.43**	.19**	.15**	--			3.19	0.60
Drive for Information	.12**	0.02	−.11**	−.13**	--		3.72	1.12
Detection	.29**	.19**	0.05	.25**	0.03	--	5.66	1.58
Engagement	−.30**	−.17**	−.15**	−.49**	.41**	−.29**	2.71	1.11

** *p* < .01.

**Table 2:** Pre-test OLS Regressions predicting clickbait detection.

	*β!* ^†^	S.E.	*t*	95% CI		*p*	VIF
				*LL*	*UL*		
Constant^***^		.56	5.83	2.16	4.35	.001	
News literacy^***^	.22	.02	5.54	.08	.17	.001	1.48
Signal detection^***^	.16	.10	4.33	.23	.61	.001	1.31
Reflective thinking^**^	.10	.04	2.91	.04	.20	.004	1.13
Tolerance for ambiguity^*^	.00	.13	.04	−.26	.27	.967	1.08
Drive for information	.06	.05	1.66	−.02	.19	.097	1.29
Age^*^	−.07	.00	−2.09	−.01	.00	.037	1.20
Income – low	.07	.14	1.72	−.03	.50	.085	1.55
Income – middle	.05	.13	1.40	−.08	.44	.163	1.34
Leans Independent	.01	.13	.27	−.22	.30	.784	1.40
Leans Conservative	.02	.13	.48	−.19	.32	.63	1.42
Leans other	.00	.20	−.08	−.42	.39	.94	1.26

R^2^ = .13; Adj. R^2^ = .11

Income – high and Leans Liberal were excluded due to collinearity.

† Standardized beta coefficients shown.

**Table 3:** Pre-test OLS regression predicting engagement with clickbait.

				95% CI			
	*b!* ^†^	S.E.	*t*	*LL*	*UL*	*p*	VIF
Constant		.32	16.53	4.87	6.18	.001	
News literacy	-.15	.01	-4.90	-.09	-.04	.001	1.48
Signal detection	-.33	.06	-11.21	-.75	-.53	.001	1.31
Reflective thinking	-.07	.02	-2.47	-.11	-.01	.014	1.13
Tolerance for ambiguity	-.06	.08	-2.31	-.34	-.03	.021	1.07
Drive for information	.34	.03	11.64	.30	.42	.001	1.29
Age	-.20	.00	-6.91	-.02	-.01	.001	1.20
Income – low	-.21	.08	-6.41	-.70	-.38	.001	1.56
Income – middle	-.13	.07	-4.39	-.52	-.21	.001	1.34
Leans Independent	-.01	.08	-.19	-.17	.15	.848	1.39
Leans Conservative	-.04	.07	-1.28	-.26	.05	.202	1.42
Leans other	-.04	.12	-1.29	-.40	.08	.198	1.26

*R*^*2*^ = ..44; *Adj. R*^*2*^ = .44

Income – high and Leans Liberal were excluded due to collinearity.

† Standardized beta coefficients shown.

Data supported H3. Signal detection was positively correlated with clickbait detection (*r* = .25, *p* < .01) and inversely correlated with engagement with clickbait (*r* = −.49, *p* < .01). OLS regression supported these findings. Distinguishing between signal and noise improved clickbait detection (*β* = .16, *p* < .001) and reduced engagement (*β* = −.33, *p* < .001). Regarding H4, data showed significant results for both correlations and regression, thus supporting this hypothesis. Here, reflective thinking was positively correlated with detection *(r* = .19, *p* < .01) and inversely correlated with engagement (*r* = −.17, *p* < .01). OLS regression showed a moderate main effect on improving detection (*β* = 10, *p* < .01) and marginal but statistically significant effects on reducing engagement (*β* = −.07, *p* < .01). Hypothesis 5 was partially supported. While the TFA was not correlated with detection, it was inversely correlated with engagement (*r* = −.15, *p* < .01). Also, while it did not have a main effect on detection, the TFA marginally impacted engagement (*β* = −.06, *p* < .05).

Hypothesis 6 predicted that clickbait training would improve clickbait detection and reduce the likelihood of engagement via clicking, reading, and sharing. Data from the experiment partially supported these predictions. One-way ANOVA was used to analyze the effect of the intervention (clickbait training) on detection and engagement, respectively. To account for changes over time, a difference score was calculated by subtracting the post-test scores from the pre-test scores for clickbait detection and engagement. The difference score was then used as the dependent variable in the ANOVA analysis, as done in previous studies [81]. For instance, if a subject scored 2 points in their pre-test detection and 5 in the post-test, their detection score was 3. The data returned no significant differences between the test and control groups regarding detection. Therefore, H6a was not supported even though the test group (pre-test = 5.64, post-test = 5.90) showed more improvement than the control group (pre-test = 5.68, post-test = 5.84). Additionally, statistical significance emerged when the detection of only clickbait headlines was considered. However, this study calculated detection based on correctly identifying both clickbait and non-clickbait headlines.

On the other hand, ANOVA analysis showed significant differences between the test and control groups regarding engagement, thus supporting H6b. Here, the test group subjects were less likely to engage with clickbait after exposure to clickbait training (pre-test = 2.99, post-test = 2.85; F = 5.14; *p* < .05) than the control group, who were not exposed to this training (pre-test = 2.83, post-test = 2.79). Separate analysis on the three endearment parameters showed that the significant differences occurred with reading and sharing clickbait. See supplemental Table S7 in S1 File. I contextualize these H6a and H6b findings in the discussion section.

## Discussion

This study deployed both a survey and an experiment to examine how news literacy and clickbait training affect clickbait detection and engagement with clickbait via clicking, reading, and sharing. Research shows that news-literate readers are better at detecting clickbait and other false news content, and the results reported here support these findings. Not only was news literacy positively correlated with clickbait detection (*r* = .29), but the regression analysis indicated a direct effect on detection (*β = .19*). Additionally, news literacy was also inversely correlated with engagement (*r* = −.29), and regression results also showed direct effects (*β = −15*). Supplemental Tables S2–S4 in S1 File show similar associations when considering the individual engagement parameters. These results reflect other research on how news literacy affects news veracity discernment [26]. It is noteworthy that news literacy had these effects despite the low performance on news literacy for the entire sample. On average, the sample scored 5.72 (s.d. = 2.81) vis-à-vis the maximum possible score of 15 points for news literacy.

Another unique finding was the effect of personal locus. As discussed, personal locus is a facet of media literacy, but this study reveals that it applies to news literacy as well. This is a seldom-researched area. The four personal locus variables examined here showed unique associations with both news literacy and engagement with clickbait. As Table 1 shows, all four personal locus variables (reflective thinking, the tolerance for ambiguity, distinguishing between signal and noise, and the drive for information) were positively correlated with news literacy. Likewise, reflective thinking and signal detection were positively correlated with clickbait detection, while all but one of the personal locus variables (the drive for information) were inversely correlated with engagement with clickbait. Additionally, OLS regression showed that not only did signal detection improve clickbait detection (*β = .19*), but it had a substantive effect on reducing engagement with clickbait (*β = −33*).

This suggests that news-literate people are more analytical than intuitive when reading news, suggesting a more in-depth reading of the news. The fact that signal detection was also positively associated with clickbait detection gives insight into how these news-literate people read the news; that is, they are selective readers who look for cues and signals within the news content. The positive association between the TFA and news literacy and clickbait detection, respectively, also suggests that apart from savvily looking for signals when reading the news, news-literate people are not deterred by complex and hard-to-comprehend news. They can handle complexity and ambiguity in news content. This is important given that the coverage of hard news on complex topics like politics, finance, and crime, etc. has declined [82] at the expense of soft news on topics like sports and entertainment. This is even more remarkable in the context of the superficial reading of news, given that only 40% of people read beyond the news headline [7].

However, the drive for information performed contrary to both extant research and to this study’s predictions. Research indicates that the drive for information improves both news literacy and the detection of false information [11,41,43]. However, while this variable was positively correlated with news literacy, it was not associated with clickbait detection and was highly correlated with engagement with clickbait. Also, OLS regression showed that it had substantive main effects on increased engagement with clickbait. A possible explanation could be the nature of the sample, which consisted only of social media users. Research shows that using social media as a source for news and information increases one’s susceptibility to misinformation and the likelihood of sharing misinformation [83,84]. Therefore, searching for and sourcing news and information from social media may have increased the likelihood of engaging with clickbait even as news literacy improved, thus raising a paradox. As mentioned earlier, social media users often share news links without reading or clicking on them [8]. This, too, may explain why the drive for information was positively associated with engagement with clickbait. Subjects who seek news and information from social media may also be more likely and eager to share whatever links they found interesting, regardless of whether the links were clickbait or not. Additionally, we must consider that sharing content is a fundamental feature of social media interaction, with perceived benefits such as social presence and social conversations [85].

This observation segues well into the experimental results that showed that clickbait training did not affect clickbait detection (H6a), but had a significant effect in reducing engagement with clickbait (H6b). The H6a null results contradict research on the effect of news literacy training on news veracity discernment and the detection of misinformation [47,48]. The same explanations about the null results for the drive for information variable may apply here. As mentioned, social media use increases the susceptibility to misinformation, and a sample composed solely of social media users may reflect a similar susceptibility to clickbait. Another explanation is that because this was a cross-sectional study, the one-time clickbait training session was not enough to improve detection. Some of the news literacy training studies cited earlier in this study were longitudinal, with some lasting weeks and months [47,48].

Because this study calculated detection based on the collective identification of both clickbait and non-clickbait headlines, I ran further analysis based on the detection of clickbait headlines only. There was statistical significance in detection when only clickbait headlines were considered, with the test subjects showing more improvement (pre-test = 3.10; post-test = 3.36, F = 4.54; *p* < .05) than the control group subjects (pre-test = 3.22; post-test = 3.30). There was no statistical significance when only non-clickbait headlines were considered. Additionally, paired-samples *t-*test results run for the test and control group separately showed a bigger effect of the clickbait training on detection among the test group subjects, regarding the level of significance and the effect size coefficients. See supplemental Tables S5 and S6 in S1 File.

The H6a null findings show that the H6b significant findings are therefore unique, given that the one-time clickbait detection training conducted in a cross-sectional study reduced engagement with clickbait. This is noteworthy because, for one, research shows that 75% of news links shared on social media are shared without the sender clicking or reading them. The fact that clickbait training reduced engagement in a social media-only sample is unique. It is noteworthy, considering that further ANOVA analysis on the individual engagement parameters showed that the training significantly reduced reading and sharing more among the test group than among the control group. See supplemental Table S8 in S1 File. This suggests that even though the training did not improve detection, it reduced the reading and sharing of clickbait. In a broader perspective, this is important because social media use is associated with habitual sharing of news, which then leads to misinformation susceptibility [84]. Lastly, the deterrent effect of the training towards reading and sharing clickbait reflects anti-misinformation inoculation strategies that I discuss below in the implications.

While this study did not predict the effects of any demographic variables, age, income, and ideology were considered as control variables during data analysis. Except for age, all categorical demographic variables were dummy coded. Important findings emerged. First, none of the demographic variables affected detection. Even though age showed statistical significance, this was marginal. However, age and income had discernible inverse effects on engagement. This means that older subjects were less likely to click, read, and share clickbait, as were subjects in higher income categories. These findings confirm extant research showing an inverse association between income and news veracity discernment and engagement with fake news [86]. They also reflect the mixed findings in current research showing that while older adults are better at news veracity discernment, they also share more misinformation than younger social media users [87–89]. Additionally, ideology did not affect either detection or engagement. This contradicts research showing an association between Conservative ideology and misinformation susceptibility [90].

### Implications and limitations

The unique results discussed above have both theoretical and practical implications. Theoretically, the study adds to news literacy research and expands the avenues of scholarly inquiry into related fields like psychology and misinformation susceptibility research. It does this by applying measures from other fields of study or even from those within, such as with personal locus. For instance, reflective thinking was measured using a psychology-related measure, the cognitive reflection test. The same applies to TFA, where the study used a modified version of Budner’s personality and psychology measure [61]. This is in addition to using an advertising measure for signal detection. Because some clickbait uses deception, this study also contributes to misinformation susceptibility scholarship. Specifically, the clickbait training stimuli used here echo anti-misinformation training strategies like psychological inoculation, which, as I discuss below, is also similar to clickbait detection training regarding practicality. Therefore, this provides another avenue for comparative inquiry that could examine the association between clickbait training and inoculation training. Practically, the study informs stakeholders in the news literacy movement as well as those involved in combating misinformation. Here, stakeholders may design training materials such that they improve aspects of personal locus like TFA, signal detection, and reflective thinking. Such training may also include clickbait detection training, given that this successfully reduced engagement with clickbait. It would not be a stretch to suggest that clickbait training should be incorporated into inoculation training meant to combat misinformation. As mentioned, research shows inoculation strategies such as prebunking and preemptively refuting falsehoods reduce the susceptibility to misinformation [49,50]. The H6a results also suggest that such training needs to be done over time across multiple sessions rather than in a single session, as done in this study.

Lastly, this study comes with various limitations. Even though the study used an experiment to examine main effects, the sample was exclusively composed of social media users. Therefore, the results are generalizable only to this group. Additionally, the stimuli were presented as plain text headlines, unlike real-life clickbait that is accompanied by equally captivating thumbnail images and other visuals [91]. It is also important to point out that the selection for both the clickbait and non-clickbait headlines did not consider news type (economic, crime, sports, science news, etc.) or content valence regarding controversial versus neutral headlines. Also, the four personal locus variables were measured using self-reported responses that may be susceptible to validity issues caused by self-report bias [92,93]. We must also consider that for the manipulation check, subjects only viewed two headlines, and they were asked to identify only two features: a clickbait headline, a forward-reference, and the emotionally charged word. Any attempts to replicate this study should use a more diverse and comprehensive approach. Demand effects and question order effects may also have occurred because subjects first viewed the headlines, after which they answered the survey questions about news literacy, reflective thinking, and signal detection, and then viewed the post-test headlines. However, this concern is mitigated by the fact that the survey questions (and each headline) were designed in blocks, and the blocks were presented randomly to the subjects as suggested by Rasinski et al. [94]. Lastly, and as mentioned, this was a cross-sectional study that used stimuli similar to those used in longitudinal multi-test studies.

## Supporting information

S1 File Supporting Information Files. (DOCX)
