# Supplementary material for: SHOCKING! College professor reveals hidden secrets of news headlines. Can you guess? Examining the efficacy of clickbait detection training and news literacy
Source: PLoS One. 2026 Nov 5;21(11):e0359401. doi: 10.1371/journal.pone.0359401 (PMC13739131; doi:10.1371/journal.pone.0359401)
Supplement: Supplementary file 1 [file pone.0359401.s003.pdf]

The purpose of the review is to provide the editors with an expert opinion regarding the validity and quality of the manuscript under consideration. The review should also supply authors with explicit feedback on how to improve their papers so that they will be acceptable for publication in *PLOS One*. As you write, consider the following points:

- What are the main claims of the paper and how significant are they for the discipline?
    - The study examines the effects of news literacy and a clickbait training on clickbait detection and clickbait behavior. The author found that readers who are news literate are less likely to engage in clickbait behavior. Additionally, a brief training seemed to improve clickbait detection and decrease clicking. It seems that the American public (based on the sample used) is not very news literate. The subject of this paper is certainly relevant in today's climate of polarization, where accusations of fake news are being used widely and wildly to discredit and silence.
- Are the claims properly placed in the context of the previous literature? Have the authors treated the literature fairly?
    - The study is embedded in the context of previous literature well.
- Do the data and analyses fully support the claims? If not, what other evidence is required?
    - The data and analyses show significant relationships between literacy and clickbait detection. The brief training showed improvement in detecting clickbait. The author does draw causation with data that only shows correlation (discussion, line 7 of page 25), which needs to be revised. The statement "news literacy…directly improved detection" is not correct based on the analyses. Correlation does not equal causation…
    -
- Are details of the methodology sufficient to allow the experiments to be reproduced?
    - Yes. As a minor point, Table 1 is not formatted correctly; the M and SD columns belong on the right-hand side.
- Is any software created by the authors freely available?
    - N/A
- Is the manuscript well organized and written clearly enough to be accessible to non-specialists?
    - Yes. The paper is written very clearly.
- Is it your opinion that this manuscript contains an NIH-defined experiment of Dual Use concern?

Although confidential comments to the editors are respected, any remarks that might help to strengthen the paper should be directed to the authors themselves.
