# Supplementary material for: SHOCKING! College professor reveals hidden secrets of news headlines. Can you guess? Examining the efficacy of clickbait detection training and news literacy
Source: PLoS One. 2026 Nov 5;21(11):e0359401. doi: 10.1371/journal.pone.0359401 (PMC13739131; doi:10.1371/journal.pone.0359401)
Supplement: Supplementary file 2 [file pone.0359401.s001.docx]

**S1 Appendix – Instrument for the Experiment**

**Test Group Stimuli (Clickbait Training Tutorial)**

Below is a description of how to detect deceitful news headlines.

News headlines serve two main purposes. First, they summarize a news story and second, they attract attention and encourage people to read the story. A good news headline must do both. Even though good headlines are meant to encourage people to read a story, the headline should not use deception to do that.

Deceptive headlines use certain tactics to make you read a story:

The headline may be exaggerated and/or hyperbolic.

*Example: This Amazing Kid Died. What He Left Behind is Wondtacular.*

The headline may contain unnecessary emotionally charged words.

*Example: You Will Be Amazed to Learn About Nelson Mandela's Unknown Boxing Career!*

The headline may ask a question rather than simply summarizing the story.

*Example: Do you know hyperesthesia? A little-known disorder of intolerance to pressure, pain and touch.*

The headline may contain forward-references. These are suggestive words that raise your curiosity by referring to aspects of the story in advance but with no further details.

*Example: He loves Beatles, menthol cigs..and longs for muscles like Van Damme* **– The forward reference is the word “he.” Unless the person’s identity is obvious, a good headline should name them.**

The headline may deceptively pitch or promote a product or service:

*Example: Why You’ve Never Heard of This Top Travel Destination.*

**Clickbait Detection Quiz (Answers Shown in Highlight)**

***The News Lead***

Please identify the forward-reference in the following headline:

This Sociologist Thought He Was Doing the Right Thing By Revealing That Most Work Accidents Happen On Friday. It Ruined the Lives Of Many Entrepreneurs!

- Many entrepreneurs
- This sociologist
- The right thing
- Happen on Friday.

Please identify the emotionally charged word in the following headline:

Scientists Make An Amazing Discovery! People With Blood Type Ab+ Can Donate To All People With Type A and Type B.

- Scientists
- All people
- Blood
- Amazing.

**Control Group Stimuli (Placebo News Lead Training Tutorial)**

The news lead in is the first paragraph of a news story and it tells the reader about the most important aspects of the story. These are the who, what, when, and where of the story.

Who: Refers to the person the story is about.

What: Refers to the action the person did.

When: Refers to when the action occurred.

Where: Refers to where the action happened.

Example

BOSTON — Jaylen Brown scored a season-high 44 points, including six 3-pointers, and the Boston Celtics snapped a two-game skid with a 142-105 rout over the Indiana Pacers on Friday night.

Who: Jaylen Brown.

What: Scored a season-high 44 points.

When: Friday night.

Where: In Boston.

**News Lead Quiz (Answers Shown in Highlight)**

Now that you know the structure of the lead, please identify the who and when in the lead shown below.

SAN DIEGO (AP) — Kyle McCord threw for 453 yards and five touchdowns to break Deshaun Watson's Atlantic Coast Conference season passing record and lead No. 22 Syracuse to a 52-35 victory over depleted-yet-scrappy Washington State in the Holiday Bowl on Friday night.

Identify the "who"

- Deshaun Watson
- Kyle McCord
- Holiday Bow
- Friday night

Identify the "when"

- Deshaun Watson
- Kyle McCord
- Holiday Bow
- Friday night

**Headlines**

- *For each headline, please indicate whether it is a clickbait or non-clickbait headline.*
- *How likely are you to click on this headline?*
- *How likely are you to read the story that comes with this headline?*
- *How likely are you to share this story?*

**NOTE: Subjects viewed one headline at a time and in random order**

***Pre-test Clickbait Headlines***

1. You Will Be Amazed to Learn About This Famous President’s Unknown Boxing Career – *clickbait.*
2. ALS Drug Wins FDA Approval Despite Questionable Data – *non-clickbait.*
3. 51 Migrants Die After Trailer Abandoned in San Antonio Heat – *non-clickbait.*
4. Do You Know Meniere's Disease? A Little-Known Disorder That Causes Imbalance – *clickbait.*
5. School Bus Driver Charged with Drunken Driving on Field Trip – *non-clickbait.*
6. Most Americans Could File Their Taxes for Free, But Don't – *non-clickbait*
7. Incredible Discovery! Massive Ocean Found 700 Kilometers Beneath Earth’s Surface – *clickbait.*
8. The World's Favorite Destination In 2024. The Charms of This Country Will Surprise You – *clickbait.*
9. Did You Know Alpacas Can Be Allergic to Humans,? If You did, You're Probably Smarter Than Average – *clickbait.*
10. MGM Resorts Sells Land That Was Site Of Las Vegas Massacre – *non-clickbait..*

***Post-test Clickbait Headlines***

1. Nevada Governor Orders Corrections Secretary to Resign After Botched Inmate Escape That Went Unreported For 4 Days – *non- clickbait.*
2. New Animal Record: This Creature Just Reached 190 Years; It Is Officially the Oldest Ever Recorded – *clickbait.*
3. A Medieval Scientist Almost Burned to Death Trying to Prove That This Chemical Is Flammable – *clickbait.*
4. Russians Push Baseless Theory Blaming US For Burst Pipeline – *non- clickbait.*
5. Shocking Info: Was Andy Warhol Really Bisexual? – *clickbait.*
6. This TV Presenter Has Been Missing Since Announcing Jane Fonda's Non-Hodgkin Lymphoma – *clickbait.*
7. US Bankruptcy Court Approves $121M Clergy Abuse Settlement – *non- clickbait.*
8. This Person Was Imprisoned After Exposing the Government’s Role in The Decline of These Animals – *clickbait.*
9. Study Shows Your Dog's Personality May Have Little to Do with Its Breed – *non- clickbait.*
10. West Nile Virus Is Behind Bald Eagle Deaths in Utah – *non- clickbait.*

| **S1 Table**  *Comparisons of Demographics Between Test and Control Groups* | | | | |
| --- | --- | --- | --- | --- |
|  |  | **Test Group** | **Control Group** | ***p*-value** |
| Age |  | 42.11 | 41.68 | .70 |
| Gender |  |  |  | .25 |
|  | Male | 213 | 199 |  |
|  | Female | 216 | 227 |  |
|  | Non-binary | 0 | 2 |  |
| Ideology |  |  |  | .27 |
|  | Leans Independent | 133 | 113 |  |
|  | Leans Liberal | 125 | 148 |  |
|  | Leans Conservative | 132 | 133 |  |
|  | Other | 39 | 34 |  |
| Income |  |  |  | .98 |
|  | Less than $50,000 | 136 | 134 |  |
|  | Less than $50,000 | 118 | 117 |  |
|  | More than 100.001 | 175 | 177 |  |
| Test group N = 429  Control group N = 428  Age denotes t-test; gender, ideology, and income denote chi-square test | | | | |

| **S2 Table**  *Comparison of Survey-related Variables Between The Test and Control Groups* | | | | |
| --- | --- | --- | --- | --- |
|  |  | Mean | S.D. | *p* |
| News literacy | Test group | 5.72 | 2.77 | .99 |
|  | Control group | 5.73 | 2.86 |  |
| Reflective thinking | Test group | 1.59 | 1.31 | .56 |
|  | Control group | 1.64 | 1.33 |  |
| Tolerance for ambiguity | Test group | 2.88 | .40 | .76 |
|  | Control group | 2.89 | .39 |  |
| Signal detection | Test group | 3.18 | .63 | .88 |
|  | Control group | 3.19 | .58 |  |
| Drive for information | Test group | 3.74 | 1.07 | .54 |
|  | Control group | 3.70 | 1.17 |  |

| **S3 Table**  *OLS Regressions Predicting Clicking on Clickbait in the Pre-test..* | | | | | | | |
| --- | --- | --- | --- | --- | --- | --- | --- |
|  |  |  |  | **95% CI** | **95% CI** |  |  |
|  | ***β!***^†^ | **S.E.** | ***t*** | *LL* | *UL* | ***p*** | **VIF** |
| Constant |  | .35 | 15.35 | 4.71 | 6.09 | .001 |  |
| News literacy | -.10 | .01 | -3.15 | -.07 | -.02 | .002 | 1.48 |
| Signal detection | -.31 | .06 | -9.86 | -.72 | -.48 | .001 | 1.31 |
| Reflective thinking | -.06 | .03 | -1.90 | -.10 | .00 | .06 | 1.13 |
| Tolerance for ambiguity | -.07 | .08 | -2.50 | -.37 | -.04 | .013 | 1.08 |
| Drive for information | .33 | .03 | 1.90 | .29 | .42 | .001 | 1.29 |
| Age | -.19 | .00 | -6.30 | -.02 | -.01 | .001 | 1.20 |
| Income – low | -.21 | .09 | -6.15 | -.70 | -.36 | .001 | 1.55 |
| Income – middle | -.13 | .08 | -4.24 | -.51 | -.19 | .001 | 1.34 |
| Leans Independent | .00 | .08 | .01 | -.16 | .17 | .99 | 1.40 |
| Leans Conservative | -.05 | .08 | -1.43 | -.28 | .04 | .15 | 1.42 |
| Leans other | -.05 | .13 | -1.49 | -.44 | .06 | .14 | 1.26 |
| *R^2^ =* 38; *Adj. R^2^ =* .38  Income – high and Leans Liberal were excluded due to collinearity.  ^†^Standardized betas shown. | | | | | | | |

| **S4 Table**  *OLS Regressions Predicting Reading Clickbait Stories in the Pre-test..* | | | | | | | |
| --- | --- | --- | --- | --- | --- | --- | --- |
|  |  |  |  | **95% CI** | **95% CI** |  |  |
|  | ***β!***^†^ | **S.E.** | ***t*** | *LL* | *UL* | ***p*** | **VIF** |
| Constant |  | .34 | 15.70 | 4.67 | 6.01 | .001 |  |
| News literacy | -.11 | .01 | -3.53 | -0.07 | -0.02 | .001 | 1.48 |
| Signal detection | -.31 | .06 | -1.22 | -0.72 | -0.49 | .001 | 1.31 |
| Reflective thinking | -.07 | .03 | -2.41 | -0.11 | -0.01 | .02 | 1.13 |
| Tolerance for ambiguity | -.07 | .08 | -2.34 | -0.35 | -0.03 | .02 | 1.08 |
| Drive for information | .34 | .03 | 11.38 | 0.30 | 0.42 | .001 | 1.29 |
| Age | -.19 | .00 | -6.64 | -0.02 | -0.01 | .001 | 1.20 |
| Income – low | -.20 | .08 | -6.04 | -0.67 | -0.34 | .001 | 1.55 |
| Income – middle | -.14 | .08 | -4.59 | -0.53 | -0.21 | .001 | 1.34 |
| Leans Independent | .01 | .08 | .21 | -0.14 | 0.18 | .83 | 1.40 |
| Leans Conservative | -.03 | .08 | -.96 | -0.23 | 0.08 | .34 | 1.42 |
| Leans other | -.03 | .12 | -.93 | -0.36 | 0.13 | .35 | 1.26 |
| *R^2^* = ..40; *Adj. R^2^ =* .40  Income – high and Leans Liberal were excluded due to collinearity.  ^†^Standardized betas shown. | | | | | | | |

| **S5 Table**  *OLS Regressions Predicting Sharing Clickbait Stories in the Pre-test.* | | | | | | | |
| --- | --- | --- | --- | --- | --- | --- | --- |
|  |  |  |  | **95% CI** | **95% CI** |  |  |
|  | ***β!***^†^ | **S.E.** | ***t*** | *LL* | *UL* | ***p*** | **VIF** |
| Constant |  | .36 | 16.05 | 5.13 | 6.56 | .001 |  |
| News literacy | -.21 | .01 | -6.91 | -.13 | -.07 | .001 | 1.48 |
| Signal detection | -.33 | .06 | -11.40 | -.84 | -.60 | .001 | 1.31 |
| Reflective thinking | -.07 | .03 | -2.41 | -.12 | -.01 | .02 | 1.13 |
| Tolerance for ambiguity | -.05 | .09 | -1.93 | -.34 | .00 | .05 | 1.08 |
| Drive for information | .32 | .03 | 11.24 | .31 | .44 | .001 | 1.29 |
| Age | -.19 | .00 | -6.88 | -.02 | -.01 | .001 | 1.20 |
| Income – low | -.21 | .09 | -6.60 | -.76 | -.41 | .001 | 1.55 |
| Income – middle | -.13 | .09 | -4.48 | -.55 | -.22 | .001 | 1.34 |
| Leans Independent | -.02 | .09 | -.55 | -.22 | .12 | .59 | 1.40 |
| Leans Conservative | -.04 | .09 | -1.45 | -.29 | .04 | .15 | 1.42 |
| Leans other | -.04 | .13 | -1.34 | -.44 | .08 | .182 | 1.26 |
| *R^2^* = .46; *Adj. R^2^*.45  Income – high and Leans Liberal were excluded due to collinearity.  ^†^Standardized betas shown. | | | | | | | |

| **S6 Table**  *Pre- and Post-test Paired Samples t-test Results for Test Group* | | | | | | |
| --- | --- | --- | --- | --- | --- | --- |
|  |  | Mean | S.D. | *t* | *p* | Cohen's *d* |
| Detection | Pre-test | 5.64 | 1.54 | 3.07 | .001 | .15 |
|  | Post-test | 5.90 | 1.71 |  |  |  |
| Clicking | Pre-test | 3.04 | 1.17 | 3.69 | .001 | .27 |
|  | Post-test | 2.90 | 1.21 |  |  |  |
| Reading | Pre-test | 3.05 | 1.14 | 4.63 | .001 | .32 |
|  | Post-test | 2.88 | 1.21 |  |  |  |
| Sharing | Pre-test | 2.89 | 1.29 | 3.29 | .001 | .25 |
|  | Post-test | 2.76 | 1.34 |  |  |  |
| N = 429  Detection means reflect clickbait and non-clickbait headlines  Engagement means reflect clickbait headlines only | | | | | | |

| **S7 Table**  *Pre- and Post-test Paired Samples t-test Results for Control Group* | | | | | | |
| --- | --- | --- | --- | --- | --- | --- |
|  |  | Mean | S.D. | *t* | *p* | Cohen's *d* |
| Detection | Pre-test | 5.68 | 1.62 | 1.80 | .036 | .09 |
|  | Post-test | 5.84 | 1.73 |  |  |  |
| Clicking | Pre-test | 2.91 | 1.20 | 1.89 | .03 | .19 |
|  | Post-test | 2.84 | 1.26 |  |  |  |
| Reading | Pre-test | 2.87 | 1.18 | 1.54 | .062 | .17 |
|  | Post-test | 2.82 | 1.24 |  |  |  |
| Sharing | Pre-test | 2.71 | 1.33 | .54 | .30 | .12 |
|  | Post-test | 2.70 | 1.40 |  |  |  |
| N = 428  Detection means reflect clickbait and non-clickbait headlines  Engagement means reflect clickbait headlines only | | | | | | |

| **S8 Table**  *ANOVA Results for Clickbait Engagement Parameters*^†^ | | | |
| --- | --- | --- | --- |
|  |  | Test Group | Control Group |
| Clicking | Pre-test mean | 3.04 (1.17) | 2.91 (1.20) |
|  | Post-test mean | 2.90 (1.21) | 2.84 (1.26) |
|  | *F* | 2.34 |  |
|  | *ηp2* | .003 |  |
| Reading** | Pre-test mean | 3.05 (1.14) | 2.87 (1.18) |
|  | Post-test mean | 2.88 (1.21) | 2.82 (1.24) |
|  | F | 5.88 |  |
|  | *ηp2* | .01 |  |
| Sharing* | Pre-test mean | 2.89 (1.29) | 2.71 (1.33) |
|  | Post-test mean | 2.76 (1.34) | 2.70 (1.40) |
|  | *F* | 4.47 |  |
|  | *ηp2* | .01 |  |
| ^†^Analysis used pre- and post-test means differences as dependent variables  **p* < .05; ***p* < .01  Standard deviations shown in parentheses  Means reflect clickbait headlines only | | | |
