# Supplementary material for: SHOCKING! College professor reveals hidden secrets of news headlines. Can you guess? Examining the efficacy of clickbait detection training and news literacy
Source: PLoS One. 2026 Nov 5;21(11):e0359401. doi: 10.1371/journal.pone.0359401 (PMC13739131; doi:10.1371/journal.pone.0359401)
Supplement: Supplementary file 3 [file pone.0359401.s002.docx]

Thanks for the opportunity to review this manuscript. Authors should take note of the following and revise the manuscript.

1. This paper explores the identification of clickbait, yet its title adopts the typical rhetoric of clickbait itself such as "SHOCKING!", "Hidden Secrets", and "Can You Guess?". This style is seriously inconsistent with the rigor of academic research and creates a logical contradiction, which may lead readers to question the seriousness of the study.

Although the author clearly points out in the introduction that such a headline is "deceptive" and inconsistent with the facts, continuing to use this title would mean that the author has personally created a "deceptive" case that will soon be criticized in the main text. This is logically contradictory in academic terms.

2. Some scholars have already discussed the relationship between media literacy and clickbait. It is suggested that the introduction should clearly delineate the conceptual boundaries between news literacy and media literacy, and elucidate how news literacy, as a subset of media literacy, with its unique "news verification skills," provides distinctive explanatory power for clickbait detection, thereby more precisely identifying the research gap.
3. The introduction and literature review sections rely excessively on early literature published over 10 years ago, failing to adequately reflect the latest research advancements in this field from the past ten years
4. The literature review has pointed out that the effect of clickbait is significant in polarized issues, but the research hypotheses did not incorporate news issue type as a moderating variable, leading to a disconnect between the theoretical framework and the literature findings.
5. The selection of experimental materials only considered the temporal dimension to avoid memory bias, but failed to control for or analyze the horizontal domain distribution of news headlines such as polarized issues vs. neutral issues, which may allow domain differences to become potential confounding variables.
6. The research findings currently remain primarily at the level of descriptive statistics, lacking in-depth theoretical interpretation of the relationships between variables. In particular, the influence mechanisms of various dimensions of personal locus on news literacy have not been sufficiently discussed. It is recommended that the authors integrate relevant theoretical frameworks to thoroughly analyze the logical implications and theoretical contributions behind these findings.
