# Supplementary material for: SHOCKING! College professor reveals hidden secrets of news headlines. Can you guess? Examining the efficacy of clickbait detection training and news literacy
Source: PLoS One. 2026 Nov 5;21(11):e0359401. doi: 10.1371/journal.pone.0359401 (PMC13739131; doi:10.1371/journal.pone.0359401)
Supplement: Supplementary file 4 [file pone.0359401.s005.docx]

**Reviewer #1**

Reviewer #1: The manuscript “SHOCKING! College Professor Reveals Hidden Secrets of News Headlines. Can You Guess?” (PONE-D-26-01307) investigates the correlates of news clickbait headline detection and interactions and investigates the effects of literacy training intervention using a cross-sectional sample. This is a very interesting and timely study on clickbait headlines. I hope these comments, provided in no particular order, are helpful to the authors:

Overall, the literature is engaged and discussed comprehensively, and multiple factors are examined in explaining clickbait related outcomes. While this is good, more focus can be placed on the theoretical underpinnings of the training intervention. The intervention has multiple components such as information provision and quiz. These can be discussed in relation to recent research on literacy training interventions on related fields such as misinformation and news engagement (Kuru, 2025, Literacy training vs. psychological inoculation?; Amazeen and Krishna, 2024, Refuting misinformation: Examining theoretical underpinnings; Badrinathan, 2021, Educative Interventions to Combat Misinformation). Discussion section can elaborate the theoretical implications further as well by taking into account exact mechanisms or targets the different components in interventions aim for.

**Response**: I have incorporated the suggested literature both within the front matter and in the discussion. In the front matter, I tie inoculation training to news literacy training – se highlight on page 9 (lines 1-3). In the discussion, I expound on how this study opens a venue to study inoculation and how both inoculation training and clickbait training offer practical uses – see highlight on page 29 (lines 1-2).

Relatedly, in the methods section, intervention can be elaborated/described in greater detail.

**Response**: I have revised this section to elaborate on the stimuli, headline selection, and procedure. See highlight on page 14 (lines 16–21) and page 15 (linesn1–24).

In the experiment, measurement order of outcome variables is as follows: participants first evaluated whether the headlines they have seen were clickbait or not, and then proceeded to answer the other outcomes (clicking on the headline, reading, or sharing the news story). This initial asking about clickbait can introduce experimenter demand effects for the questions that come after: clicking on the headline, reading, or sharing the news story. If anything, it may suppress effects on these three subsequent outcome variables; but there are significant effects observed on those as well. Hence, this should be carefully discussed, and perhaps some question order related methodological literature can be cited (Rasinski, Lee, and Krishnamurty, 2012, Question order effects).

**Response**: This is a legitimate concern that I had not addressed earlier. When designing the survey, I used blocks for each headline and also for the survey questions. Therefore, the subjects viewed the clickbait and non-clickbait headlines randomly – I mention this in the procedure section (. What I did not mention is that they also viewed the survey questions randomly, so no one subject viewed headlines and questions in the same order. I have since addresses this issue in the limitations section – see highlight on page 31 (lines 1–8).

While this outcome measurement issue is relevant for all respondents, for experimental news literacy training group, extra care was shown to not even use the word “clickbait”: “To prevent priming the subjects, the tutorial did not mention the word clickbait” which is good.

Response: Not applicable

More sampling details such as pseudo response rate, removal of respondents criteria can be helpful.

**Response**: The Qualtrics panel that I used had a 100% completion rate. Only those who failed the attention check questions were eliminated. See highlight on page 12 (lines 15–16).

As far as I understand, survey and experiment come from the same dataset. Where was experimental manipulation placed?

**Response**: I had not discussed this issue before I do. The in-study quiz during the main experiment acted as the manipulation check. see highlight on page 19 (lines 5–13).

Are any survey analyses use measures obtained after the experimental manipulation during the survey? I assume not, since they are referred to as pre-test measures. Still, in such a study, this should be very clearly stated. Anything that comes after experimental manipulation (control and training) should not be used in the initial correlational part of the empirical evidence/results.

**Response**: No, none of the survey-based variables were tested or measured after exposure. I’ve tried to say this in various places but each time it ended up reading very awkwardly. Therefore, I added a sentence at the beginning of the results section – See highlight on page 19 (line 18): *Hypotheses 1–5 were tested based only on data collected in the pre-test.*

Relatedly, I also wonder if the measure of news literacy questions might have primed/sensitized respondents to focus on clickbait headlines critically above and beyond the news literacy training in the experimental module. This should be discussed and potentially, it can be used as a moderator. Not a theoretical moderator, but as a methodological test to understand if respondents who were at baseline either weak or strong in news literacy behaved similarly in their uptake of the intervention (clickbait news literacy training).

Response: I get the gist of the concern, but I do not fully understand what I need to do or how to it. Did the reviewer mean to say clickbait training rather than news literacy training? If referring to the news literacy questions, these are very general and they query about broad aspects of the media industry, and not about specific such as how headlines are written. In fact, the only question that comes close to that refers to the news lead. It is unlikely that the news literacy questions may prime subjects to be critical of the media more than they are because research suggests that news iterate people are better at analyzing headlines by default. Additionally, subjects were never informed if they got the news literacy survey questions right or wrong.

If referring to the clickbait training, running it as a moderator would only apply to half the sample, those who were exposed to this intervention.

Was the randomization successful? Pretest balance checks can be helpful (on all relevant demographic and theoretical variables, such as news literacy, reflective thinking etc.).

**Response**: I ran these statistics and there were no significant difference between the test and control group in any of the survey-related variables. See highlight on page 13 (lines 5–8) and supplemental tables 1 and 2, where I compared the two groups regarding demographics, and here too there were no differences.

Relatedly, looking at the means in Table 6 and 7, I notice that experimental condition’s pre-test values seem to be higher for some reason. This can be by chance, but it should be tested whether this is significant. If so, these pre-test values should be included as a control variable in an ideally OLS or ANCOVA model when groups are compared directly (when not using the pre-test DV variables).

**Response**: In my revision, I use ANOVA to analyze the pre- and post-test detection and engagement variables.

Was there manipulation check for the experiment? It is my understanding that in both control and training manipulation, participant answered a set of quiz questions and all were shown correct answers. Is the data for whether participants answered correctly or incorrectly available. If so, this can be used as an in-built manipulation check too perhaps.

**Response**: yes, the in-study quiz was the manipulation check. See highlight on page 19 (lines 5–13).

-------------

**Reviewer #2**

Reviewer #2: Thanks for the opportunity to review this manuscript. Authors should take note of the following and revise the manuscript.

1.This paper explores the identification of clickbait, yet its title adopts the typical rhetoric of clickbait itself such as "SHOCKING!", "Hidden Secrets", and "Can You Guess?". This style is seriously inconsistent with the rigor of academic research and creates a logical contradiction, which may lead readers to question the seriousness of the study.

Although the author clearly points out in the introduction that such a headline is "deceptive" and inconsistent with the facts, continuing to use this title would mean that the author has personally created a "deceptive" case that will soon be criticized in the main text. This is logically contradictory in academic terms.

**Response**: It’s more strategic than contradictory. I chose this title strategically to segue into the introduction and have the reader first view a detailed definition of clickbait, one that also gives examples of forward-references, emotionally charged words, and question headlines. This is something I pondered lengthily. Also, the title is not deceptive and reflects plenty of titles used in clickbait studies:

- Jung AK, Stieglitz S, Kissmer T, Mirbabaie M, Kroll T (2022) Click me…! The influence of clickbait on user engagement in social media and the role of digital nudging. PLOS ONE 17(6): e0266743. <https://doi.org/10.1371/journal.pone.0266743>
- Scott, Kate. “You Won’t Believe What’s in This Paper! Clickbait, Relevance and the Curiosity Gap.” Journal of Pragmatics [Amsterdam], vol. 175, April 2021, pp. 53–66, https://doi.org/10.1016/j.pragma.2020.12.023.
- Béna J, Rouard M, Corneille O. You won't believe it! Truth judgments for clickbait headlines benefit (but less so) from prior exposure. Applied cognitive psychology. 2023;37(6):1418–29.
- Shrestha A, Behfar A, Al-Ameen MN. “It is luring you to click on the link with false advertising” - mental models of clickbait and its impact on user’s perceptions and behavior towards clickbait warnings. International Journal of Human–Computer Interaction. 2024;41(4):2352–70.
- ‘I Urge You To See This...’. Clickbait As One Of The Dominant Features Of
- Contemporary Online Headlines.
- Mukherjee P, Dutta S, De Bruyn A. Did clickbait crack the code on virality? Journal of the Academy of Marketing Science. 2022;50(3):482–502.

2.Some scholars have already discussed the relationship between media literacy and clickbait. It is suggested that the introduction should clearly delineate the conceptual boundaries between news literacy and media literacy, and elucidate how news literacy, as a subset of media literacy, with its unique "news verification skills," provides distinctive explanatory power for clickbait detection, thereby more precisely identifying the research gap.

**Research**: I have addressed this issue by emphasizing the association between news literacy and media literacy generally, but also specifying the association between news literacy and the four media literacy variables. See highlight on page 3 (line 23–24) and page 4 (lines 1–5).

3.The introduction and literature review sections rely excessively on early literature published over 10 years ago, failing to adequately reflect the latest research advancements in this field from the past ten years

**Response**: Most of the dated references were in the introduction and the *Effects of Clickbait* sections. I have either removed some of the old references and replaced them with newer sources or added newer references to the old ones. The few dated references I left were either important survey data or seminal studies such as Blom and Hansen (2015) and Shostak and Gillespie (2014). I have incorporated the following:

- Li X, Zhou J, Xiang H, Cao J. Attention grabbing through forward reference: an ERP study on clickbait and top news stories. International Journal Of Human-Computer Interaction. 2024;40(11):3014–29.
- Enache A, Militaru M, Ioncica D. The Emotional Appeal of Clickbait Headlines – between Entertainment and Deception. Dialogos. 2022;
- Su Y, Lee DKL, Xiao X. "I enjoy thinking critically, and I'm in control": Examining the influences of media literacy factors on misperceptions amidst the COVID-19 infodemic. Comput Human Behav. 2022 Mar;128:107111. doi: 10.1016/j.chb.2021.107111. Epub 2021 Nov 27. PMID: 34866771; PMCID: PMC8631744.
- Shrestha A, Flood A, Sohrawardi S, Wright M, Al-Ameen MN. A First Look into Targeted Clickbait and its Countermeasures: The Power of Storytelling. Proceedings of the 2024 CHI Conference on Human Factors in Computing Systems; Honolulu, HI, USA: Association for Computing Machinery; 2024. p. Article 971.
- Sundar SS, Snyder EC, Liao M, Yin J, Wang J, Chi G. Sharing without clicking on news in social media. Nature Human Behaviour. 2025;9(1):156–68.

4. The literature review has pointed out that the effect of clickbait is significant in polarized issues, but the research hypotheses did not incorporate news issue type as a moderating variable, leading to a disconnect between the theoretical framework and the literature findings.

**Response**: This is a good observation, but the type of news that the clickbait headline accompanied was not within the focus of the study. I have mentioned this in the limitations section. See highlight on pages 30 (lines 20–23).

5.The selection of experimental materials only considered the temporal dimension to avoid memory bias, but failed to control for or analyze the horizontal domain distribution of news headlines such as polarized issues vs. neutral issues, which may allow domain differences to become potential confounding variables.

**Response**: This too is a good observation, but the valence of the clickbait headline was not within the focus of the study. Like with the news types or issues, have mentioned this in the limitations section. See highlight on page 30 (lines 20–23).

6.The research findings currently remain primarily at the level of descriptive statistics, lacking in-depth theoretical interpretation of the relationships between variables. In particular, the influence mechanisms of various dimensions of personal locus on news literacy have not been sufficiently discussed. It is recommended that the authors integrate relevant theoretical frameworks to thoroughly analyze the logical implications and theoretical contributions behind these findings.

**Response**: This reflects the writing style in my field, where we present results in an objective manner and then contextualize them in the discussion section. While I had done this before, I have now expanded my discussion on the confluence among news literacy and the personal locus variables See highlight on page 26 (lines 12–23).

-----------

**Reviewer #3**

Reviewer #3: The paper addresses an important and timely question regarding news literacy, cognitive dispositions, and engagement with clickbait content. However, in its current form, the manuscript requires major revisions before considering its publication.

1, The experimental results rely primarily on within-group pre–post comparisons. However, the key inferential question is whether the change over time differs between the intervention and control groups.

Without directly testing the time × condition interaction, it is not possible to conclude that the training had a distinct causal effect. The manuscript should therefore include an appropriate interaction model (e.g., mixed ANOVA or regression with interaction term) and report effect sizes and confidence intervals.

**Response:** I have since rewritten my description of the experiment to clarify that it was a single factor between subjects design. The single factor was clickbait training with two levels (clickbait training vs. placebo training). See highlight on page 12 (lines 7-12).

**Response**: To address the time issue, I have now included results from ANOVA analysis that used mean differences between the pre- and post-test scores and a dichotomous variable for test group vs. control group a done in previous studies single factor, two-level studies.. See explanation on page 24 (lines 16–20).

2, Please provide a link to state that the referred data is publicly available for the readers.

**Response**: I had already provided this statement and a link to the data during the submission process form even though it does not appear in this version of the manuscript. I think the publisher will include the link once the paper in available online.

3, Clicking, reading, and sharing intentions are extremely highly correlated. Treating them as separate dependent variables in multiple regressions may inflate statistical significance and obscure whether they reflect a single underlying engagement construct. The authors should clarify whether these are theoretically distinct constructs or facets of a broader engagement dimension, and adjust the analysis accordingly (e.g., composite index, multivariate modeling, or correction for multiple testing).

**Response**: I have since combined these three into a composite engagement variable. For the sake of nuance, I have included data on the three separate parameters separately as supplemental tables.

4, The regression models include key predictors but appear to omit potentially important demographic and media-use controls (like age, education, ideology, general news use). Please consider including relevant control variables and report whether the main findings remain robust. This would strengthen causal interpretation and reduce concerns about omitted variable bias.

**Response**: I have redone the regression and included age, income, and ideology. The survey did no collect education data because the study did not predict this variable. I have also included a discussion on the effect of these demographic variables in the discussion section. See highlight on page 28 (lines 4–16).

5, The manuscript would benefit from stronger documentation of how clickbait vs. non-clickbait headlines were identified and validated.

Please clarify:

-Whether independent coders classified the headlines and reported intercoder reliability (if applicable).

-Whether a manipulation check or pilot test confirmed that participants perceived the headlines as clickbait.

-How ecological validity was considered (e.g., absence of images, platform cues).

-Including headline examples in an appendix would improve transparency.

**Response**: As I mention in the method section, I used clickbait headlines from a previous study (see below) whose authors created them from scratch and validated them in a small sample pilot test. For my study, I randomly picked real headlines and tested them in a pilot study similar to the Béna, et al. approach. I have now included a more detailed description of this process. See *Headline Selection* section on page 14 (lines 2–14)..

Béna J, Rouard M, Corneille O. You won't believe it! Truth judgments for clickbait headlines benefit (but less so) from prior exposure. Applied cognitive psychology. 2023;37(6):1418–29.

6, The scoring procedure for the signal detection task should be described more clearly and consistently.

**Response**: I have revised this section for clarity. See highlight on page 17 (lines 5-7).

7, Please consistently distinguish between survey (pre-test) analyses and experimental findings.

**Response**: I have included statements to differentiate between the two on page 19 (line 18) and on page 23 (lines 13–14).

8, Report effect sizes and confidence intervals more systematically.

**Response**: I have included the effects sizes and confidence intervals when applicable. See tables 2 and 3 and supplemental tables 3–5 for confidence intervals. Supplemental tables 6–7) show effects sizes for t-test and ANOVA results.

9, Polishing the clarification of statistical reporting would improve readability.

**Response**: Done

------------

**Reviewer #4**

Reviewer #4: This is a very relevant topic for the times we live in. The paper is written well and the research is clearly embedded in literature and discussed thoroughly.

I have only a couple of minor comments (see attached file).

-----------

The purpose of the review is to provide the editors with an expert opinion regarding the validity and quality of the manuscript under consideration. The review should also supply authors with explicit feedback on how to improve their papers so that they will be acceptable for publication in *PLOS One*. As you write, consider the following points:

- What are the main claims of the paper and how significant are they for the discipline?
  - The study examines the effects of news literacy and a clickbait training on clickbait detection and clickbait behavior. The author found that readers who are news literate are less likely to engage in clickbait behavior. Additionally, a brief training seemed to improve clickbait detection and decrease clicking. It seems that the American public (based on the sample used) is not very news literate. The subject of this paper is

certainly relevant in today’s climate of polarization, where accusations of fake news are

being used widely and wildly to discredit and silence.

**Response**: Not applicable

- Are the claims properly placed in the context of the previous literature? Have the authors treated the literature fairly?
  - The study is embedded in the context of previous literature well.

**Response**: Not applicable

- Do the data and analyses fully support the claims? If not, what other evidence is required?
  - The data and analyses show significant relationships between literacy and clickbait detection. The brief training showed improvement in detecting clickbait. The author does draw causation with data that only shows correlation (discussion, line 7 of page 25), which needs to be revised. The statement “news literacy…directly improved detection” is not correct based on the analyses. Correlation does not equal causation…

**Response**: This statement refers to news literacy and not clickbait detection training. It also refers to the pre-rest data. Even then, the reviewer is correct because as written, the statement does not explicitly point out that this conclusion is based on both the correlation and regression analysis results In fact, news literacy was correlated with clickbait detection (*r* = 29, p < .001) but it also had a main effect on detection in the pre-test (*β*  **= .**22**, *p* < .**01). I have revised it to mention the main effects from the regression analysis. See highlight on page 19 (lines 22–24)..

- Are details of the methodology sufficient to allow the experiments to be reproduced?
  - Yes. As a minor point, Table 1 is not formatted correctly; the M and SD columns belong on the right-hand side.

**Response**: I have revised the table.

- Is any software created by the authors freely available?
  - N/A
- Is the manuscript well organized and written clearly enough to be accessible to non-specialists?
  - Yes. The paper is written very clearly.
- Is it your opinion that this manuscript contains an [NIH-defined experiment of Dual Use concern](https://grants.nih.gov/grants/guide/notice-files/NOT-OD-13-107.html)?

Although confidential comments to the editors are respected, any remarks that might help to strengthen the paper should be directed to the authors themselves.

**Reviewer #5**

Reviewer #5: Overall, the design of the experiment and survey is sound and built on previous work in this area. The application of media literacy to news literacy and the underlying psychometric measures are well-defined, and the reasoning for the statistical analysis of OLS regression is sound. I greatly appreciated the thorough explanations of the variables in the background information and examples of questions from the surveys.

The only revisions needed are to include the tools used to do the analysis (SPSS, R?),

**Response**: I used SPSS, and I have now included a statement on that. See highlight on page 19 (Line 13).

Some awkward descriptions of variables on pg.6 12-16 (emotionally charged words and engagement),

**Response**: I have revised this section. See highlight on page 6 (lines 16_22).

and a discussion of the analysis of demographic information.

**Response**: I have done this in several ways:

- Because I now include the demographic variables I the regression analysis, I have added a section discussing their impact on the dependent variables. See last paragraph of the discussion section, page 29 (lines 4–16).
- I have also incorporated a new comparison of demographics between the test and control groups. See supplemental tale 1. I also contextualize this comparisons on page 13 (lines 5-9)

The author points to the care of producing a sample that reflects the diversity of the larger population, but does not include how the factors of gender, ethnicity, race, age, etc may interact with the other variables to predict news literacy and clickbait detection. If these factors offer no predictability to the model, then that should be stated.

**Response**: I have now included these demographics variables in the regression analysis. See tables 1 and 2 and supplemental tables 3–5.

-------------

**Reviewer #6**

Reviewer #6: Dear authors,

I was very pleased to review your manuscript. This is a well-written manuscript that I reviewed so far this year. The paper provided sound background indicating gaps in the literature and its broad theoretical implications. The most important aspect of the manuscript it's the combination of the survey and experimental design. Relevant analyses were conducted to support the hypotheses. The discussion of the results was adequate. The overall quality of the writing is good and simple. After back and forth examination of the paper, I found no substantial issues that would prevent me from recommending it in its current form. Thus, I opt for an "ACCEPT" decision.

It was nice reading your work.

Regards

**Response**: Not applicable

------------

**Reviewer #7**

Reviewer #7: See attached review. This box has a minimum length, so I include the initial paragraph but ask that the full review be considered.

The paper presents the results of a survey and experimental intervention into 'clickbait engagement'. The study reports evidence in line with most of the current literature, but develops this evidence through an experimental framework to extend our confidence in the current understanding. The paper drew my immediate curiosity as to the state of objective measures of clickbait. But I am uncertain about the state of agreement as to these definitions -- it seems likely that one person's clickbait is another person's viral content; are there agreed-upon thresholds about the difference between clickbait and good marketing? I can see deceptiveness as an area where there would be more agreement. How does one distinguish between a headline with a question that is also clickbait, and one that isn't? In general this line of inquiry may be quite settled in the area of clickbait studies, but I think for a PLOS audience it would be helpful to take a slightly more expository approach. In particular I was not sure how to think about non-deceptive clickbait, and I notice that the Instrument in the appendix does not seem to cover this category and the text unfortunately contradicts itself on this concept. I also had trouble with the way the concept of personal locus is explained throughout; more guidance would be helpful here.

This is strong work but would benefit from revision, particularly with respect to how it engages the concepts under study. A clearer conceptual framework and the relationship of constructs to one another would allow this work to be placed more firmly in the literature. I organize my review to match the ordering of the work, noting various minor points at the end.

----------

Abstract and Introduction:

.......................

The work describes personal locus as "a competency adjacent to news literacy," without saying what personal locus actually is. Given how important this concept is for the work, I think it's important to really nail this. The Introduction / line 18-19 on page 3 get a bit closer to offering a definition, but I still don't feel like I quite grasp it or know what it means from what's presented here. I did a bit of searching on the definition to ground myself (seemingly, belief that one has control over one's life), but I think it would aid the reader to be supplied with a definition and reference in the first 2 pages since it's a key concept in the work. In general, more clarity about concepts would be helpful in the introduction and elsewhere as noted.

**Response**: I had defined personal locus, but that was further down in the paper under the *News Literacy and Personal Locus* section. I have now defined it earlier in the introduction and also rewritten the passage where I defined it earlier to make it more obvious and better tie it to media literacy and news literacy. See highlights on page 3 (lines 23–24) and page 9 (lines 1–6).

The effects of clickbait

.......................

I think this next section is related work or background -- standard section headers are likely not required by this venue but do tend to aid the reader.

The extended discussion of clickbait as a phenomenon was interesting to read and resonated with me; it would be useful to expand on this by including some sense of the degree of agreement people have about what is and isn't clickbait and generally establish the validity of the construct.

**Response**: While there is a consensus on the definition of clickbait and the characteristics of clickbait headlines, declaring a certain headline to be clickbait is subjective and changes from person to person. It is for this reason that I focused my discussion on the characteristics of clickbait and gave examples from seminal studies like Blom & Hansen and Jodłowiec.

**Response**: Regarding validity, I had not discussed the validity of the clickbait and non-clickbait headlines used here. I have now done so, and this also answers the concerns the reviewer raised in their initial comments. See highlight in method section – pages 14 (lines 1-14).

Additionally, the issue of deceptive clickbait is important, but that was beyond the scope of the current study.

Pages 5 and 6 describe the current state of research about clickbait with respect to numerous measures. Although this material really is interesting, I think at times it veers away from the analytical focus of the current work.

**Response**: This section serves several purposes. First, it explains the nuances of clickbait beyond the definition and gives the reader a better picture of what clickbait is given the subjectiveness of determine what is and what not clickbait. The examples of forward-references and emotionally charged words help clarify how clickbait headlines are presented. The discussion on the effects of clickbait in this section also ground in theory, the hypotheses that predict the effect of exposure to clickbait.

The role of news headlines

.......................

I'm not sure a study demonstrating that a deceptive headline has consequences (page 7 line 14) is evidence that a full reading is required in the case of a non-deceptive headline (line 15).

**Response**: The study cited here found that people made judgments about the subject of an opinion news story based on a headline that was misleading or incongruent with the story itself. This suggests that a full reading of the story is needed to get the true information. I have changed the wording to that shown below. See highlight on page 7 (lines 15–17).

*“For instance, a misleading headline accompanying an opinion article may affect the reader’s judgement about the story’s subject even when that headline is incongruent with the gist of the article.”*

I am not sure what "the sentiment and context with which a headline is written" means (page 7 line 17); the lines of text following this statement only seem to talk about sentiment.

**Response**: Tis refers to the valence and effect of the words, negative or positive. I have replaced the word sentiment with valence and affect. See highlights on page 7 (lines 19–22).

News Literacy and Personal Locus

.......................

Here I find I am a little bit confused again about the concepts under study; news literacy is clear enough, but now I see a conflicting conceptual relationship expressed. In line 5 and again on line 12, it sounds as if the definition of personal locus is actually a sub-concept inside of a media literacy framework, but I am finding this difficult to understand as the concept expressed in some literature is quite a bit larger -- it seems to be a more general feature of people, with implications for many things, including their media literacy and news literacy. Perhaps a conceptual diagram along with directly addressing the relationships among concepts would help?

**Response**: Locus of control is the larger, broader concept. Personal locus was the specific concept coined by Potter (2004) in his discussion of media literacy. To refer to one’s control over their media use. In my revision on page 8 (9–14), I tr to connect these three concepts.

The engagement with Dewey is nice to see here.

It's a matter of taste, but in a complex series of hypotheses (there are 24 of them if you include the letters), my preference as a reader is to have the hypotheses positioned within the literature review, such that there is a direct bright line between "Literature says..." and "H# proposes" without me needing to scroll back and forth in the section.

Alternately the ordering of the concepts in the hypotheses could match up the ordering of the background; for example I see that the lit about detection training is discussed in the middle of the section, but it's the last hypothesis.

**Response**: I have rearranged the hypotheses as suggested. To allow for a logical discussion of the results, I left H6 as the last one given that H1–H5 predict about the four personal locus variables. It would be confusing to present the clickbait training results in between the results of the personal locus variables.

Method

.......................

It would be helpful to point back to the hypotheses by number here in the method and measures section to aid the reader in tracking the argument.

I was not sure why synthetic headlines were used for clickbait and real but old headlines used for non-clickbait; the opening argument of the paper describes the BBC as having clickbait so presumably so might the NYT or AP. Presumably either or both could be used, but using a different procedure for one versus the other is unclear to me.

**Response**: Ia have provided a more detailed explanation of how and why the headlines were collected. See the Headline Selection section in the methodology – pages 14 (lines 1–14).

I was surprised to see the signal vs noise exploration collapsed down to distinguishing advertorials; the background seems to frame this much more broadly (albeit briefly -- I think just lines 8-12 on page 16?

**Response**: It’s true that signal detection may refer to a broad range of materials. In the literature, I discuss the Tully, et al. study that emphasized that news-literate people need to distinguish between news content from other content like opinions and advertisements. I used advertorials because they deceptively mimic editorial content and research shows that this make them more engaging, suggesting that it takes a higher level of signal detection ability to distinguish them from traditional advertisement content. I have added this explanation in the method section. See highlight on page 17 (lines 5–7).

Results

................

It would be useful to have more interpretation of the empirical results -- particularly the practical significance of the statistically significant factors. Some visualizations showing relative effects at a glance would be useful as well; otherwise one has to scroll back and forth to weigh out these factors.

**Response**: Reviewer 2 also brought up this issue. I explained that in my filed, we present the results in an objective manner and contextualize and interpret them in the discussion section. I have since expanded my previous discussion of the significant and non-significant results. See highlights on pages 24–25).

Discussion

................

I would like to see more exploration here of the contradicted / failed hypotheses -- what might be the implications of this reversal?

**Response**: I have expanded on potential reasons to explain this failed hypothesis. See highlight on page 27 (lines 11–17). And page 27 (lines 18–23) and page 29 (line 1–4).

Implications and Limitations

................

The phrase "expanding the avenues of scholarly inquiry" is vague as a summary of contribution; presumably every study does this?

**Response**: I have now specified that it opens avenue to psychology and misinformation susceptibility research. See highlights on page 29 (lines 20–21) and page 30 (lines 4–8).

The section says that "clickbait uses deception" but the opening definition states that it can be both deceptive and non-deceptive?

**Response**: This was supposed to read – Because some clickbait uses deception. I have made the revision. See highlight on page 30 Line 3).

Document preparation

.......................

I saw some unevenness here, with stray / missing punctuation and spacing. I list the items I noted as I read, but the quantity suggests the need for a thorough proofread.

Line 18 / page 3 would be clearer with an Oxford comma (otherwise, it's unclear as to whether this is a list of three measures, or if the work is defining personal locus as "a psychometric measure of media literacy ...and misinformation susceptibility". I was sufficiently confused that I re-read this bit repeatedly -- up until I realized it was simply an ambiguity, rather than a phrase offering an inline definition.

**Response**: Done

odd punctuation in p. 4 line 14 -- there's an unpaired double quote, and an unpaired parenthesis.

stray punctuation -- line 18 of page 6

stray spaces -- page 7 line 12

p.9, "differentiating between signal from noise" -- unclear use of idiom.

Some missing spaces and an extra comma (p 9, line 14 and 21)

Unclear: "The reflects the self-awareness" (p10. line 10)

Missing period: (p 10 line 18)

"discission" (p10 line 22)

Usually I see a colon at the end of lines like line 22, p 10.

Extra comma, p 15 line 6

Stray letter, p 16 line 3

"Hypotheses 5" (p 23 line 10)

"news literacy write large" (p 25 line 16)

"which exclusively composed" (p 26 line 20)

**Response**: Done

References

.......................

Some formatting errors make this section at times difficult to read (missing italics, missing punctuation, missing publication name).

--------------

**Reviewer #8**

Reviewer #8: Thank you for the opportunity to read this manuscript. I really enjoyed it, and I think it would make a great candidate for inclusion as a PLOS one article. I do have some minor comments that I believe will make the manuscript stronger prior to publication, but that amount to curiosity and a few minor details that may reflect the framing/writing of the text rather than a weakness in the work itself. Comments below, presented in no particular order:

- I'm interested in seeing a bit more discussion of the demographic makeup of your sample. You mention that your total sample is representative of the US population, but does this mean that both your test group and your control group were representative samples? Were the percentage of respondents in each demographic category the same, or were there differences? This isn't clear in the text, so if both groups were representative samples, I would suggest making that a bit clearer in the text.

**Response**: I did not design each group to be representative Rather; I recruited one large that was representative and then randomly assigned some to the test group and some to the control group. The randomization was successful because there were no significant differences between the two groups among the demographic variables and among the survey-related predictor variables. I have included a passage on this. See highlight ton page 13 (lines 5–8) and supplemental tables 1 and 2..

**Response**: Below is a comparison of the demographics between the test and control groups. This table appears in the paper as Supplemental Table 1.

| Comparison Between Test and Control Group Demographics | | | | |
| --- | --- | --- | --- | --- |
|  |  | **Test Group** | **Control Group** | ***p*-value** |
| Age |  | 42.11 | 41.68 | .7 |
| Gender |  |  |  | .25 |
|  | Male | 213 | 199 |  |
|  | Female | 216 | 227 |  |
|  | Non-binary | 0 | 2 |  |
| Ideology |  |  |  | .27 |
|  | Leans Independent | 133 | 113 |  |
|  | Leans Liberal | 125 | 148 |  |
|  | Leans Conservative | 132 | 133 |  |
|  | Other | 39 | 34 |  |
| Income |  |  |  | .98 |
|  | Less than $50,000 | 136 | 134 |  |
|  | Less than $50,000 | 118 | 117 |  |
|  | More than 100.001 | 175 | 177 |  |
| Test group N = 429  Control group N = 428  Age denotes t-test; gender, ideology, and income denote chi-square test | | | | |
|  | | | | |

- if it is the case that your two groups were comprised of different demographic makeups, I'm curious how that might influence your results, since there isn't very much discussion of demographics in the manuscript. Specifically, I'm wondering about ideological differences in news literacy and vulnerability to clickbait, since there's ample research that deals with formation of political echo chambers and political misinformation. if there were notable demographic differences on your literacy or other metrics, perhaps this is something that could be discussed in a short appendix.

**Response**: I have now included the demographics variables in the regression analysis. Only age and income returned main effects. I contextualize the effect of the demographics variables in the discussion section. See highlight on page 29 (lines 4–16).

- your introduction is at times a bit repetitive, such as for example in the second paragraph of your "effects of clickbait" section, where you mention the definition of forward-referencing despite already describing what forward referencing is in your opening paragraph. I would suggest tightening up the writing a bit here and make sure you aren't repeating yourself, but this is a very minor thing

**Response**: I have revised this part to reduce the repetitiveness. See highlight on pages 4 (lines 12–21).

All in all, I think this is a strong manuscript that would make a great candidate for publication.
