# Supplementary material for: SHOCKING! College professor reveals hidden secrets of news headlines. Can you guess? Examining the efficacy of clickbait detection training and news literacy
Source: PLoS One. 2026 Nov 5;21(11):e0359401. doi: 10.1371/journal.pone.0359401 (PMC13739131; doi:10.1371/journal.pone.0359401)
Supplement: Supplementary file 5 [file pone.0359401.s006.docx]

6. Review Comments to the Author

Please use the space provided to explain your answers to the questions above. You may also include additional comments for the author, including concerns about dual publication, research ethics, or publication ethics. (Please upload your review as an attachment if it exceeds 20,000 characters)

Reviewer #1: Overall, my comments are addressed. Limitations section can be expanded a bit to include a point about having more diverse and comprehensive manipulation check questions in future research. I read other reviewers’ thoughtful and detailed comments and it looks like the revised manuscript improved a lot in clarity, transparency, and methodological details a lot. One comment I have about demographics is that pre-test balance checks shows that randomization worked, hence I think it is unnecessary to have demographic covariates in the analysis, but they can still be retained. This can be noted somewhere in analytical strategy that these covariates are added as exploratory as they are not required for experimental comparison. I think this paper is a good addition to the literature.

**Response**: I have added a note about the manipulation check in the limitations section. See highlight on page 32.

**Response**: I have also added a note about using the demographic variables as controls in the Statistical Analysis section. See the highlight on page 20.

Reviewer #2: Thank you for the revised manuscript and the detailed point-by-point responses. The authors have addressed most of my previous concerns in a serious and constructive manner, and the manuscript has been improved accordingly. In its current form, I believe the paper is generally acceptable for publication.
That said, I would like to retain two reservations for the record.
I appreciate the author's effort to justify the current title by citing prior studies on clickbait that used attention-grabbing wording. However, I remain unconvinced that these examples are fully comparable to the present manuscript title. Several of the cited titles are either more restrained in tone, explicitly framed within an academic subtitle, or use clickbait-like language in a self-referential and clearly scholarly way. By contrast, the current manuscript title appears to reproduce clickbait rhetoric more directly, and thus more closely resembles the very discursive form that the paper critically examines. For this reason, I do not think the cited precedents fully resolve the concern. My reservation is therefore not about the mere presence of an attention-grabbing phrase, but about the degree and manner in which the title adopts clickbait conventions in an academic context.
Second, I appreciate that the authors have now acknowledged the issues of news issue domain and headline valence or emotional orientation in the limitations section. Given the stated scope of the study, this is an acceptable way to handle these factors at this stage. However, I would still note that these are not merely peripheral matters, they may shape clickbait perception and detection in meaningful ways and therefore affect the broader interpretability of the findings.
Overall, these remaining concerns are reservations rather than fundamental objections. The manuscript has made a contribution, and I consider it acceptable for publication.

**Response**: I have since revised the title. Even though the kicker still reads like clickbait (for purposes of illustration), I have added more to the title to make it suitable for a scholarly paper.

Reviewer #3: Thanks for your revisions, which have addressed my concerns.

The last suggestion you might consider:
Although the title of the research article allows for its creativity, it is essential not only to consider its appeal to the audience, but also to maintain the nature of the research, which aligns with the nature of this Journal. Therefore, I would suggest that the author revisit the title and choose a more appropriate one.

**Response**: Similar to my response above, I have since revised the title. Even though the kicker still reads like clickbait (for purposes of illustration), I have added more to the title to make it suitable for a scholarly paper.

Reviewer #5: (No Response)

Reviewer #6: Dear author,

I have read the revised version of your manuscript with great interest. I went through all previous comments of each reviewer. I remarked substantial improvements have been made. Thus, I conclude that the manuscript is good enough to be published in its current form.

Regards

Reviewer #7: I appreciate the high level of effort this revision represents and appreciate all of the engagement among scholars that I see in the lengthy review record. This effort has much improved the work. I prepared my review from the unhighlighted version to try to offer a fresher perspective.

Ultimately I find that several of my key concerns have not been addressed by the revision. My concerns are with respect to the results and their interpretation, as well as the concepts under study. Unfortunately and with due respect to all the labor in evidence, I cannot yet recommend publication given these gaps.

1) Interpretation of results.

To the extent that clickbait training only functions in the limited scope described in the abstract (i.e. when only clickbait is present), I'm not persuaded by the evidence presented here that such training is beneficial. News consumers are faced with an environment that mixes clickbait with non-clickbait, the exception being perhaps the weird clickbait / advertising section one finds at the bottom of even legitimate sites.

I also note that the correlations are relatively small to negligible and the coefficients are all quite small. Descriptions of the practical significance of these effect sizes would be very useful as the units are constructed within the instrument.

**Response**: As I mentioned in the paper, these results may be because this was a cross-sectional study where subjects were exposed to a single session of clickbait detection training. A longitudinal study with multiple training sessions could have produced more robust results. I have added a note about this practicality in the implications section – see highlight on page 31.

2 ) Concepts under study

I find that I am still concerned about the lack of a conceptual map or a way to relate these ideas to one another, and the resulting statistical problems in interpretation. To be clear, this is a fundamental validity question, rather than one of reader clarity.

The text argues that personal locus is "a measure of media literacy associated with news literacy" and misinformation susceptibility; news literacy is also described as a "subset" of media literacy, and "associated with" the 4 personal locus variables. The study tests 5 variables -- the 4 locus variables and a measure of news literacy. However, conceptually these ideas are not presented as independent from one another.

**Response:** My rationale is that extant research suggests that these variables are interrelated, not independent, as the reviewer mentions, but no one has examined these associations, hence the uniqueness of the study. I feel that I have adequately discussed the literature suggesting potential associations, and the results support this conceptualization.

This seems like a source of either multicollinearity, or otherwise turns the locus variables into controls (potentially partial / noisy ones) for news literacy, which makes interpretation challenging and doesn't support the interpretation in use. It does look like the multicollinearity is not bad given the correlations in table 1, but the issue of noisy / bad controls remains -- because then when interpreting the coefficients in table 2, one would say, "a 1-unit increase in news literacy is associated with a .22 unit increase in clickbait detection, holding signal detection, reflective thinking, TfA, and DfI constant", or "two people who are identical in their personal locus but who differ by one unit in their news literacy...". My question is, given the conceptual map as I understand it from the text, if the locus variables are themselves part of news literacy, how should we interpret the regression results, or if the 4 personal locus variables serve as a measure of news literacy, why are both the personal locus variables and news literacy serving as independent variables in the same regression?

**Response**: The four personal locus variables do not measure news literacy, but I argue that they are adjacent to news literacy. I think the confusion arose because of how I introduced media literacy, news literacy, and personal locus. I have revised this section for clarity by emphasizing the chronological order in which these interrelated concepts were introduced. Media literacy in 1992, personal locus in 2004, and news literacy in 2008. In brief, media literacy is the broader concept that was proposed in 1992. Potter reimagined media literacy in 2004, when he introduced personal locus as a key component of media literacy. Allan Miller later introduced news literacy in 2008 – see highlight on page 8.

Then again, later this is also described as a finding of the study rather than an element of the conceptual framework. I am not sure why this is framed as a finding, however; it seems that news literacy as a subset of media literacy would inevitably be associated with the same factors as its parent concept.

**Response**: I hope my response above clears this confusion.

3) Document preparation notes

**Response**: I have corrected the issues listed below.

- page 3 line 9, punctuation
- page 3 line 15, unclear pronoun reference (them)
- page 14 line 2, stray comma, advice -> advise
- page 28 line 7 (headiness) – I have revised this statement. See highlight on page 28.
- some generic statements (e.g., "Notable findings emerged." and "Because hardly any research has examined this angle, let alone in the context of clickbait detection, this study uniquely adds to knowledge.") – I have revised this statement. See highlight on page 4.

Reviewer #8: I believe that the author has adequately addressed mine and the other reviewers’ comments and that the manuscript is now suitable for publication.
