# Supplementary material for: SHOCKING! College professor reveals hidden secrets of news headlines. Can you guess? Examining the efficacy of clickbait detection training and news literacy
Source: PLoS One. 2026 Nov 5;21(11):e0359401. doi: 10.1371/journal.pone.0359401 (PMC13739131; doi:10.1371/journal.pone.0359401)
Supplement: Supplementary file 6 [file pone.0359401.s007.docx]

**Comments to the Author**

1. If the authors have adequately addressed your comments raised in a previous round of review and you feel that this manuscript is now acceptable for publication, you may indicate that here to bypass the “Comments to the Author” section, enter your conflict of interest statement in the “Confidential to Editor” section, and submit your "Accept" recommendation.

Reviewer #7: (No Response)

**Response: N/A**

2. Is the manuscript technically sound, and do the data support the conclusions?

The manuscript must describe a technically sound piece of scientific research with data that supports the conclusions. Experiments must have been conducted rigorously, with appropriate controls, replication, and sample sizes. The conclusions must be drawn appropriately based on the data presented.

Reviewer #7: Yes

**Response: N/A**

3. Has the statistical analysis been performed appropriately and rigorously?

Reviewer #7: Yes

**Response: N/A**

4. Have the authors made all data underlying the findings in their manuscript fully available?

The [PLOS Data policy](https://nam10.safelinks.protection.outlook.com/?url=http%3A%2F%2Ftrack.editorialmanager.com%2FCL0%2Fhttp%3A%252F%252Fwww.plosone.org%252Fstatic%252Fpolicies.action%2523sharing%2F1%2F010f019f6da861d7-41da0006-e34e-42c9-9c7c-6e131b37e20c-000000%2Fgj4Kbo5InFWE2BQvJo5IjUuNU0OLEPbJ9Yzl4S-0pwk%3D258&data=05%7C02%7Cben.wasike%40utrgv.edu%7C9168c3f7a2b0469b6fd008dee3a179a8%7C990436a687df491c91249afa91f88827%7C0%7C0%7C639198479844018146%7CUnknown%7CTWFpbGZsb3d8eyJFbXB0eU1hcGkiOnRydWUsIlYiOiIwLjAuMDAwMCIsIlAiOiJXaW4zMiIsIkFOIjoiTWFpbCIsIldUIjoyfQ%3D%3D%7C0%7C%7C%7C&sdata=%2BDn4luDsxfxkFeR0xsnubocKuA914MgUyjMjecFSUmk%3D&reserved=0) requires authors to make all data underlying the findings described in their manuscript fully available without restriction, with rare exception (please refer to the Data Availability Statement in the manuscript PDF file). The data should be provided as part of the manuscript or its supporting information, or deposited to a public repository. For example, in addition to summary statistics, the data points behind means, medians and variance measures should be available. If there are restrictions on publicly sharing data—e.g. participant privacy or use of data from a third party—those must be specified.

Reviewer #7: Yes

**Response: N/A**

5. Is the manuscript presented in an intelligible fashion and written in standard English?

PLOS ONE does not copyedit accepted manuscripts, so the language in submitted articles must be clear, correct, and unambiguous. Any typographical or grammatical errors should be corrected at revision, so please note any specific errors here.

Reviewer #7: No

**Response: Please see response below**

6. Review Comments to the Author

Please use the space provided to explain your answers to the questions above. You may also include additional comments for the author, including concerns about dual publication, research ethics, or publication ethics. (Please upload your review as an attachment if it exceeds 20,000 characters)

Reviewer #7: I have only some document preparation notes:

Probably this should have 'One,' 'Two,' and 'Three' for the three negative effects; to me this reads like four items, and jumps from First to Third and then has two un-numbered items:

10 However, research indicates that question-based headlines also
11 elicit three negative effects that traditional headlines do not [4]. First, such headlines elicit
12 perceptions of untrustworthiness, confusion, impropriety, and the lack of credibility and
13 seriousness. They also increase the anticipation that news articles following the headlines will
14 elicit the same negative reactions. Third, question-based clickbait headlines reduce engagement
15 with the news via commenting, sharing, or posting reactions such as likes. Other research shows
16 that stories linked to question-based clickbait headlines also receive less engagement online [27].
17 However, readers are more likely to click on question-based clickbait headlines than traditional
18 headlines with no questions [28] even though other research shows that question-based clickbait
19 headlines reduce a reader’s comprehension of accompanying stories [29].

**Response:** I have rewritten this passage as suggested. See highlight on page 6.

I'm not sure these organizations are news agencies per se? The term typically refers to wire services like the Associated Press:

18 These headlines were published by news agencies such as the BBC,
19 The Sun, and Upworthy.

**Response:** This is a good observation. I have since relaced the phrase “news agency” with “news outlets.” See highlight on page 5.

This text reads as redundant:

"Like with clickbait, valence and affect also make a difference. Here, the valence and context with which a headline is written also matter."

**Response:** I have revised to only use valence. See highlight on pages 7–8.

Stray text:

" This explains why news-literate people are more likely to rate stories that carry real rather than generic photos and those with traditional rather than clickbait headlines as better written, more professional, better organized, and they also perceive them as more credible l [52]. l [52]"

**Response:** I did not understand this comment, but I have rewritten this passage as shown below. In the cited study, quality of writing, professionalism, organization, and credibility were measured as separate parameters.

*This explains why news-literate people are more likely to rate stories that carry real rather than generic photos and those with traditional rather than clickbait headlines as better written, more professional, better organized, and they also perceive them as more credible l [52].*

Typo:
"hypothesis 6 predicts that clickbait raining"

**Response:** Corrected – see highlight on page 12.

7. PLOS authors have the option to publish the peer review history of their article ([what does this mean?](https://nam10.safelinks.protection.outlook.com/?url=https%3A%2F%2Ftrack.editorialmanager.com%2FCL0%2Fhttps%3A%252F%252Fjournals.plos.org%252Fplosone%252Fs%252Feditorial-and-peer-review-process%2523loc-peer-review-history%2F1%2F010f019f6da861d7-41da0006-e34e-42c9-9c7c-6e131b37e20c-000000%2FRS_SO4fktI3PXTYzgBXsYxdpBLRkxgskOE72moqp9MM%3D258&data=05%7C02%7Cben.wasike%40utrgv.edu%7C9168c3f7a2b0469b6fd008dee3a179a8%7C990436a687df491c91249afa91f88827%7C0%7C0%7C639198479844041679%7CUnknown%7CTWFpbGZsb3d8eyJFbXB0eU1hcGkiOnRydWUsIlYiOiIwLjAuMDAwMCIsIlAiOiJXaW4zMiIsIkFOIjoiTWFpbCIsIldUIjoyfQ%3D%3D%7C0%7C%7C%7C&sdata=I%2B%2FZOIIWVbA2lTk6J9D4bNdt3b7EqBxtqQWGiw%2FEcHg%3D&reserved=0)). If published, this will include your full peer review and any attached files.


If you choose “no”, your identity will remain anonymous but your review may still be made public.


**Do you want your identity to be public for this peer review?** For information about this choice, including consent withdrawal, please see our [Privacy Policy](https://nam10.safelinks.protection.outlook.com/?url=https%3A%2F%2Ftrack.editorialmanager.com%2FCL0%2Fhttps%3A%252F%252Fwww.plos.org%252Fprivacy-policy%2F1%2F010f019f6da861d7-41da0006-e34e-42c9-9c7c-6e131b37e20c-000000%2FebxFRP79ELSfDHajBkJCxSxbdxwZ0OgwjQjp2XOzT7I%3D258&data=05%7C02%7Cben.wasike%40utrgv.edu%7C9168c3f7a2b0469b6fd008dee3a179a8%7C990436a687df491c91249afa91f88827%7C0%7C0%7C639198479844065392%7CUnknown%7CTWFpbGZsb3d8eyJFbXB0eU1hcGkiOnRydWUsIlYiOiIwLjAuMDAwMCIsIlAiOiJXaW4zMiIsIkFOIjoiTWFpbCIsIldUIjoyfQ%3D%3D%7C0%7C%7C%7C&sdata=X9KkMB0stFrO9wC9f0WWIq6HuD%2F%2FZRLXxl5P3KOIGD4%3D&reserved=0).

Reviewer #7: No

**Response: N/A**
