# Supplementary material for: SHOCKING! College professor reveals hidden secrets of news headlines. Can you guess? Examining the efficacy of clickbait detection training and news literacy
Source: PLoS One. 2026 Nov 5;21(11):e0359401. doi: 10.1371/journal.pone.0359401 (PMC13739131; doi:10.1371/journal.pone.0359401)
Supplement: Supplementary file 7 [file pone.0359401.s008.docx]

**Comments to the Author**

1. If the authors have adequately addressed your comments raised in a previous round of review and you feel that this manuscript is now acceptable for publication, you may indicate that here to bypass the “Comments to the Author” section, enter your conflict of interest statement in the “Confidential to Editor” section, and submit your "Accept" recommendation.

Reviewer #7: (No Response)

**Response: N/A**

2. Is the manuscript technically sound, and do the data support the conclusions?

The manuscript must describe a technically sound piece of scientific research with data that supports the conclusions. Experiments must have been conducted rigorously, with appropriate controls, replication, and sample sizes. The conclusions must be drawn appropriately based on the data presented.

Reviewer #7: Yes

**Response: N/A**

3. Has the statistical analysis been performed appropriately and rigorously?

Reviewer #7: Yes

**Response: N/A**

4. Have the authors made all data underlying the findings in their manuscript fully available?

The [PLOS Data policy](https://nam10.safelinks.protection.outlook.com/?url=http%3A%2F%2Ftrack.editorialmanager.com%2FCL0%2Fhttp%3A%252F%252Fwww.plosone.org%252Fstatic%252Fpolicies.action%2523sharing%2F1%2F010f01a07eb3e7e0-3e616568-4cec-4337-9dd4-8b7375e28a0c-000000%2FAURNXIQZDxb6Af42rxXEDPO0mNeoHf0e4TKZY-gWICI%3D258&data=05%7C02%7Cben.wasike%40utrgv.edu%7C56e97e11908049a6af9008df0d4b4baf%7C990436a687df491c91249afa91f88827%7C0%7C0%7C639244289403823423%7CUnknown%7CTWFpbGZsb3d8eyJFbXB0eU1hcGkiOnRydWUsIlYiOiIwLjAuMDAwMCIsIlAiOiJXaW4zMiIsIkFOIjoiTWFpbCIsIldUIjoyfQ%3D%3D%7C0%7C%7C%7C&sdata=Zp123bsWVZFD9LoxM4iRE%2BZu29x9EfKj8mAjjqrNzic%3D&reserved=0) requires authors to make all data underlying the findings described in their manuscript fully available without restriction, with rare exception (please refer to the Data Availability Statement in the manuscript PDF file). The data should be provided as part of the manuscript or its supporting information, or deposited to a public repository. For example, in addition to summary statistics, the data points behind means, medians and variance measures should be available. If there are restrictions on publicly sharing data—e.g. participant privacy or use of data from a third party—those must be specified.

Reviewer #7: Yes

**Response: N/A**

5. Is the manuscript presented in an intelligible fashion and written in standard English?

PLOS ONE does not copyedit accepted manuscripts, so the language in submitted articles must be clear, correct, and unambiguous. Any typographical or grammatical errors should be corrected at revision, so please note any specific errors here.

Reviewer #7: Yes

**Response: N/A**

6. Review Comments to the Author

Please use the space provided to explain your answers to the questions above. You may also include additional comments for the author, including concerns about dual publication, research ethics, or publication ethics. (Please upload your review as an attachment if it exceeds 20,000 characters)

Reviewer #7: All of my comments from the prior review have been handled with the exception of one -- I pointed out that the following contains stray text: "credible l [52]". The response received was that my comment was not understood. I am not sure how else to clarify, but I have no idea what "credible l" followed by a citation can possibly mean if it is not an error or omission of some kind. Apologies, truly, at prolonging the cycle of review. But this error does persist in the present version after the revision.

**Response:** I had not noticed the extra character after the word “credible” in my initial review, hence my confusion. I have since deleted that character. See highlight on page 9 line 15.

7. PLOS authors have the option to publish the peer review history of their article ([what does this mean?](https://nam10.safelinks.protection.outlook.com/?url=https%3A%2F%2Ftrack.editorialmanager.com%2FCL0%2Fhttps%3A%252F%252Fjournals.plos.org%252Fplosone%252Fs%252Feditorial-and-peer-review-process%2523loc-peer-review-history%2F1%2F010f01a07eb3e7e0-3e616568-4cec-4337-9dd4-8b7375e28a0c-000000%2FgA0E1sgG9CKlQuI9snUxf7VfLu8TkwzPposUB6XiQjQ%3D258&data=05%7C02%7Cben.wasike%40utrgv.edu%7C56e97e11908049a6af9008df0d4b4baf%7C990436a687df491c91249afa91f88827%7C0%7C0%7C639244289403843345%7CUnknown%7CTWFpbGZsb3d8eyJFbXB0eU1hcGkiOnRydWUsIlYiOiIwLjAuMDAwMCIsIlAiOiJXaW4zMiIsIkFOIjoiTWFpbCIsIldUIjoyfQ%3D%3D%7C0%7C%7C%7C&sdata=DVI%2Fi8w6qfbhrtwgEc8X5vCjpL88IdJCbVM3XSHuBcM%3D&reserved=0)). If published, this will include your full peer review and any attached files.


If you choose “no”, your identity will remain anonymous but your review may still be made public.


**Do you want your identity to be public for this peer review?** For information about this choice, including consent withdrawal, please see our [Privacy Policy](https://nam10.safelinks.protection.outlook.com/?url=https%3A%2F%2Ftrack.editorialmanager.com%2FCL0%2Fhttps%3A%252F%252Fwww.plos.org%252Fprivacy-policy%2F1%2F010f01a07eb3e7e0-3e616568-4cec-4337-9dd4-8b7375e28a0c-000000%2FmscBwvWn2Oz4ROdAZGqyxpXsLZp1Xb0Nl6fquBw1r0k%3D258&data=05%7C02%7Cben.wasike%40utrgv.edu%7C56e97e11908049a6af9008df0d4b4baf%7C990436a687df491c91249afa91f88827%7C0%7C0%7C639244289403863575%7CUnknown%7CTWFpbGZsb3d8eyJFbXB0eU1hcGkiOnRydWUsIlYiOiIwLjAuMDAwMCIsIlAiOiJXaW4zMiIsIkFOIjoiTWFpbCIsIldUIjoyfQ%3D%3D%7C0%7C%7C%7C&sdata=8rr6tUQLFXRiFxZ4hRDaiAOa5yEf%2FDkhnEuN5kqzDsU%3D&reserved=0).

Reviewer #7: No

**Response: N/A**
