# Supplementary material for: SHOCKING! College professor reveals hidden secrets of news headlines. Can you guess? Examining the efficacy of clickbait detection training and news literacy
Source: PLoS One. 2026 Nov 5;21(11):e0359401. doi: 10.1371/journal.pone.0359401 (PMC13739131; doi:10.1371/journal.pone.0359401)
Supplement: Peer review history

# Peer review: Decision Letter 0

**DOI:** 10.1371/journal.pone.0359401.r001

**Authors:** Sergio Consoli

5 Mar 2026

PONE-D-26-01307

SHOCKING! College Professor Reveals Hidden Secrets of News Headlines. Can You Guess?

PLOS One?

Dear Dr. Wasike,

Thank you for submitting your manuscript to PLOS ONE. After careful consideration, we feel that it has merit but does not fully meet PLOS ONE’s publication criteria as it currently stands. Therefore, we invite you to submit a revised version of the manuscript that addresses the points raised during the review process.

Please take particular care to the comments raised by the reviewers, and in particular:

- Strengthen the theoretical framing of the literacy training intervention, grounding its components in recent literature on misinformation and news literacy interventions.
- Address the statistical limitations raised, specifically by including a time x condition interaction model (e.g., mixed ANOVA or ANCOVA) rather than relying solely on within-group pre–post comparisons, and by reporting effect sizes and confidence intervals systematically.
- Clarify whether clicking, reading, and sharing intentions should be treated as distinct constructs or facets of a single engagement dimension, and adjust the analysis accordingly.
- Add demographic and media-use control variables to the regression models, and discuss whether main findings remain robust.
- Provide clearer documentation of how clickbait and non-clickbait headlines were identified and validated, including intercoder reliability and any manipulation checks.
- Include pretest balance checks to confirm successful randomization, and address the apparently higher pre-test values in the experimental condition.

Please submit your revised manuscript by Apr 19 2026 11:59PM. If you will need more time than this to complete your revisions, please reply to this message or contact the journal office at plosone@plos.org. When you're ready to submit your revision, log on to https://www.editorialmanager.com/pone/ and select the 'Submissions Needing Revision' folder to locate your manuscript file.

- A letter that responds to each point raised by the academic editor and reviewer(s). You should upload this letter as a separate file labeled 'Response to Reviewers'.
- A marked-up copy of your manuscript that highlights changes made to the original version. You should upload this as a separate file labeled 'Revised Manuscript with Track Changes'.
- An unmarked version of your revised paper without tracked changes. You should upload this as a separate file labeled 'Manuscript'.

If you would like to make changes to your financial disclosure, please include your updated statement in your cover letter. Guidelines for resubmitting your figure files are available below the reviewer comments at the end of this letter.

If applicable, we recommend that you deposit your laboratory protocols in protocols.io to enhance the reproducibility of your results. Protocols.io assigns your protocol its own identifier (DOI) so that it can be cited independently in the future. For instructions see: https://journals.plos.org/plosone/s/submission-guidelines#loc-laboratory-protocols. Additionally, PLOS ONE offers an option for publishing peer-reviewed Lab Protocol articles, which describe protocols hosted on protocols.io. Read more information on sharing protocols at https://plos.org/protocols?utm_medium=editorial-email&utm_source=authorletters&utm_campaign=protocols.

We look forward to receiving your revised manuscript.

Kind regards,

Sergio Consoli

Academic Editor

PLOS One

Journal requirements:

When submitting your revision, we need you to address these additional requirements.

1. Please ensure that your manuscript meets PLOS ONE's style requirements, including those for file naming. The PLOS ONE style templates can be found at

https://journals.plos.org/plosone/s/file?id=wjVg/PLOSOne_formatting_sample_main_body.pdf and

https://journals.plos.org/plosone/s/file?id=ba62/PLOSOne_formatting_sample_title_authors_affiliations.pdf

2. When completing the data availability statement of the submission form, you indicated that you will make your data available on acceptance. We strongly recommend all authors decide on a data sharing plan before acceptance, as the process can be lengthy and hold up publication timelines. Please note that, though access restrictions are acceptable now, your entire data will need to be made freely accessible if your manuscript is accepted for publication. This policy applies to all data except where public deposition would breach compliance with the protocol approved by your research ethics board. If you are unable to adhere to our open data policy, please kindly revise your statement to explain your reasoning and we will seek the editor's input on an exemption. Please be assured that, once you have provided your new statement, the assessment of your exemption will not hold up the peer review process.

If the reviewer comments include a recommendation to cite specific previously published works, please review and evaluate these publications to determine whether they are relevant and should be cited. There is no requirement to cite these works unless the editor has indicated otherwise.

[Note: HTML markup is below. Please do not edit.]

Reviewers' comments:

Reviewer's Responses to Questions

**Comments to the Author**

1. Is the manuscript technically sound, and do the data support the conclusions?

Reviewer #1: Yes

Reviewer #2: Partly

Reviewer #3: Yes

Reviewer #4: Yes

Reviewer #5: Yes

Reviewer #6: Yes

Reviewer #7: Yes

Reviewer #8: Yes

**********

2. Has the statistical analysis been performed appropriately and rigorously?

Reviewer #1: Yes

Reviewer #2: No

Reviewer #3: No

Reviewer #4: Yes

Reviewer #5: Yes

Reviewer #6: Yes

Reviewer #7: Yes

Reviewer #8: Yes

**********

3. Have the authors made all data underlying the findings in their manuscript fully available?

The PLOS Data policy

Reviewer #1: Yes

Reviewer #2: Yes

Reviewer #3: No

Reviewer #4: Yes

Reviewer #5: Yes

Reviewer #6: Yes

Reviewer #7: Yes

Reviewer #8: Yes

**********

4. Is the manuscript presented in an intelligible fashion and written in standard English?

Reviewer #1: Yes

Reviewer #2: Yes

Reviewer #3: Yes

Reviewer #4: Yes

Reviewer #5: Yes

Reviewer #6: Yes

Reviewer #7: Yes

Reviewer #8: Yes

**********

Reviewer #1: The manuscript “SHOCKING! College Professor Reveals Hidden Secrets of News Headlines. Can You Guess?” (PONE-D-26-01307) investigates the correlates of news clickbait headline detection and interactions and investigates the effects of literacy training intervention using a cross-sectional sample. This is a very interesting and timely study on clickbait headlines. I hope these comments, provided in no particular order, are helpful to the authors:

Overall, the literature is engaged and discussed comprehensively, and multiple factors are examined in explaining clickbait related outcomes. While this is good, more focus can be placed on the theoretical underpinnings of the training intervention. The intervention has multiple components such as information provision and quiz. These can be discussed in relation to recent research on literacy training interventions on related fields such as misinformation and news engagement (Kuru, 2025, Literacy training vs. psychological inoculation?; Amazeen and Krishna, 2024, Refuting misinformation: Examining theoretical underpinnings; Badrinathan, 2021, Educative Interventions to Combat Misinformation). Discussion section can elaborate the theoretical implications further as well by taking into account exact mechanisms or targets the different components in interventions aim for. Relatedly, in the methods section, intervention can be elaborated/described in greater detail.

In the experiment, measurement order of outcome variables is as follows: participants first evaluated whether the headlines they have seen were clickbait or not, and then proceeded to answer the other outcomes (clicking on the headline, reading, or sharing the news story). This initial asking about clickbait can introduce experimenter demand effects for the questions that come after: clicking on the headline, reading, or sharing the news story. If anything, it may suppress effects on these three subsequent outcome variables; but there are significant effects observed on those as well. Hence, this should be carefully discussed, and perhaps some question order related methodological literature can be cited (Rasinski, Lee, and Krishnamurty, 2012, Question order effects).

While this outcome measurement issue is relevant for all respondents, for experimental news literacy training group, extra care was shown to not even use the word “clickbait”: “To prevent priming the subjects, the tutorial did not mention the word clickbait” which is good.

More sampling details such as pseudo response rate, removal of respondents criteria can be helpful.

As far as I understand, survey and experiment come from the same dataset. Where was experimental manipulation placed? Are any survey analyses use measures obtained after the experimental manipulation during the survey? I assume not, since they are referred to as pre-test measures. Still, in such a study, this should be very clearly stated. Anything that comes after experimental manipulation (control and training) should not be used in the initial correlational part of the empirical evidence/results. Relatedly, I also wonder if the measure of news literacy questions might have primed/sensitized respondents to focus on clickbait headlines critically above and beyond the news literacy training in the experimental module. This should be discussed and potentially, it can be used as a moderator. Not a theoretical moderator, but as a methodological test to understand if respondents who were at baseline either weak or strong in news literacy behaved similarly in their uptake of the intervention (clickbait news literacy training).

Was the randomization successful? Pretest balance checks can be helpful (on all relevant demographic and theoretical variables, such as news literacy, reflective thinking etc.). Relatedly, looking at the means in Table 6 and 7, I notice that experimental condition’s pre-test values seem to be higher for some reason. This can be by chance, but it should be tested whether this is significant. If so, these pre-test values should be included as a control variable in an ideally OLS or ANCOVA model when groups are compared directly (when not using the pre-test DV variables)

Was there manipulation check for the experiment? It is my understanding that in both control and training manipulation, participant answered a set of quiz questions and all were shown correct answers. Is the data for whether participants answered correctly or incorrectly available. If so, this can be used as an in-built manipulation check too perhaps.

Reviewer #2: Thanks for the opportunity to review this manuscript. Authors should take note of the following and revise the manuscript.

1.This paper explores the identification of clickbait, yet its title adopts the typical rhetoric of clickbait itself such as "SHOCKING!", "Hidden Secrets", and "Can You Guess?". This style is seriously inconsistent with the rigor of academic research and creates a logical contradiction, which may lead readers to question the seriousness of the study.

Although the author clearly points out in the introduction that such a headline is "deceptive" and inconsistent with the facts, continuing to use this title would mean that the author has personally created a "deceptive" case that will soon be criticized in the main text. This is logically contradictory in academic terms.

2.Some scholars have already discussed the relationship between media literacy and clickbait. It is suggested that the introduction should clearly delineate the conceptual boundaries between news literacy and media literacy, and elucidate how news literacy, as a subset of media literacy, with its unique "news verification skills," provides distinctive explanatory power for clickbait detection, thereby more precisely identifying the research gap.

3.The introduction and literature review sections rely excessively on early literature published over 10 years ago, failing to adequately reflect the latest research advancements in this field from the past ten years

4. The literature review has pointed out that the effect of clickbait is significant in polarized issues, but the research hypotheses did not incorporate news issue type as a moderating variable, leading to a disconnect between the theoretical framework and the literature findings.

5.The selection of experimental materials only considered the temporal dimension to avoid memory bias, but failed to control for or analyze the horizontal domain distribution of news headlines such as polarized issues vs. neutral issues, which may allow domain differences to become potential confounding variables.

6.The research findings currently remain primarily at the level of descriptive statistics, lacking in-depth theoretical interpretation of the relationships between variables. In particular, the influence mechanisms of various dimensions of personal locus on news literacy have not been sufficiently discussed. It is recommended that the authors integrate relevant theoretical frameworks to thoroughly analyze the logical implications and theoretical contributions behind these findings.

Reviewer #3: The paper addresses an important and timely question regarding news literacy, cognitive dispositions, and engagement with clickbait content. However, in its current form, the manuscript requires major revisions before considering its publication.

1, The experimental results rely primarily on within-group pre–post comparisons. However, the key inferential question is whether the change over time differs between the intervention and control groups.

Without directly testing the time × condition interaction, it is not possible to conclude that the training had a distinct causal effect. The manuscript should therefore include an appropriate interaction model (e.g., mixed ANOVA or regression with interaction term) and report effect sizes and confidence intervals.

2, Please provide a link to state that the referred data is publicly available for the readers.

3, Clicking, reading, and sharing intentions are extremely highly correlated. Treating them as separate dependent variables in multiple regressions may inflate statistical significance and obscure whether they reflect a single underlying engagement construct. The authors should clarify whether these are theoretically distinct constructs or facets of a broader engagement dimension, and adjust the analysis accordingly (e.g., composite index, multivariate modeling, or correction for multiple testing).

4, The regression models include key predictors but appear to omit potentially important demographic and media-use controls (like age, education, ideology, general news use). Please consider including relevant control variables and report whether the main findings remain robust. This would strengthen causal interpretation and reduce concerns about omitted variable bias.

5, The manuscript would benefit from stronger documentation of how clickbait vs. non-clickbait headlines were identified and validated.

Please clarify:

-Whether independent coders classified the headlines and reported intercoder reliability (if applicable).

-Whether a manipulation check or pilot test confirmed that participants perceived the headlines as clickbait.

-How ecological validity was considered (e.g., absence of images, platform cues).

-Including headline examples in an appendix would improve transparency.

6, The scoring procedure for the signal detection task should be described more clearly and consistently.

7, Please consistently distinguish between survey (pre-test) analyses and experimental findings.

8, Report effect sizes and confidence intervals more systematically.

9, Polishing the clarification of statistical reporting would improve readability.

Reviewer #4: This is a very relevant topic for the times we live in. The paper is written well and the research is clearly embedded in literature and discussed thoroughly.

I have only a couple of minor comments (see attached file).

Reviewer #5: Overall, the design of the experiment and survey is sound and built on previous work in this area. The application of media literacy to news literacy and the underlying psychometric measures are well-defined, and the reasoning for the statistical analysis of OLS regression is sound. I greatly appreciated the thorough explanations of the variables in the background information and examples of questions from the surveys. The only revisions needed are to include the tools used to do the analysis (SPSS, R?), some awkward descriptions of variables on pg.6 12-16 (emotionally charged words and engagement), and a discussion of the analysis of demographic information. The author points to the care of producing a sample that reflects the diversity of the larger population, but does not include how the factors of gender, ethnicity, race, age, etc may interact with the other variables to predict news literacy and clickbait detection. If these factors offer no predictability to the model, then that should be stated.

Reviewer #6: Dear authors,

I was very pleased to review your manuscript. This is a well-written manuscript that I reviewed so far this year. The paper provided sound background indicating gaps in the literature and its broad theoretical implications. The most important aspect of the manuscript it's the combination of the survey and experimental design. Relevant analyses were conducted to support the hypotheses. The discussion of the results was adequate. The overall quality of the writing is good and simple. After back and forth examination of the paper, I found no substantial issues that would prevent me from recommending it in its current form. Thus, I opt for an "ACCEPT" decision.

It was nice reading your work.

Regards

Reviewer #7: See attached review. This box has a minimum length, so I include the initial paragraph but ask that the full review be considered.

The paper presents the results of a survey and experimental intervention into 'clickbait engagement'. The study reports evidence in line with most of the current literature, but develops this evidence through an experimental framework to extend our confidence in the current understanding. The paper drew my immediate curiosity as to the state of objective measures of clickbait. But I am uncertain about the state of agreement as to these definitions -- it seems likely that one person's clickbait is another person's viral content; are there agreed-upon thresholds about the difference between clickbait and good marketing? I can see deceptiveness as an area where there would be more agreement. How does one distinguish between a headline with a question that is also clickbait, and one that isn't? In general this line of inquiry may be quite settled in the area of clickbait studies, but I think for a PLOS audience it would be helpful to take a slightly more expository approach. In particular I was not sure how to think about non-deceptive clickbait, and I notice that the Instrument in the appendix does not seem to cover this category and the text unfortunately contradicts itself on this concept. I also had trouble with the way the concept of personal locus is explained throughout; more guidance would be helpful here.

This is strong work but would benefit from revision, particularly with respect to how it engages the concepts under study. A clearer conceptual framework and the relationship of constructs to one another would allow this work to be placed more firmly in the literature. I organize my review to match the ordering of the work, noting various minor points at the end.

Reviewer #8: Thank you for the opportunity to read this mansucript. I really enjoyed it, and I think it would make a great candidate for inclusion as a PLOS one article. I do have some minor comments that I believe will make the manuscript stronger prior to publication, but that amount to curiosity and a few minor details that may reflect the framing/writing of the text rather than a weakness in the work itself. Comments below, presented in no particular order:

- I'm interested in seeing a bit more discussion of the demographic makeup of your sample. You mention that your total sample is representative of the US population, but does this mean that both your test group and your control group were representative samples? Were the percentage of respondents in each demographic category the same, or were there differences? This isn't clear in the text, so if both groups were representative samples, I would suggest making that a bit clearer in the text.

- if it is the case that your two groups were comprised of different demographic makeups, I'm curious how that might influence your results, since there isn't very much discussion of demographics in the manuscript. Specifically, I'm wondering about ideological differences in news literacy and vulnerability to clickbait, since there's ample research that deals with formation of political echo chambers and political misinformation. if there were notable demographic differences on your literacy or other metrics, perhaps this is something that could be discussed in a short appendix.

- your introduction is at times a bit repetitive, such as for example in the second paragraph of your "effects of clickbait" section, where you mention the definition of forward-referencing despite already describing what forward referencing is in your opening paragraph. I would suggest tightening up the writing a bit here and make sure you aren't repeating yourself, but this is a very minor thing

All in all, I think this is a strong manuscript that would make a great candidate for publication.

**********

what does this mean?). If published, this will include your full peer review and any attached files.

If you choose “no”, your identity will remain anonymous but your review may still be made public.

**Do you want your identity to be public for this peer review?**  For information about this choice, including consent withdrawal, please see our Privacy Policy

Reviewer #1: No

Reviewer #2: No

Reviewer #3: No

Reviewer #4: No

Reviewer #5: **Yes:**  Denise D Coberley

Reviewer #6: No

Reviewer #7: No

Reviewer #8: No

**********

[NOTE: If reviewer comments were submitted as an attachment file, they will be attached to this email and accessible via the submission site. Please log into your account, locate the manuscript record, and check for the action link "View Attachments". If this link does not appear, there are no attachment files.]

To ensure your figures meet our technical requirements, please review our figure guidelines: https://journals.plos.org/plosone/s/figures

You may also use PLOS’s free figure tool, NAAS, to help you prepare publication quality figures: https://journals.plos.org/plosone/s/figures#loc-tools-for-figure-preparation.

NAAS will assess whether your figures meet our technical requirements by comparing each figure against our figure specifications.

Attachment Submitted filename: comments.docx

Attachment Submitted filename: PLoS One - clickbait study.pdf

# Author comment: Author response to Decision Letter 1

**DOI:** 10.1371/journal.pone.0359401.r002

1 Apr 2026

I have also attached my response to the reviewers as a separate file

Reviewer #1

Reviewer #1: The manuscript “SHOCKING! College Professor Reveals Hidden Secrets of News Headlines. Can You Guess?” (PONE-D-26-01307) investigates the correlates of news clickbait headline detection and interactions and investigates the effects of literacy training intervention using a cross-sectional sample. This is a very interesting and timely study on clickbait headlines. I hope these comments, provided in no particular order, are helpful to the authors:

Overall, the literature is engaged and discussed comprehensively, and multiple factors are examined in explaining clickbait related outcomes. While this is good, more focus can be placed on the theoretical underpinnings of the training intervention. The intervention has multiple components such as information provision and quiz. These can be discussed in relation to recent research on literacy training interventions on related fields such as misinformation and news engagement (Kuru, 2025, Literacy training vs. psychological inoculation?; Amazeen and Krishna, 2024, Refuting misinformation: Examining theoretical underpinnings; Badrinathan, 2021, Educative Interventions to Combat Misinformation). Discussion section can elaborate the theoretical implications further as well by taking into account exact mechanisms or targets the different components in interventions aim for.

Response: I have incorporated the suggested literature both within the front matter and in the discussion. In the front matter, I tie inoculation training to news literacy training – se highlight on page 9 (lines 1-3). In the discussion, I expound on how this study opens a venue to study inoculation and how both inoculation training and clickbait training offer practical uses – see highlight on page 29 (lines 1-2).

Relatedly, in the methods section, intervention can be elaborated/described in greater detail.

Response: I have revised this section to elaborate on the stimuli, headline selection, and procedure. See highlight on page 14 (lines 16–21) and page 15 (linesn1–24).

In the experiment, measurement order of outcome variables is as follows: participants first evaluated whether the headlines they have seen were clickbait or not, and then proceeded to answer the other outcomes (clicking on the headline, reading, or sharing the news story). This initial asking about clickbait can introduce experimenter demand effects for the questions that come after: clicking on the headline, reading, or sharing the news story. If anything, it may suppress effects on these three subsequent outcome variables; but there are significant effects observed on those as well. Hence, this should be carefully discussed, and perhaps some question order related methodological literature can be cited (Rasinski, Lee, and Krishnamurty, 2012, Question order effects).

Response: This is a legitimate concern that I had not addressed earlier. When designing the survey, I used blocks for each headline and also for the survey questions. Therefore, the subjects viewed the clickbait and non-clickbait headlines randomly – I mention this in the procedure section (. What I did not mention is that they also viewed the survey questions randomly, so no one subject viewed headlines and questions in the same order. I have since addresses this issue in the limitations section – see highlight on page 31 (lines 1–8).

While this outcome measurement issue is relevant for all respondents, for experimental news literacy training group, extra care was shown to not even use the word “clickbait”: “To prevent priming the subjects, the tutorial did not mention the word clickbait” which is good.

Response: Not applicable

More sampling details such as pseudo response rate, removal of respondents criteria can be helpful.

Response: The Qualtrics panel that I used had a 100% completion rate. Only those who failed the attention check questions were eliminated. See highlight on page 12 (lines 15–16).

As far as I understand, survey and experiment come from the same dataset. Where was experimental manipulation placed?

Response: I had not discussed this issue before I do. The in-study quiz during the main experiment acted as the manipulation check. see highlight on page 19 (lines 5–13).

Are any survey analyses use measures obtained after the experimental manipulation during the survey? I assume not, since they are referred to as pre-test measures. Still, in such a study, this should be very clearly stated. Anything that comes after experimental manipulation (control and training) should not be used in the initial correlational part of the empirical evidence/results.

Response: No, none of the survey-based variables were tested or measured after exposure. I’ve tried to say this in various places but each time it ended up reading very awkwardly. Therefore, I added a sentence at the beginning of the results section – See highlight on page 19 (line 18): Hypotheses 1–5 were tested based only on data collected in the pre-test.

Relatedly, I also wonder if the measure of news literacy questions might have primed/sensitized respondents to focus on clickbait headlines critically above and beyond the news literacy training in the experimental module. This should be discussed and potentially, it can be used as a moderator. Not a theoretical moderator, but as a methodological test to understand if respondents who were at baseline either weak or strong in news literacy behaved similarly in their uptake of the intervention (clickbait news literacy training).

Response: I get the gist of the concern, but I do not fully understand what I need to do or how to it. Did the reviewer mean to say clickbait training rather than news literacy training? If referring to the news literacy questions, these are very general and they query about broad aspects of the media industry, and not about specific such as how headlines are written. In fact, the only question that comes close to that refers to the news lead. It is unlikely that the news literacy questions may prime subjects to be critical of the media more than they are because research suggests that news iterate people are better at analyzing headlines by default. Additionally, subjects were never informed if they got the news literacy survey questions right or wrong.

If referring to the clickbait training, running it as a moderator would only apply to half the sample, those who were exposed to this intervention.

Was the randomization successful? Pretest balance checks can be helpful (on all relevant demographic and theoretical variables, such as news literacy, reflective thinking etc.).

Response: I ran these statistics and there were no significant difference between the test and control group in any of the survey-related variables. See highlight on page 13 (lines 5–8) and supplemental tables 1 and 2, where I compared the two groups regarding demographics, and here too there were no differences.

Relatedly, looking at the means in Table 6 and 7, I notice that experimental condition’s pre-test values seem to be higher for some reason. This can be by chance, but it should be tested whether this is significant. If so, these pre-test values should be included as a control variable in an ideally OLS or ANCOVA model when groups are compared directly (when not using the pre-test DV variables).

Response: In my revision, I use ANOVA to analyze the pre- and post-test detection and engagement variables.

Was there manipulation check for the experiment? It is my understanding that in both control and training manipulation, participant answered a set of quiz questions and all were shown correct answers. Is the data for whether participants answered correctly or incorrectly available. If so, this can be used as an in-built manipulation check too perhaps.

Response: yes, the in-study quiz was the manipulation check. See highlight on page 19 (lines 5–13).

-------------

Reviewer #2

Reviewer #2: Thanks for the opportunity to review this manuscript. Authors should take note of the following and revise the manuscript.

1.This paper explores the identification of clickbait, yet its title adopts the typical rhetoric of clickbait itself such as "SHOCKING!", "Hidden Secrets", and "Can You Guess?". This style is seriously inconsistent with the rigor of academic research and creates a logical contradiction, which may lead readers to question the seriousness of the study.

Although the author clearly points out in the introduction that such a headline is "deceptive" and inconsistent with the facts, continuing to use this title would mean that the author has personally created a "deceptive" case that will soon be criticized in the main text. This is logically contradictory in academic terms.

Response: It’s more strategic than contradictory. I chose this title strategically to segue into the introduction and have the reader first view a detailed definition of clickbait, one that also gives examples of forward-references, emotionally charged words, and question headlines. This is something I pondered lengthily. Also, the title is not deceptive and reflects plenty of titles used in clickbait studies:

• Jung AK, Stieglitz S, Kissmer T, Mirbabaie M, Kroll T (2022) Click me…! The influence of clickbait on user engagement in social media and the role of digital nudging. PLOS ONE 17(6): e0266743. https://doi.org/10.1371/journal.pone.0266743

• Scott, Kate. “You Won’t Believe What’s in This Paper! Clickbait, Relevance and the Curiosity Gap.” Journal of Pragmatics [Amsterdam], vol. 175, April 2021, pp. 53–66, https://doi.org/10.1016/j.pragma.2020.12.023.

• Béna J, Rouard M, Corneille O. You won't believe it! Truth judgments for clickbait headlines benefit (but less so) from prior exposure. Applied cognitive psychology. 2023;37(6):1418–29.

• Shrestha A, Behfar A, Al-Ameen MN. “It is luring you to click on the link with false advertising” - mental models of clickbait and its impact on user’s perceptions and behavior towards clickbait warnings. International Journal of Human–Computer Interaction. 2024;41(4):2352–70.

• ‘I Urge You To See This...’. Clickbait As One Of The Dominant Features Of

• Contemporary Online Headlines.

• Mukherjee P, Dutta S, De Bruyn A. Did clickbait crack the code on virality? Journal of the Academy of Marketing Science. 2022;50(3):482–502.

2.Some scholars have already discussed the relationship between media literacy and clickbait. It is suggested that the introduction should clearly delineate the conceptual boundaries between news literacy and media literacy, and elucidate how news literacy, as a subset of media literacy, with its unique "news verification skills," provides distinctive explanatory power for clickbait detection, thereby more precisely identifying the research gap.

Research: I have addressed this issue by emphasizing the association between news literacy and media literacy generally, but also specifying the association between news literacy and the four media literacy variables. See highlight on page 3 (line 23–24) and page 4 (lines 1–5).

3.The introduction and literature review sections rely excessively on early literature published over 10 years ago, failing to adequately reflect the latest research advancements in this field from the past ten years

Response: Most of the dated references were in the introduction and the Effects of Clickbait sections. I have either removed some of the old references and replaced them with newer sources or added newer references to the old ones. The few dated references I left were either important survey data or seminal studies such as Blom and Hansen (2015) and Shostak and Gillespie (2014). I have incorporated the following:

• Li X, Zhou J, Xiang H, Cao J. Attention grabbing through forward reference: an ERP study on clickbait and top news stories. International Journal Of Human-Computer Interaction. 2024;40(11):3014–29.

• Enache A, Militaru M, Ioncica D. The Emotional Appeal of Clickbait Headlines – between Entertainment and Deception. Dialogos. 2022;

• Su Y, Lee DKL, Xiao X. "I enjoy thinking critically, and I'm in control": Examining the influences of media literacy factors on misperceptions amidst the COVID-19 infodemic. Comput Human Behav. 2022 Mar;128:107111. doi: 10.1016/j.chb.2021.107111. Epub 2021 Nov 27. PMID: 34866771; PMCID: PMC8631744.

• Shrestha A, Flood A, Sohrawardi S, Wright M, Al-Ameen MN. A First Look into Targeted Clickbait and its Countermeasures: The Power of Storytelling. Proceedings of the 2024 CHI Conference on Human Factors in Computing Systems; Honolulu, HI, USA: Association for Computing Machinery; 2024. p. Article 971.

• Sundar SS, Snyder EC, Liao M, Yin J, Wang J, Chi G. Sharing without clicking on news in social media. Nature Human Behaviour. 2025;9(1):156–68.

4. The literature review has pointed out that the effect of clickbait is significant in polarized issues, but the research hypotheses did not incorporate news issue type as a moderating variable, leading to a disconnect between the theoretical framework and the literature findings.

Response: This is a good observation, but the type of news that the clickbait headline accompanied was not within the focus of the study. I have mentioned this in the limitations section. See highlight on pages 30 (lines 20–23).

5.The selection of experimental materials only considered the temporal dimension to avoid memory bias, but failed to control for or analyze the horizontal domain distribution of news headlines such as polarized issues vs. neutral issues, which may allow domain differences to become potential confounding variables.

Response: This too is a good observation, but the valence of the clickbait headline was not within the focus of the study. Like with the news types or issues, have mentioned this in the limitations section. See highlight on page 30 (lines 20–23).

6.The research findings currently remain primarily at the level of descriptive statistics, lacking in-depth theoretical interpretation of the relationships between variables. In particular, the influence mechanisms of various dimensions of personal locus on news literacy have not been sufficiently discussed. It is recommended that the authors integrate relevant theoretical frameworks to thoroughly analyze the logical implications and theoretical contributions behind these findings.

Response: This reflects the writing style in my field, where we present results in an objective manner and then contextualize them in the discussion section. While I had done this before, I have now expanded my discussion on the confluence among news literacy and the personal locus variables See highlight on page 26 (lines 12–23).

-----------

Reviewer #3

Reviewer #3: The paper addresses an important and timely question regarding news literacy, cognitive dispositions, and engagement with clickbait content. However, in its current form, the manuscript requires major revisions before considering its publication.

1, The experimental results rely primarily on within-group pre–post comparisons. However, the key inferential question is whether the change over time differs between the intervention and control groups.

Without directly testing the time × condition interaction, it is not possible to conclude that the training had a distinct causal effect. The manuscript should therefore include an appropriate interaction model (e.g., mixed ANOVA or regression with interaction term) and report effect sizes and confidence intervals.

Response: I have since rewritten my description of the experiment to clarify that it was a single factor between subjects design. The single factor was clickbait training with two levels (clickbait training vs. placebo training). See highlight on page 12 (lines 7-12).

Response: To address the time issue, I have now included results from ANOVA analysis that used mean differences between the pre- and post-test scores and a dichotomous variable for test group vs. control group a done in previous studies single factor, two-level studies.. See explanation on page 24 (lines 16–20).

2, Please provide a link to state that the referred data is publicly available for the readers.

Response: I had already provided this statement and a link to the data during the submission process form even though it does not appear in this version of the manuscript. I think the publisher will include the link once the paper in available online.

3, Clicking, reading, and sharing intentions are extremely highly correlated. Treating them as separate dependent variables in multiple regressions may inflate statistical significance and obscure whether they reflect a single underlying engagement construct. The authors should clarify whether these are theoretically distinct constructs or facets of a broader engagement dimension, and adjust the analysis accordingly (e.g., composite index, multivariate modeling, or correction for multiple testing).

Response: I have since combined these three into a composite engagement variable. For the sake of nuance, I have included data on the three separate parameters separately as supplemental tables.

4, The regression models include key predictors but appear to omit potentially important demographic and media-use controls (like age, education, ideology, general news use). Please consider including relevant control variables and report whether the main findings remain robust. This would strengthen causal interpretation and reduce concerns about omitted variable bias.

Response: I have redone the regression and included age, income, and ideology. The survey did no collect education data because the study did not predict this variable. I have also included a discussion on the effect of these demographic variables in the discussion section. See highlight on page 28 (lines 4–16).

5, The manuscript would benefit from stronger documentation of how clickbait vs. non-clickbait headlines were identified and validated.

Please clarify:

-Whether independent coders classified the headlines and reported intercoder reliability (if applicable).

-Whether a manipulation check or pilot test confirmed that participants perceived the headlines as clickbait.

-How ecological validity was considered (e.g., absence of images, platform cues).

-Including headline examples in an appendix would improve transparency.

Response: As I mention in the method section, I used clickbait headlines from a previous study (see below) whose authors created them from scratch and validated them in a small sample pilot test. For my study, I randomly picked real headlines and tested them in a pilot study similar to the Béna, et al. approach. I have now included a more detailed description of this process. See Headline Selection section on page 14 (lines 2–14)..

Béna J, Rouard M, Corneille O. You won't believe it! Truth judgments for clickbait headlines benefit (but less so) from prior exposure. Applied cognitive psychology. 2023;37(6):1418–29.

6, The scoring procedure for the signal detection task should be described more clearly and consistently.

Response: I have revised this section for clarity. See highlight on page 17 (lines 5-7).

7, Please consistently distinguish between survey (pre-test) analyses and experimental findings.

Response: I have included statements to differentiate between the two on page 19 (line 18) and on page 23 (lines 13–14).

8, Report effect sizes and confidence intervals more systematically.

Response: I have included the effects sizes and confidence intervals when applicable. See tables 2 and 3 and supplemental tables 3–5 for confidence intervals. Supplemental tables 6–7) show effects sizes for t-test and ANOVA results.

9, Polishing the clarification of statistical reporting would improve readability.

Response: Done

------------

Reviewer #4

Reviewer #4: This is a very relevant topic for the times we live in. The paper is written well and the research is clearly embedded in literature and discussed thoroughly.

I have only a couple of minor comments (see attached file).

-----------

The purpose of the review is to provide the editors with an expert opinion regarding the validity and quality of the manuscript under consideration. The review should also supply authors with explicit feedback on how to improve their papers so that they will be acceptable for publication in PLOS One. As you write, consider the following points:

• What are the main claims of the paper and how significant are they for the discipline?

o The study examines the effects of news literacy and a clickbait training on clickbait detection and clickbait behavior. The author found that readers who are news literate are less likely to engage in clickbait behavior. Additionally, a brief training seemed to improve clickbait detection and decrease clicking. It seems that the American public (based on the sample used) is not very news literate. The subject of this paper is

certainly relevant in today’s climate of polarization, where accusations of fake news are

being used widely and wildly to discredit and silence.

Response: Not applicable

• Are the claims properly placed in the context of the previous literature? Have the authors treated the literature fairly?

o The study is embedded in the context of previous literature well.

Response: Not applicable

• Do the data and analyses fully support the claims? If not, what other evidence is required?

o The data and analyses show significant relationships between literacy and clickbait detection. The brief training showed improvement in detecting clickbait. The author does draw causation with data that only shows correlation (discussion, line 7 of page 25), which needs to be revised. The statement “news literacy…directly improved detection” is not correct based on the analyses. Correlation does not equal causation…

Response: This statement refers to news literacy and not clickbait detection training. It also refers to the pre-rest data. Even then, the reviewer is correct because as written, the statement does not explicitly point out that this conclusion is based on both the correlation and regression analysis results In fact, news literacy was correlated with clickbait detection (r = 29, p < .001) but it also had a main effect on detection in the pre-test (� = .22, p < .01). I have revised it to mention the main effects from the regression analysis. See highlight on page 19 (lines 22–24)..

• Are details of the methodology sufficient to allow the experiments to be reproduced?

o Yes. As a minor point, Table 1 is not formatted correctly; the M and SD columns belong on the right-hand side.

Response: I have revised the table.

• Is any software created by the authors freely available?

o N/A

• Is the manuscript well organized and written clearly enough to be accessible to non-specialists?

o Yes. The paper is written very clearly.

• Is it your opinion that this manuscript contains an NIH-defined experiment of Dual Use concern?

Although confidential comments to the editors are respected, any remarks that might help to strengthen the paper should be directed to the authors themselves.

Reviewer #5

Reviewer #5: Overall, the design of the experiment and survey is sound and built on previous work in this area. The application of media literacy to news literacy and the underlying psychometric measures are well-defined, and the reasoning for the statistical analysis of OLS regression is sound. I greatly appreciated the thorough explanations of the variables in the background information and examples of questions from the surveys.

The only revisions needed are to include the tools used to do the analysis (SPSS, R?),

Response: I used SPSS, and I have now included a statement on that. See highlight on page 19 (Line 13).

Some awkward descriptions of variables on pg.6 12-16 (emotionally charged words and engagement),

Response: I have revised this section. See highlight on page 6 (lines 16_22).

and a discussion of the analysis of demographic information.

Response: I have done this in several ways:

• Because I now include the demographic variables I the regression analysis, I have added a section discussing their impact on the dependent variables. See last paragraph of the discussion section, page 29 (lines 4–16).

• I have also incorporated a new comparison of demographics between the test and control groups. See supplemental tale 1. I also contextualize this comparisons on page 13 (lines 5-9)

The author points to the care of producing a sample that reflects the diversity of the larger population, but does not include how the factors of gender, ethnicity, race, age, etc may interact with the other variables to predict news literacy and clickbait detection. If these factors offer no predictability to the model, then that should be stated.

Response: I have now included these demographics variables in the regression analysis. See tables 1 and 2 and supplemental tables 3–5.

-------------

Reviewer #6

Reviewer #6: Dear authors,

I was very pleased to review your manuscript. This is a well-written manuscript that I reviewed so far this year. The paper provided sound background indicating gaps in the literature and its broad theoretical implications. The most important aspect of the manuscript it's the combination of the survey and experimental design. Relevant analyses were conducted to support the hypotheses. The discussion of the results was adequate. The overall quality of the writing is good and simple. After back and forth examination of the paper, I found no substantial issues that would prevent me from recommending it in its current form. Thus, I opt for an "ACCEPT" decision.

It was nice reading your work.

Regards

Response: Not applicable

------------

Reviewer #7

Reviewer #7: See attached review. This box has a minimum length, so I include the initial paragraph but ask that the full review be considered.

The paper presents the results of a survey and experimental intervention into 'clickbait engagement'. The study reports evidence in line with most of the current literature, but develops this evidence through an experimental framework to extend our confidence in the current understanding. The paper drew my immediate curiosity as to the state of objective measures of clickbait. But I am uncertain about the state of agreement as to these definitions -- it seems likely that one person's clickbait is another person's viral content; are there agreed-upon thresholds about the difference between clickbait and good marketing? I can see deceptiveness as an area where there would be more agreement. How does one distinguish between a headline with a question that is also clickbait, and one that isn't? In general this line of inquiry may be quite settled in the area of clickbait studies, but I think for a PLOS audience it would be helpful to take a slightly more expository approach. In particular I was not sure how to think about non-deceptive clickbait, and I notice that the Instrument in the appendix does not seem to cover this category and the text unfortunately contradicts itself on this concept. I also had trouble with the way the concept of personal locus is explained throughout; more guidance would be helpful here.

This is strong work but would benefit from revision, particularly with respect to how it engages the concepts under study. A clearer conceptual framework and the relationship of constructs to one another would allow this work to be placed more firmly in the literature. I organize my review to match the ordering of the work, noting various minor points at the end.

----------

Abstract and Introduction: .......................

The work describes personal locus as "a competency adjacent to news literacy," without saying what personal locus actually is. Given how important this concept is for the work, I think it's important to really nail this. The Introduction / line 18-19 on page 3 get a bit closer to offering a definition, but I still don't feel like I quite grasp it or know what it means from what's presented here. I did a bit of searching on the definition to ground myself (seemingly, belief that one has control over one's life), but I think it would aid the reader to be supplied with a definition and reference in the first 2 pages since it's a key concept in the work. In general, more clarity about concepts would be helpful in the introduction and elsewhere as noted.

Response: I had defined personal locus, but that was further down in the paper under the News Literacy and Personal Locus section. I have now defined it earlier in the introduction and also rewritten the passage where I defined it earlier to make it more obvious and better tie it to media literacy and news literacy. See highlights on page 3 (lines 23–24) and page 9 (lines 1–6).

The effects of clickbait .......................

I think this next section is related work or background -- standard section headers are likely not required by this venue but do tend to aid the reader.

The extended discussion of clickbait as a phenomenon was interesting to read and resonated with me; it would be useful to expand on this by including some sense of the degree of agreement people have about what is and isn't clickbait and generally establish the validity of the construct.

Response: While there is a consensus on the definition of clickbait and the characteristics of clickbait headlines, declaring a certain headline to be clickbait is subjective and changes from person to person. It is for this reason that I focused my discussion on the characteristics of clickbait and gave examples from seminal studies like Blom & Hansen and Jodłowiec.

Response: Regarding validity, I had not discussed the validity of the clickbait and non-clickbait headlines used here. I have now done so, and this also answers the concerns the reviewer raised in their initial comments. See highlight in method section – pages 14 (lines 1-14).

Additionally, the issue of deceptive clickbait is important, but that was beyond the scope of the current study.

Pages 5 and 6 describe the current state of research about clickbait with respect to numerous measures. Although this material really is interesting, I think at times it veers away from the analytical focus of the current work.

Response: This section serves several purposes. First, it explains the nuances of clickbait beyond the definition and gives the reader a better picture of what clickbait is given the subjectiveness of determine what is and what not clickbait. The examples of forward-references and emotionally charged words help clarify how clickbait headlines are presented. The discussion on the effects of clickbait in this section also ground in theory, the hypotheses that predict the effect of exposure to clickbait.

The role of news headlines .......................

I'm not sure a study demonstrating that a deceptive headline has consequences (page 7 line 14) is evidence that a full reading is required in the case of a non-deceptive headline (line 15).

Response: The study cited here found that people made judgments about the subject of an opinion news story based on a headline that was misleading or incongruent with the story itself. This suggests that a full reading of the story is needed to get the true information. I have changed the wording to that shown below. See highlight on page 7 (lines 15–17).

“For instance, a misleading headline accompanying an opinion article may affect the reader’s judgement about the story’s subject even when that headline is incongruent with the gist of the article.”

I am not sure what "the sentiment and context with which a headline is written" means (page 7 line 17); the lines of text following this statement only seem to talk about sentiment.

Response: Tis refers to the valence and effect of the words, negative or positive. I have replaced the word sentiment with valence and affect. See highlights on page 7 (lines 19–22).

News Literacy and Personal Locus .......................

Here I find I am a little bit confused again about the concepts under study; news literacy is clear enough, but now I see a conflicting conceptual relationship expressed. In line 5 and again on line 12, it sounds as if the definition of personal locus is actually a sub-concept inside of a media literacy framework, but I am finding this difficult to understand as the concept expressed in some literature is quite a bit larger -- it seems to be a more general feature of people, with implications for many things, including their media literacy and news literacy. Perhaps a conceptual diagram along with directly addressing the relationships among concepts would help?

Response: Locus of control is the larger, broader concept. Personal locus was the specific concept coined by Potter (2004) in his discussion of media literacy. To refer to one’s control over their media use. In my revision on page 8 (9–14), I tr to connect these three concepts.

The engagement with Dewey is nice to see here.

It's a matter of taste, but in a complex series of hypotheses (there are 24 of them if you include the letters), my preference as a reader is to have the hypotheses positioned within the literature review, such that there is a direct bright line between "Literature says..." and "H# proposes" without me needing to scroll back and forth in the section.

Alternately the ordering of the concepts in the hypotheses could match up the ordering of the background; for example I see that the lit about detection training is discussed in the middle of the section, but it's the last hypothesis.

Response: I have rearranged the hypotheses as suggested. To allow for a logical discussion of the results, I left H6 as the last one given that H1–H5 predict about the four personal locus variables. It would be confusing to present the clickbait training results in between the results of the personal locus variables.

Method .......................

It would be helpful to point back to the hypotheses by number here in the method and measures section to aid the reader in tracking the argument.

I was not sure why synthetic headlines were used for clickbait and real but old headlines used for non-clickbait; the opening argument of the paper describes the BBC as having clickbait so presumably so might the NYT or AP. Presumably either or both could be used, but using a different procedure for one versus the other is unclear to me.

Response: Ia have provided a more detailed explanation of how and why the headlines were collected. See the Headline Selection section in the methodology – pages 14 (lines 1–14).

I was surprised to see the signal vs noise exploration collapsed down to distinguishing advertorials; the background seems to frame this much more broadly (albeit briefly -- I think just lines 8-12 on page 16?

Response: It’s true that signal detection may refer to a broad range of materials. In the literature, I discuss the Tully, et al. study that emphasized that news-literate people need to distinguish between news content from other content like opinions and advertisements. I used advertorials because they deceptively mimic editorial content and research shows that this make them more engaging, suggesting that it takes a higher level of signal detection ability to distinguish them from traditional advertisement content. I have added this explanation in the method section. See highlight on page 17 (lines 5–7).

Results ................

It would be useful to have more interpretation of the empirical results -- particularly the practical significance of the statistically significant factors. Some visualizations showing relative effects at a glance would be useful as well; otherwise one has to scroll back and forth to weigh out these factors.

Response: Reviewer 2 also brought up this issue. I explained that in my filed, we present the results in an objective manner and contextualize and interpret them in the discussion section. I have since expanded my previous discussion of the significant and non-significant results. See highlights on pages 24–25).

Discussion ................

I would like to see more exploration here of the contradicted / failed hypotheses -- what might be the implications of this reversal?

Response: I have expanded on potential reasons to explain this failed hypothesis. See highlight on page 27 (lines 11–17). And page 27 (lines 18–23) and page 29 (line 1–4).

Implications and Limitations ................

The phrase "expanding the avenues of scholarly inquiry" is vague as a summary of contribution; presumably every study does this?

Response: I have now specified that it opens avenue to psychology and misinformation susceptibility research. See highlights on page 29 (lines 20–21) and page 30 (lines 4–8).

The section says that "clickbait uses deception" but the opening definition states that it can be both deceptive and non-deceptive?

Response: This was supposed to read – Because some clickbait uses deception. I have made the revision. See highlight on page 30 Line 3).

Document preparation .......................

I saw some unevenness here, with stray / missing punctuation and spacing. I list the items I noted as I read, but the quantity suggests the need for a thorough proofread.

Line 18 / page 3 would be clearer with an Oxford comma (otherwise, it's unclear as to whether this is a list of three measures, or if the work is defining personal locus as "a psychometric measure of media literacy ...and misinformation susceptibility". I was sufficiently confused that I re-read this bit repeatedly -- up until I realized it was simply an ambiguity, rather than a phrase offering an inline definition.

Response: Done

odd punctuation in p. 4 line 14 -- there's an unpaired double quote, and an unpaired parenthesis.

stray punctuation -- line 18 of page 6

stray spaces -- page 7 line 12

p.9, "differentiating between signal from noise" -- unclear use of idiom.

Some missing spaces and an extra comma (p 9, line 14 and 21)

Unclear: "The reflects the self-awareness" (p10. line 10)

Missing period: (p 10 line 18)

"discission" (p10 line 22)

Usually I see a colon at the end of lines like line 22, p 10.

Extra comma, p 15 line 6

Stray letter, p 16 line 3

"Hypotheses 5" (p 23 line 10)

"news literacy write large" (p 25 line 16)

"which exclusively composed" (p 26 line 20)

Response: Done

References .......................

Some formatting errors make this section at times difficult to read (missing italics, missing punctuation, missing publication name).

--------------

Reviewer #8

Reviewer #8: Thank you for the opportunity to read this manuscript. I really enjoyed it, and I think it would make a great candidate for inclusion as a PLOS one article. I do have some minor comments that I believe will make the manuscript stronger prior to publication, but that amount to curiosity and a few minor details that may reflect the framing/writing of the text rather than a weakness in the work itself. Comments below, presented in no particular order:

- I'm interested in seeing a bit more discussion of the demographic makeup of your sample. You mention that your total sample is representative of the US population, but does this mean that both your test group and your control group were representative samples? Were the percentage of respondents in each demographic category the same, or were there differences? This isn't clear in the text, so if both groups were representative samples, I would suggest making that a bit clearer in the text.

Response: I did not design each group to be representative Rather; I recruited one large that was representative and then randomly assigned some to the test group and some to the control group. The randomization was successful because there were no significant differences between the two groups among the demographic variables and among the survey-related predictor variables. I have included a passage on this. See highlight ton page 13 (lines 5–8) and supplemental tables 1 and 2..

Response: Below is a comparison of the demographics between the test and control groups. This table appears in the paper as Supplemental Table 1.

Comparison Between Test and Control Group Demographics

Test Group Control Group p-value

Age 42.11 41.68 .7

Gender .25

Male 213 199

Female 216 227

Non-binary 0 2

Ideology .27

Leans Independent 133 113

Leans Liberal 125 148

Leans Conservative 132 133

Other 39 34

Income .98

Less than $50,000 136 134

Less than $50,000 118 117

More than 100.001 175 177

Test group N = 429

Control group N = 428

Age denotes t-test; gender, ideology, and income denote chi-square test

- if it is the case that your two groups were comprised of different demographic makeups, I'm curious how that might influence your results, since there isn't very much discussion of demographics in the manuscript. Specifically, I'm wondering about ideological differences in news literacy and vulnerability to clickbait, since there's ample research that deals with formation of political echo chambers and political misinformation. if there were notable demographic differences on your literacy or other metrics, perhaps this is something that could be discussed in a short appendix.

Response: I have now included the demographics variables in the regression analysis. Only age and income returned main effects. I contextualize the effect of the demographics variables in the discussion section. See highlight on page 29 (lines 4–16).

- your introduction is at times a bit repetitive, such as for example in the second paragraph of your "effects of clickbait" section, where you mention the definition of forward-referencing despite already describing what forward referencing is in your opening paragraph. I would suggest tightening up the writing a bit here and make sure you aren't repeating yourself, but this is a very minor thing

Response: I have revised this part to reduce the repetitiveness. See highlight on pages 4 (lines 12–21).

All in all, I think this is a strong manuscript that would make a great candidate for publication.

Attachment Submitted filename: Responses to Reviewers.docx

# Peer review: Decision Letter 1

**DOI:** 10.1371/journal.pone.0359401.r003

**Authors:** Robin Haunschild

6 May 2026

-->SHOCKING! College Professor Reveals Hidden Secrets of News Headlines. Can You Guess?

PLOS One?

Dear Dr. Wasike,

Thank you for submitting your manuscript to PLOS ONE. After careful consideration, we feel that it has merit but does not fully meet PLOS ONE’s publication criteria as it currently stands. Therefore, we invite you to submit a revised version of the manuscript that addresses the points raised during the review process.For Lab, Study and Registered Report Protocols: These article types are not expected to include results but may include pilot data.

Please submit your revised manuscript by Jun 20 2026 11:59PM. If you will need more time than this to complete your revisions, please reply to this message or contact the journal office at plosone@plos.org. When you're ready to submit your revision, log on to https://www.editorialmanager.com/pone/ and select the 'Submissions Needing Revision' folder to locate your manuscript file.

- A letter that responds to each point raised by the academic editor and reviewer(s). You should upload this letter as a separate file labeled 'Response to Reviewers'.
- A marked-up copy of your manuscript that highlights changes made to the original version. You should upload this as a separate file labeled 'Revised Manuscript with Track Changes'.
- An unmarked version of your revised paper without tracked changes. You should upload this as a separate file labeled 'Manuscript'.

If you would like to make changes to your financial disclosure, please include your updated statement in your cover letter. Guidelines for resubmitting your figure files are available below the reviewer comments at the end of this letter.

If applicable, we recommend that you deposit your laboratory protocols in protocols.io to enhance the reproducibility of your results. Protocols.io assigns your protocol its own identifier (DOI) so that it can be cited independently in the future. For instructions see: https://journals.plos.org/plosone/s/submission-guidelines#loc-laboratory-protocols. Additionally, PLOS ONE offers an option for publishing peer-reviewed Lab Protocol articles, which describe protocols hosted on protocols.io. Read more information on sharing protocols at https://plos.org/protocols?utm_medium=editorial-email&utm_source=authorletters&utm_campaign=protocols.

As the corresponding author, your ORCID iD is verified in the submission system and will appear in the published article. PLOS supports the use of ORCID, and we encourage all coauthors to register for an ORCID iD and use it as well. Please encourage your coauthors to verify their ORCID iD within the submission system before final acceptance, as unverified ORCID iDs will not appear in the published article. *Only*  the individual author can complete the verification step; PLOS staff *cannot*  verify ORCID iDs on behalf of authors.

We look forward to receiving your revised manuscript.

Kind regards,

Robin Haunschild

Academic Editor

PLOS One

Journal Requirements:

If the reviewer comments include a recommendation to cite specific previously published works, please review and evaluate these publications to determine whether they are relevant and should be cited. There is no requirement to cite these works unless the editor has indicated otherwise.

[Note: HTML markup is below. Please do not edit.]

Reviewers' comments:

Reviewer's Responses to Questions

**Comments to the Author**

Reviewer #1: All comments have been addressed

Reviewer #2: All comments have been addressed

Reviewer #3: All comments have been addressed

Reviewer #5: All comments have been addressed

Reviewer #6: All comments have been addressed

Reviewer #7: (No Response)

Reviewer #8: All comments have been addressed

**********

2. Is the manuscript technically sound, and do the data support the conclusions?

Reviewer #1: Yes

Reviewer #2: Partly

Reviewer #3: Yes

Reviewer #5: Yes

Reviewer #6: Yes

Reviewer #7: No

Reviewer #8: Yes

**********

3. Has the statistical analysis been performed appropriately and rigorously?

Reviewer #1: Yes

Reviewer #2: N/A

Reviewer #3: I Don't Know

Reviewer #5: Yes

Reviewer #6: Yes

Reviewer #7: No

Reviewer #8: Yes

**********

4. Have the authors made all data underlying the findings in their manuscript fully available?

The PLOS Data policy

Reviewer #1: (No Response)

Reviewer #2: Yes

Reviewer #3: Yes

Reviewer #5: Yes

Reviewer #6: Yes

Reviewer #7: Yes

Reviewer #8: Yes

**********

5. Is the manuscript presented in an intelligible fashion and written in standard English?

Reviewer #1: Yes

Reviewer #2: Yes

Reviewer #3: Yes

Reviewer #5: Yes

Reviewer #6: Yes

Reviewer #7: Yes

Reviewer #8: Yes

**********

Reviewer #1: Overall, my comments are addressed. Limitations section can be expanded a bit to include a point about having more diverse and comprehensive manipulation check questions in future research. I read other reviewers’ thoughtful and detailed comments and it looks like the revised manuscript improved a lot in clarity, transparency, and methodological details a lot. One comment I have about demographics is that pre-test balance checks shows that randomization worked, hence I think it is unnecessary to have demographic covariates in the analysis, but they can still be retained. This can be noted somewhere in analytical strategy that these covariates are added as exploratory as they are not required for experimental comparison. I think this paper is a good addition to the literature.

Reviewer #2: Thank you for the revised manuscript and the detailed point-by-point responses. The authors have addressed most of my previous concerns in a serious and constructive manner, and the manuscript has been improved accordingly. In its current form, I believe the paper is generally acceptable for publication.

That said, I would like to retain two reservations for the record.

I appreciate the author's effort to justify the current title by citing prior studies on clickbait that used attention-grabbing wording. However, I remain unconvinced that these examples are fully comparable to the present manuscript title. Several of the cited titles are either more restrained in tone, explicitly framed within an academic subtitle, or use clickbait-like language in a self-referential and clearly scholarly way. By contrast, the current manuscript title appears to reproduce clickbait rhetoric more directly, and thus more closely resembles the very discursive form that the paper critically examines. For this reason, I do not think the cited precedents fully resolve the concern. My reservation is therefore not about the mere presence of an attention-grabbing phrase, but about the degree and manner in which the title adopts clickbait conventions in an academic context.

Second, I appreciate that the authors have now acknowledged the issues of news issue domain and headline valence or emotional orientation in the limitations section. Given the stated scope of the study, this is an acceptable way to handle these factors at this stage. However, I would still note that these are not merely peripheral matters, they may shape clickbait perception and detection in meaningful ways and therefore affect the broader interpretability of the findings.

Overall, these remaining concerns are reservations rather than fundamental objections. The manuscript has made a contribution, and I consider it acceptable for publication.

Reviewer #3: Thanks for your revisions, which have addressed my concerns.

The last suggestion you might consider:

Although the title of the research article allows for its creativity, it is essential not only to consider its appeal to the audience, but also to maintain the nature of the research, which aligns with the nature of this Journal. Therefore, I would suggest that the author revisit the title and choose a more appropriate one.

Reviewer #5: (No Response)

Reviewer #6: Dear author,

I have read the revised version of your manuscript with great interest. I went through all previous comments of each reviewer. I remarked substantial improvements have been made. Thus, I conclude that the manuscript is good enough to be published in its current form.

Regards

Reviewer #7: I appreciate the high level of effort this revision represents and appreciate all of the engagement among scholars that I see in the lengthy review record. This effort has much improved the work. I prepared my review from the unhighlighted version to try to offer a fresher perspective.

Ultimately I find that several of my key concerns have not been addressed by the revision. My concerns are with respect to the results and their interpretation, as well as the concepts under study. Unfortunately and with due respect to all the labor in evidence, I cannot yet recommend publication given these gaps.

1) Interpretation of results.

To the extent that clickbait training only functions in the limited scope described in the abstract (i.e. when only clickbait is present), I'm not persuaded by the evidence presented here that such training is beneficial. News consumers are faced with an environment that mixes clickbait with non-clickbait, the exception being perhaps the weird clickbait / advertising section one finds at the bottom of even legitimate sites.

I also note that the correlations are relatively small to negligible and the coefficients are all quite small. Descriptions of the practical significance of these effect sizes would be very useful as the units are constructed within the instrument.

2 ) Concepts under study

I find that I am still concerned about the lack of a conceptual map or a way to relate these ideas to one another, and the resulting statistical problems in interpretation. To be clear, this is a fundamental validity question, rather than one of reader clarity.

The text argues that personal locus is "a measure of media literacy associated with news literacy" and misinformation susceptibility; news literacy is also described as a "subset" of media literacy, and "associated with" the 4 personal locus variables. The study tests 5 variables -- the 4 locus variables and a measure of news literacy. However, conceptually these ideas are not presented as independent from one another.

This seems like a source of either multicollinearity, or otherwise turns the locus variables into controls (potentially partial / noisy ones) for news literacy, which makes interpretation challenging and doesn't support the interpretation in use. It does look like the multicollinearity is not bad given the correlations in table 1, but the issue of noisy / bad controls remains -- because then when interpreting the coefficients in table 2, one would say, "a 1-unit increase in news literacy is associated with a .22 unit increase in clickbait detection, holding signal detection, reflective thinking, TfA, and DfI constant", or "two people who are identical in their personal locus but who differ by one unit in their news literacy...". My question is, given the conceptual map as I understand it from the text, if the locus variables are themselves part of news literacy, how should we interpret the regression results, or if the 4 personal locus variables serve as a measure of news literacy, why are both the personal locus variables and news literacy serving as independent variables in the same regression?

Then again, later this is also described as a finding of the study rather than an element of the conceptual framework. I am not sure why this is framed as a finding, however; it seems that news literacy as a subset of media literacy would inevitably be associated with the same factors as its parent concept.

3) Document preparation notes

- page 3 line 9, punctuation

- page 3 line 15, unclear pronoun reference (them)

- page 14 line 2, stray comma, advice -> advise

- page 28 line 7 (headiness)

- some generic statements (e.g., "Notable findings emerged." and "Because hardly any research has examined this angle, let alone in the context of clickbait detection, this study uniquely adds to knowledge.")

Reviewer #8: I believe that the author has adequately addressed mine and the other reviewers’ comments and that the manuscript is now suitable for publication.

**********

what does this mean?). If published, this will include your full peer review and any attached files.

If you choose “no”, your identity will remain anonymous but your review may still be made public.

**Do you want your identity to be public for this peer review?**  For information about this choice, including consent withdrawal, please see our Privacy Policy

Reviewer #1: No

Reviewer #2: No

Reviewer #3: No

Reviewer #5: **Yes:**  Denise Coberley

Reviewer #6: **Yes:**  Claudel Mombeuil

Reviewer #7: No

Reviewer #8: No

**********

[NOTE: If reviewer comments were submitted as an attachment file, they will be attached to this email and accessible via the submission site. Please log into your account, locate the manuscript record, and check for the action link "View Attachments". If this link does not appear, there are no attachment files.]

To ensure your figures meet our technical requirements, please review our figure guidelines: https://journals.plos.org/plosone/s/figures

You may also use PLOS’s free figure tool, NAAS, to help you prepare publication quality figures: https://journals.plos.org/plosone/s/figures#loc-tools-for-figure-preparation.

NAAS will assess whether your figures meet our technical requirements by comparing each figure against our figure specifications.

# Author comment: Author response to Decision Letter 2

**DOI:** 10.1371/journal.pone.0359401.r004

13 May 2026

6. Review Comments to the Author

Please use the space provided to explain your answers to the questions above. You may also include additional comments for the author, including concerns about dual publication, research ethics, or publication ethics. (Please upload your review as an attachment if it exceeds 20,000 characters)

Reviewer #1: Overall, my comments are addressed. Limitations section can be expanded a bit to include a point about having more diverse and comprehensive manipulation check questions in future research. I read other reviewers’ thoughtful and detailed comments and it looks like the revised manuscript improved a lot in clarity, transparency, and methodological details a lot. One comment I have about demographics is that pre-test balance checks shows that randomization worked, hence I think it is unnecessary to have demographic covariates in the analysis, but they can still be retained. This can be noted somewhere in analytical strategy that these covariates are added as exploratory as they are not required for experimental comparison. I think this paper is a good addition to the literature.

Response: I have added a note about the manipulation check in the limitations section. See highlight on page 32.

Response: I have also added a note about using the demographic variables as controls in the Statistical Analysis section. See the highlight on page 20.

Reviewer #2: Thank you for the revised manuscript and the detailed point-by-point responses. The authors have addressed most of my previous concerns in a serious and constructive manner, and the manuscript has been improved accordingly. In its current form, I believe the paper is generally acceptable for publication.

That said, I would like to retain two reservations for the record.

I appreciate the author's effort to justify the current title by citing prior studies on clickbait that used attention-grabbing wording. However, I remain unconvinced that these examples are fully comparable to the present manuscript title. Several of the cited titles are either more restrained in tone, explicitly framed within an academic subtitle, or use clickbait-like language in a self-referential and clearly scholarly way. By contrast, the current manuscript title appears to reproduce clickbait rhetoric more directly, and thus more closely resembles the very discursive form that the paper critically examines. For this reason, I do not think the cited precedents fully resolve the concern. My reservation is therefore not about the mere presence of an attention-grabbing phrase, but about the degree and manner in which the title adopts clickbait conventions in an academic context.

Second, I appreciate that the authors have now acknowledged the issues of news issue domain and headline valence or emotional orientation in the limitations section. Given the stated scope of the study, this is an acceptable way to handle these factors at this stage. However, I would still note that these are not merely peripheral matters, they may shape clickbait perception and detection in meaningful ways and therefore affect the broader interpretability of the findings.

Overall, these remaining concerns are reservations rather than fundamental objections. The manuscript has made a contribution, and I consider it acceptable for publication.

Response: I have since revised the title. Even though the kicker still reads like clickbait (for purposes of illustration), I have added more to the title to make it suitable for a scholarly paper.

Reviewer #3: Thanks for your revisions, which have addressed my concerns.

The last suggestion you might consider:

Although the title of the research article allows for its creativity, it is essential not only to consider its appeal to the audience, but also to maintain the nature of the research, which aligns with the nature of this Journal. Therefore, I would suggest that the author revisit the title and choose a more appropriate one.

Response: Similar to my response above, I have since revised the title. Even though the kicker still reads like clickbait (for purposes of illustration), I have added more to the title to make it suitable for a scholarly paper.

Reviewer #5: (No Response)

Reviewer #6: Dear author,

I have read the revised version of your manuscript with great interest. I went through all previous comments of each reviewer. I remarked substantial improvements have been made. Thus, I conclude that the manuscript is good enough to be published in its current form.

Regards

Reviewer #7: I appreciate the high level of effort this revision represents and appreciate all of the engagement among scholars that I see in the lengthy review record. This effort has much improved the work. I prepared my review from the unhighlighted version to try to offer a fresher perspective.

Ultimately I find that several of my key concerns have not been addressed by the revision. My concerns are with respect to the results and their interpretation, as well as the concepts under study. Unfortunately and with due respect to all the labor in evidence, I cannot yet recommend publication given these gaps.

1) Interpretation of results.

To the extent that clickbait training only functions in the limited scope described in the abstract (i.e. when only clickbait is present), I'm not persuaded by the evidence presented here that such training is beneficial. News consumers are faced with an environment that mixes clickbait with non-clickbait, the exception being perhaps the weird clickbait / advertising section one finds at the bottom of even legitimate sites.

I also note that the correlations are relatively small to negligible and the coefficients are all quite small. Descriptions of the practical significance of these effect sizes would be very useful as the units are constructed within the instrument.

Response: As I mentioned in the paper, these results may be because this was a cross-sectional study where subjects were exposed to a single session of clickbait detection training. A longitudinal study with multiple training sessions could have produced more robust results. I have added a note about this practicality in the implications section – see highlight on page 31.

2 ) Concepts under study

I find that I am still concerned about the lack of a conceptual map or a way to relate these ideas to one another, and the resulting statistical problems in interpretation. To be clear, this is a fundamental validity question, rather than one of reader clarity.

The text argues that personal locus is "a measure of media literacy associated with news literacy" and misinformation susceptibility; news literacy is also described as a "subset" of media literacy, and "associated with" the 4 personal locus variables. The study tests 5 variables -- the 4 locus variables and a measure of news literacy. However, conceptually these ideas are not presented as independent from one another.

Response: My rationale is that extant research suggests that these variables are interrelated, not independent, as the reviewer mentions, but no one has examined these associations, hence the uniqueness of the study. I feel that I have adequately discussed the literature suggesting potential associations, and the results support this conceptualization.

This seems like a source of either multicollinearity, or otherwise turns the locus variables into controls (potentially partial / noisy ones) for news literacy, which makes interpretation challenging and doesn't support the interpretation in use. It does look like the multicollinearity is not bad given the correlations in table 1, but the issue of noisy / bad controls remains -- because then when interpreting the coefficients in table 2, one would say, "a 1-unit increase in news literacy is associated with a .22 unit increase in clickbait detection, holding signal detection, reflective thinking, TfA, and DfI constant", or "two people who are identical in their personal locus but who differ by one unit in their news literacy...". My question is, given the conceptual map as I understand it from the text, if the locus variables are themselves part of news literacy, how should we interpret the regression results, or if the 4 personal locus variables serve as a measure of news literacy, why are both the personal locus variables and news literacy serving as independent variables in the same regression?

Response: The four personal locus variables do not measure news literacy, but I argue that they are adjacent to news literacy. I think the confusion arose because of how I introduced media literacy, news literacy, and personal locus. I have revised this section for clarity by emphasizing the chronological order in which these interrelated concepts were introduced. Media literacy in 1992, personal locus in 2004, and news literacy in 2008. In brief, media literacy is the broader concept that was proposed in 1992. Potter reimagined media literacy in 2004, when he introduced personal locus as a key component of media literacy. Allan Miller later introduced news literacy in 2008 – see highlight on page 8.

Then again, later this is also described as a finding of the study rather than an element of the conceptual framework. I am not sure why this is framed as a finding, however; it seems that news literacy as a subset of media literacy would inevitably be associated with the same factors as its parent concept.

Response: I hope my response above clears this confusion.

3) Document preparation notes

Response: I have corrected the issues listed below.

- page 3 line 9, punctuation

- page 3 line 15, unclear pronoun reference (them)

- page 14 line 2, stray comma, advice -> advise

- page 28 line 7 (headiness) – I have revised this statement. See highlight on page 28.

- some generic statements (e.g., "Notable findings emerged." and "Because hardly any research has examined this angle, let alone in the context of clickbait detection, this study uniquely adds to knowledge.") – I have revised this statement. See highlight on page 4.

Reviewer #8: I believe that the author has adequately addressed mine and the other reviewers’ comments and that the manuscript is now suitable for publication.

Attachment Submitted filename: Response to Reviiwer Comments.docx

# Peer review: Decision Letter 2

**DOI:** 10.1371/journal.pone.0359401.r005

**Authors:** Robin Haunschild

16 Jul 2026

PONE-D-26-01307R2

SHOCKING! College Professor Reveals Hidden Secrets of News Headlines. Can You Guess? Examining the Efficacy of Clickbait Detection Training and News Literacy

PLOS One?

Dear Dr. Wasike,

Thank you for submitting your manuscript to PLOS ONE. After careful consideration, we feel that it has merit but does not fully meet PLOS ONE’s publication criteria as it currently stands. Therefore, we invite you to submit a revised version of the manuscript that addresses the points raised during the review process.

Please submit your revised manuscript by Aug 30 2026 11:59PM. If you will need more time than this to complete your revisions, please reply to this message or contact the journal office at plosone@plos.org. When you're ready to submit your revision, log on to https://www.editorialmanager.com/pone/ and select the 'Submissions Needing Revision' folder to locate your manuscript file.

- A letter that responds to each point raised by the academic editor and reviewer(s). You should upload this letter as a separate file labeled 'Response to Reviewers'.
- A marked-up copy of your manuscript that highlights changes made to the original version. You should upload this as a separate file labeled 'Revised Manuscript with Track Changes'.
- An unmarked version of your revised paper without tracked changes. You should upload this as a separate file labeled 'Manuscript'.

If you would like to make changes to your financial disclosure, please include your updated statement in your cover letter. Guidelines for resubmitting your figure files are available below the reviewer comments at the end of this letter.

If applicable, we recommend that you deposit your laboratory protocols in protocols.io to enhance the reproducibility of your results. Protocols.io assigns your protocol its own identifier (DOI) so that it can be cited independently in the future. For instructions see: https://journals.plos.org/plosone/s/submission-guidelines#loc-laboratory-protocols. Additionally, PLOS ONE offers an option for publishing peer-reviewed Lab Protocol articles, which describe protocols hosted on protocols.io. Read more information on sharing protocols at https://plos.org/protocols?utm_medium=editorial-email&utm_source=authorletters&utm_campaign=protocols.

As the corresponding author, your ORCID iD is verified in the submission system and will appear in the published article. PLOS supports the use of ORCID, and we encourage all coauthors to register for an ORCID iD and use it as well. Please encourage your coauthors to verify their ORCID iD within the submission system before final acceptance, as unverified ORCID iDs will not appear in the published article. *Only*  the individual author can complete the verification step; PLOS staff *cannot*  verify ORCID iDs on behalf of authors.

We look forward to receiving your revised manuscript.

Kind regards,

Robin Haunschild

Academic Editor

PLOS One

Journal Requirements:

If the reviewer comments include a recommendation to cite specific previously published works, please review and evaluate these publications to determine whether they are relevant and should be cited. There is no requirement to cite these works unless the editor has indicated otherwise.

Please review your reference list to ensure that it is complete and correct. If you have cited papers that have been retracted, please include the rationale for doing so in the manuscript text, or remove these references and replace them with relevant current references. Any changes to the reference list should be mentioned in the rebuttal letter that accompanies your revised manuscript. If you need to cite a retracted article, indicate the article’s retracted status in the References list and also include a citation and full reference for the retraction notice.

[Note: HTML markup is below. Please do not edit.]

Reviewers' comments:

Reviewer's Responses to Questions

**Comments to the Author**

Reviewer #7: (No Response)

**********

2. Is the manuscript technically sound, and do the data support the conclusions?

Reviewer #7: Yes

**********

3. Has the statistical analysis been performed appropriately and rigorously?

Reviewer #7: Yes

**********

4. Have the authors made all data underlying the findings in their manuscript fully available?

The PLOS Data policy

Reviewer #7: Yes

**********

5. Is the manuscript presented in an intelligible fashion and written in standard English?

Reviewer #7: No

**********

Reviewer #7: I have only some document preparation notes:

Probably this should have 'One,' 'Two,' and 'Three' for the three negative effects; to me this reads like four items, and jumps from First to Third and then has two un-numbered items:

10 However, research indicates that question-based headlines also

11 elicit three negative effects that traditional headlines do not [4]. First, such headlines elicit

12 perceptions of untrustworthiness, confusion, impropriety, and the lack of credibility and

13 seriousness. They also increase the anticipation that news articles following the headlines will

14 elicit the same negative reactions. Third, question-based clickbait headlines reduce engagement

15 with the news via commenting, sharing, or posting reactions such as likes. Other research shows

16 that stories linked to question-based clickbait headlines also receive less engagement online [27].

17 However, readers are more likely to click on question-based clickbait headlines than traditional

18 headlines with no questions [28] even though other research shows that question-based clickbait

19 headlines reduce a reader’s comprehension of accompanying stories [29].

I'm not sure these organizations are news agencies per se? The term typically refers to wire services like the Associated Press:

18 These headlines were published by news agencies such as the BBC,

19 The Sun, and Upworthy.

This text reads as redundant:

"Like with clickbait, valence and affect also make a difference. Here, the valence and context with which a headline is written also matter."

Stray text:

"and more credible l [52]"

Typo:

"hypothesis 6 predicts that clickbait raining"

**********

what does this mean?). If published, this will include your full peer review and any attached files.

If you choose “no”, your identity will remain anonymous but your review may still be made public.

**Do you want your identity to be public for this peer review?**  For information about this choice, including consent withdrawal, please see our Privacy Policy

Reviewer #7: No

**********

[NOTE: If reviewer comments were submitted as an attachment file, they will be attached to this email and accessible via the submission site. Please log into your account, locate the manuscript record, and check for the action link "View Attachments". If this link does not appear, there are no attachment files.]

To ensure your figures meet our technical requirements, please review our figure guidelines: https://journals.plos.org/plosone/s/figures

You may also use PLOS’s free figure tool, NAAS, to help you prepare publication quality figures: https://journals.plos.org/plosone/s/figures#loc-tools-for-figure-preparation.

NAAS will assess whether your figures meet our technical requirements by comparing each figure against our figure specifications.

# Author comment: Author response to Decision Letter 3

**DOI:** 10.1371/journal.pone.0359401.r006

17 Jul 2026

Comments to the Author

1. If the authors have adequately addressed your comments raised in a previous round of review and you feel that this manuscript is now acceptable for publication, you may indicate that here to bypass the “Comments to the Author” section, enter your conflict of interest statement in the “Confidential to Editor” section, and submit your "Accept" recommendation.

Reviewer #7: (No Response)

Response: N/A

2. Is the manuscript technically sound, and do the data support the conclusions?

The manuscript must describe a technically sound piece of scientific research with data that supports the conclusions. Experiments must have been conducted rigorously, with appropriate controls, replication, and sample sizes. The conclusions must be drawn appropriately based on the data presented.

Reviewer #7: Yes

Response: N/A

3. Has the statistical analysis been performed appropriately and rigorously?

Reviewer #7: Yes

Response: N/A

4. Have the authors made all data underlying the findings in their manuscript fully available?

The PLOS Data policy requires authors to make all data underlying the findings described in their manuscript fully available without restriction, with rare exception (please refer to the Data Availability Statement in the manuscript PDF file). The data should be provided as part of the manuscript or its supporting information, or deposited to a public repository. For example, in addition to summary statistics, the data points behind means, medians and variance measures should be available. If there are restrictions on publicly sharing data—e.g. participant privacy or use of data from a third party—those must be specified.

Reviewer #7: Yes

Response: N/A

5. Is the manuscript presented in an intelligible fashion and written in standard English?

PLOS ONE does not copyedit accepted manuscripts, so the language in submitted articles must be clear, correct, and unambiguous. Any typographical or grammatical errors should be corrected at revision, so please note any specific errors here.

Reviewer #7: No

Response: Please see response below

6. Review Comments to the Author

Please use the space provided to explain your answers to the questions above. You may also include additional comments for the author, including concerns about dual publication, research ethics, or publication ethics. (Please upload your review as an attachment if it exceeds 20,000 characters)

Reviewer #7: I have only some document preparation notes:

Probably this should have 'One,' 'Two,' and 'Three' for the three negative effects; to me this reads like four items, and jumps from First to Third and then has two un-numbered items:

10 However, research indicates that question-based headlines also

11 elicit three negative effects that traditional headlines do not [4]. First, such headlines elicit

12 perceptions of untrustworthiness, confusion, impropriety, and the lack of credibility and

13 seriousness. They also increase the anticipation that news articles following the headlines will

14 elicit the same negative reactions. Third, question-based clickbait headlines reduce engagement

15 with the news via commenting, sharing, or posting reactions such as likes. Other research shows

16 that stories linked to question-based clickbait headlines also receive less engagement online [27].

17 However, readers are more likely to click on question-based clickbait headlines than traditional

18 headlines with no questions [28] even though other research shows that question-based clickbait

19 headlines reduce a reader’s comprehension of accompanying stories [29].

Response: I have rewritten this passage as suggested. See highlight on page 6.

I'm not sure these organizations are news agencies per se? The term typically refers to wire services like the Associated Press:

18 These headlines were published by news agencies such as the BBC,

19 The Sun, and Upworthy.

Response: This is a good observation. I have since replaced the phrase “news agency” with “news outlets.” See highlight on page 5.

This text reads as redundant:

"Like with clickbait, valence and affect also make a difference. Here, the valence and context with which a headline is written also matter."

Response: I have revised to only use valence. See highlight on pages 7–8.

Stray text:

" This explains why news-literate people are more likely to rate stories that carry real rather than generic photos and those with traditional rather than clickbait headlines as better written, more professional, better organized, and they also perceive them as more credible l [52]. l [52]"

Response: I did not understand this comment, but I have rewritten this passage as shown below. In the cited study, quality of writing, professionalism, organization, and credibility were measured as separate parameters.

This explains why news-literate people are more likely to rate stories that carry real rather than generic photos and those with traditional rather than clickbait headlines as better written, more professional, better organized, and they also perceive them as more credible l [52].

Typo:

"hypothesis 6 predicts that clickbait raining"

Response: Corrected – see highlight on page 12.

7. PLOS authors have the option to publish the peer review history of their article (what does this mean?). If published, this will include your full peer review and any attached files.

If you choose “no”, your identity will remain anonymous but your review may still be made public.

Do you want your identity to be public for this peer review? For information about this choice, including consent withdrawal, please see our Privacy Policy.

Reviewer #7: No

Response: N/A

Attachment Submitted filename: Response to Reviewer Comments.docx

# Peer review: Decision Letter 3

**DOI:** 10.1371/journal.pone.0359401.r007

**Authors:** Robin Haunschild

7 Sep 2026

PONE-D-26-01307R3

SHOCKING! College Professor Reveals Hidden Secrets of News Headlines. Can You Guess? Examining the Efficacy of Clickbait Detection Training and News Literacy

PLOS One?

Dear Dr. Wasike,

Thank you for submitting your manuscript to PLOS One. After careful consideration, we feel that it has merit but does not fully meet PLOS One’s publication criteria as it currently stands. Therefore, we invite you to submit a revised version of the manuscript that addresses the points raised during the review process.

Please submit your revised manuscript by Oct 22 2026 11:59PM. If you will need more time than this to complete your revisions, please reply to this message or contact the journal office at plosone@plos.org. When you're ready to submit your revision, log on to https://www.editorialmanager.com/pone/ and select the 'Submissions Needing Revision' folder to locate your manuscript file.

- A letter that responds to each point raised by the academic editor and reviewer(s). You should upload this letter as a separate file labeled 'Response to Reviewers'.
- A marked-up copy of your manuscript that highlights changes made to the original version. You should upload this as a separate file labeled 'Revised Manuscript with Track Changes'.
- An unmarked version of your revised paper without tracked changes. You should upload this as a separate file labeled 'Manuscript'.

If you would like to make changes to your financial disclosure, please include your updated statement in your cover letter. Guidelines for resubmitting your figure files are available below the reviewer comments at the end of this letter.

If applicable, we recommend that you deposit your laboratory protocols in protocols.io to enhance the reproducibility of your results. Protocols.io assigns your protocol its own identifier (DOI) so that it can be cited independently in the future. For instructions see: https://journals.plos.org/plosone/s/submission-guidelines#loc-laboratory-protocols. Additionally, PLOS One offers an option for publishing peer-reviewed Lab Protocol articles, which describe protocols hosted on protocols.io. Read more information on sharing protocols at https://plos.org/protocols?utm_medium=editorial-email&utm_source=authorletters&utm_campaign=protocols.

As the corresponding author, your ORCID iD is verified in the submission system and will appear in the published article. PLOS supports the use of ORCID, and we encourage all coauthors to register for an ORCID iD and use it as well. Please encourage your coauthors to verify their ORCID iD within the submission system before final acceptance, as unverified ORCID iDs will not appear in the published article. *Only*  the individual author can complete the verification step; PLOS staff *cannot*  verify ORCID iDs on behalf of authors.

We look forward to receiving your revised manuscript.

Kind regards,

Robin Haunschild

Academic Editor

PLOS One

Journal Requirements:

If the reviewer comments include a recommendation to cite specific previously published works, please review and evaluate these publications to determine whether they are relevant and should be cited. There is no requirement to cite these works unless the editor has indicated otherwise.

Please review your reference list to ensure that it is complete and correct. If you have cited papers that have been retracted, please include the rationale for doing so in the manuscript text, or remove these references and replace them with relevant current references. Any changes to the reference list should be mentioned in the rebuttal letter that accompanies your revised manuscript. If you need to cite a retracted article, indicate the article’s retracted status in the References list and also include a citation and full reference for the retraction notice.

[Note: HTML markup is below. Please do not edit.]

Reviewers' comments:

Reviewer's Responses to Questions

**Comments to the Author**

Reviewer #7: (No Response)

**********

2. Is the manuscript technically sound, and do the data support the conclusions?

Reviewer #7: Yes

**********

3. Has the statistical analysis been performed appropriately and rigorously?

Reviewer #7: Yes

**********

4. Have the authors made all data underlying the findings in their manuscript fully available?

The PLOS Data policy

Reviewer #7: Yes

**********

5. Is the manuscript presented in an intelligible fashion and written in standard English?

Reviewer #7: Yes

**********

Reviewer #7: All of my comments from the prior review have been handled with the exception of one -- I pointed out that the following contains stray text: "credible l [52]". The response received was that my comment was not understood. I am not sure how else to clarify, but I have no idea what "credible l" followed by a citation can possibly mean if it is not an error or omission of some kind. Apologies, truly, at prolonging the cycle of review. But this error does persist in the present version after the revision.

**********

what does this mean?). If published, this will include your full peer review and any attached files.

If you choose “no”, your identity will remain anonymous but your review may still be made public.

**Do you want your identity to be public for this peer review?**  For information about this choice, including consent withdrawal, please see our Privacy Policy

Reviewer #7: No

**********

[NOTE: If reviewer comments were submitted as an attachment file, they will be attached to this email and accessible via the submission site. Please log into your account, locate the manuscript record, and check for the action link "View Attachments". If this link does not appear, there are no attachment files.]

To ensure your figures meet our technical requirements, please review our figure guidelines: https://journals.plos.org/plosone/s/figures

You may also use PLOS’s free figure tool, NAAS, to help you prepare publication quality figures: https://journals.plos.org/plosone/s/figures#loc-tools-for-figure-preparation.

NAAS will assess whether your figures meet our technical requirements by comparing each figure against our figure specifications.

# Author comment: Author response to Decision Letter 4

**DOI:** 10.1371/journal.pone.0359401.r008

10 Sep 2026

Comments to the Author

1. If the authors have adequately addressed your comments raised in a previous round of review and you feel that this manuscript is now acceptable for publication, you may indicate that here to bypass the “Comments to the Author” section, enter your conflict of interest statement in the “Confidential to Editor” section, and submit your "Accept" recommendation.

Reviewer #7: (No Response)

Response: N/A

2. Is the manuscript technically sound, and do the data support the conclusions?

The manuscript must describe a technically sound piece of scientific research with data that supports the conclusions. Experiments must have been conducted rigorously, with appropriate controls, replication, and sample sizes. The conclusions must be drawn appropriately based on the data presented.

Reviewer #7: Yes

Response: N/A

3. Has the statistical analysis been performed appropriately and rigorously?

Reviewer #7: Yes

Response: N/A

4. Have the authors made all data underlying the findings in their manuscript fully available?

The PLOS Data policy requires authors to make all data underlying the findings described in their manuscript fully available without restriction, with rare exception (please refer to the Data Availability Statement in the manuscript PDF file). The data should be provided as part of the manuscript or its supporting information, or deposited to a public repository. For example, in addition to summary statistics, the data points behind means, medians and variance measures should be available. If there are restrictions on publicly sharing data—e.g. participant privacy or use of data from a third party—those must be specified.

Reviewer #7: Yes

Response: N/A

5. Is the manuscript presented in an intelligible fashion and written in standard English?

PLOS ONE does not copyedit accepted manuscripts, so the language in submitted articles must be clear, correct, and unambiguous. Any typographical or grammatical errors should be corrected at revision, so please note any specific errors here.

Reviewer #7: Yes

Response: N/A

6. Review Comments to the Author

Please use the space provided to explain your answers to the questions above. You may also include additional comments for the author, including concerns about dual publication, research ethics, or publication ethics. (Please upload your review as an attachment if it exceeds 20,000 characters)

Reviewer #7: All of my comments from the prior review have been handled with the exception of one -- I pointed out that the following contains stray text: "credible l [52]". The response received was that my comment was not understood. I am not sure how else to clarify, but I have no idea what "credible l" followed by a citation can possibly mean if it is not an error or omission of some kind. Apologies, truly, at prolonging the cycle of review. But this error does persist in the present version after the revision.

Response: I had not noticed the extra character after the word “credible” in my initial review, hence my confusion. I have since deleted that character. See highlight on page 9 line 15.

7. PLOS authors have the option to publish the peer review history of their article (what does this mean?). If published, this will include your full peer review and any attached files.

If you choose “no”, your identity will remain anonymous but your review may still be made public.

Do you want your identity to be public for this peer review? For information about this choice, including consent withdrawal, please see our Privacy Policy.

Reviewer #7: No

Response: N/A

Attachment Submitted filename: Response_to_Reviewer_Comments_auresp_4.docx

# Editor report: Decision Letter 4

**DOI:** 10.1371/journal.pone.0359401.r009

**Authors:** Robin Haunschild

15 Sep 2026

SHOCKING! College Professor Reveals Hidden Secrets of News Headlines. Can You Guess? Examining the Efficacy of Clickbait Detection Training and News Literacy

PONE-D-26-01307R4

Dear Dr. Wasike,

We’re pleased to inform you that your manuscript has been judged scientifically suitable for publication and will be formally accepted for publication once it meets all outstanding technical requirements.

Within one week, you’ll receive an e-mail detailing the required amendments. When these have been addressed, you’ll receive a formal acceptance letter and your manuscript will be scheduled for publication.

An invoice will be generated when your article is formally accepted. Please note, if your institution has a publishing partnership with PLOS and your article meets the relevant criteria, all or part of your publication costs will be covered. Please make sure your user information is up-to-date by logging into Editorial Manager at Editorial Manager® and clicking the ‘Update My Information' link at the top of the page. For questions related to billing, please contact billing support.

If your institution or institutions have a press office, please notify them about your upcoming paper to help maximize its impact. If they’ll be preparing press materials, please inform our press team as soon as possible -- no later than 48 hours after receiving the formal acceptance. Your manuscript will remain under strict press embargo until 2 pm Eastern Time on the date of publication. For more information, please contact onepress@plos.org.

Kind regards,

Robin Haunschild

Academic Editor

PLOS One

Additional Editor Comments (optional):

Reviewers' comments:

# Editor report: Acceptance letter

**DOI:** 10.1371/journal.pone.0359401.r010

**Authors:** Robin Haunschild

PONE-D-26-01307R4

PLOS One

Dear Dr. Wasike,

I'm pleased to inform you that your manuscript has been deemed suitable for publication in PLOS One. Congratulations! Your manuscript is now being handed over to our production team.

At this stage, our production department will prepare your paper for publication. This includes ensuring the following:

* All references, tables, and figures are properly cited

* All relevant supporting information is included in the manuscript submission,

* There are no issues that prevent the paper from being properly typeset

You will receive further instructions from the production team, including instructions on how to review your proof when it is ready. Please keep in mind that we are working through a large volume of accepted articles, so please give us a few days to review your paper and let you know the next and final steps.

Lastly, if your institution or institutions have a press office, please let them know about your upcoming paper now to help maximize its impact. If they'll be preparing press materials, please inform our press team within the next 48 hours. Your manuscript will remain under strict press embargo until 2 pm Eastern Time on the date of publication. For more information, please contact onepress@plos.org.

You will receive an invoice from PLOS for your publication fee after your manuscript has reached the completed accept phase. If you receive an email requesting payment before acceptance or for any other service, this may be a phishing scheme. Learn how to identify phishing emails and protect your accounts at https://explore.plos.org/phishing.

If we can help with anything else, please email us at customercare@plos.org.

Thank you for submitting your work to PLOS One and supporting open access.

Kind regards,

PLOS One Editorial Office Staff

on behalf of

Dr. Robin Haunschild

Academic Editor

PLOS One
